# Drug-induced metabolic remodeling promotes antimicrobial activity in macrophages and extends longevity in vivo

**DOI:** 10.26508/lsa.202603852

**Published:** 2026-09-17

**Authors:** Claudia Hollmann, Eun Chan Park, Zheng Liu, Faye Nourollahi, Emīls Edgars Bašēns, Jonathan Dietz, Trushnal S Waghmare, Mehul Vora, Georgy Nikolayshvili, Nanci S Kane, Tongbin Li, Fabian Imdahl, Kay F Macleod, Zaiga Nora-Krukle, Mohamed M Elashiry, Christopher Rongo, Bhupesh K Prusty

**Affiliations:** 1 Institute of Virology and Immunobiology, University of Würzburg , Würzburg, Germany; 2 The Waksman Institute, Rutgers The State University of New Jersey , Piscataway, NJ, USA; 3 Institute of Microbiology and Virology, Riga Stradiņš University , Riga, Latvia; 4 ModOmics Ltd, Southampton, UK; 5 AccuraScience, Johnston, IA, USA; 6 Helmholtz Institute for RNA-based Infection Research, Würzburg, Germany; 7 The Ben May Department for Cancer Research, The University of Chicago , Chicago, IL, USA; 8 Department of Endodontics, Dental College of Georgia, Augusta University , Augusta, GA, USA

## Abstract

Multiple immune mechanisms must be coordinated to defend against a broad range of pathogens; however, the mechanisms by which broad-spectrum antipathogens act remain largely elusive. Here, we used systems biology approaches to understand the organization of human immune cells at the single-cell level and their reorganization in response to K21, a silane derivative effective against viral, bacterial, and fungal infections. K21 effectively reduced the infectivity of *Enterococcus faecalis* within macrophages while improving bacterial phagocytosis by human monocyte–derived macrophages, in a manner similar to that of known mitophagy inducers. K21 induced pro-inflammatory pathways in M1 and M2 macrophages without altering cytokine secretion, decreased a specific subtype of M1 macrophages and M2c macrophages, and improved mitochondrial health by enhancing mitochondrial recycling via mitophagy. Similar treatment of the model organism *C. elegans* induced mitophagy and extended lifespan, suggesting an evolutionarily conserved mechanism. Our work demonstrates that a drug that remodels mitochondrial metabolism can reshape the immune cell repertoire, potentially aiding the development of more effective antimicrobials and helping to prevent the emergence of drug-resistant pathogens.

## Abstract (precis)

A potent antimicrobial promotes mitochondrial fission and mitophagy, boosting the effectiveness of macrophages to defend against bacterial infection and promoting lifespan extension in *C. elegans*.

## Introduction

Macrophages are the key mediators of the innate immune system, which provides an early line of defense against pathogens that breach barrier tissues to enter the body. Macrophages exhibit immense variability and plasticity in their functions and differentiation, enabling them to respond to a broad range of pathogens, including viruses, bacteria, and fungi. The ability of macrophages to dynamically alter their functional, morphological, and metabolic properties to adapt to diverse microenvironments and various stages of infection and healing is termed macrophage polarization (Kolliniati et al, 2022). Naïve macrophages (non-polarized, referred to as M0) have a relatively quiescent metabolism that relies more on fatty acid beta-oxidation than glycolysis for energy production (Kelly & O’Neill, 2015). M0 macrophages can differentiate into polarized M1 (classically activated) macrophages or polarized M2 (alternatively activated macrophages) depending on their exposure to specific TLR agonists and cytokines (Murray & Wynn, 2011), with the specific macrophage polarization phenotype dictating whether the innate immune response is antimicrobial and pro-inflammatory (for M1), or more anti-inflammatory, and thus, favorable for tissue repair (for M2). Metabolic differences are important aspects of M1 versus M2 polarization, as M1 macrophage metabolism is characterized by elevated glycolysis, pentose phosphate pathway (PPP) flux, and fatty acid synthesis, whereas M2 macrophage metabolism is characterized by elevated fatty acid oxidation and oxidative phosphorylation (OXPHOS) (Galván-Peña & O’Neill, 2014; El Kasmi & Stenmark, 2015; Jha et al, 2015; Kumar et al, 2024). Indeed, manipulating macrophage metabolism (glycolysis, mitochondrial OXPHOS, TCA intermediates, fatty acid, and amino-acid pathways) through either genetic or pharmacologic interventions can shift macrophage polarization between M1 and M2 phenotypes in vitro and in vivo (Galván-Peña & O’Neill, 2014; De Santa et al, 2019; Guo et al, 2021; Dussold et al, 2024). Metabolic reprogramming in macrophages influences mitochondrial dynamics and mitophagy, which, in turn, shape macrophage polarization and functional differentiation toward M1 or M2 phenotypes (Li et al, 2025). This metabolic reprogramming is a crucial aspect of macrophage functional plasticity; however, we lack a comprehensive understanding of how these metabolic changes are mediated and how to regulate them to provide therapeutic benefits.

Traditionally human monocyte–derived macrophage (HMDM) differentiation and polarization has been analyzed through a combination of cytokine-based stimulatory factor treatment that induces naïve differentiation and polarization (e.g., GM-CSF and M-CSF to induce M0 GM-CSF–derived HMDMs and M0 M-CSF–derived HMDMs, which for simplicity we will term M1 and M2 naïve macrophages, respectively), followed by additional stimulatory cytokine treatment that induces macrophage activation (e.g., LPS/IFN-γ, IL-4, and TGF-β to induce activated M1, M2a, and M2c macrophages, respectively). Polarization is monitored by profiling secreted cytokines (e.g., IL-12 and IL-10 for M1 and M2, respectively) and flow cytometry analysis of cell-surface proteins (e.g., CD80 and CD206 for M1 and M2, respectively), yet these approaches do not capture the full spectrum of macrophage differentiation and adaptability. Moreover, macrophage functional diversity is influenced by the tissue in which the macrophage populations are found (e.g., wounds, tumor-associated macrophages, or TAMs), and the presence of stromal cells like fibroblasts can influence cytokine secretion and the expression of cell-surface markers (Ozdemir et al, 2019; Buechler et al, 2021; Mohammad-Hasani et al, 2026). Similarly, the co-incubation of monocytes and macrophages with other immune cells can reduce cytokine secretion and cell-surface markers such as CD80 (Ma et al, 2015; Ozdemir et al, 2019; Mohammad-Hasani et al, 2026). Indeed, single-cell RNA sequencing studies have revealed many novel macrophage subpopulations with distinct marker profiles, activation states, and functional programs demonstrating roles in injury, repair, fibrosis, infection, or antigen presentation depending on their specific context (Lin et al, 2019; Wei et al, 2021; Chen et al, 2022; Krasniewski et al, 2022; Yao et al, 2022; Decano et al, 2023; Li et al, 2023; Yu et al, 2024b). These new subpopulations reshape our understanding of polarization beyond the M1/M2 polarization paradigm.

Here, we sought to use single-cell RNA-seq to explore the diversity of the innate immune response and to understand its potential to be reprogrammed by antimicrobial therapy. Antimicrobials primarily target specific aspects of the pathogen’s lifecycle to inhibit its spread, yet few drugs can effectively control viruses, bacteria, and other pathogens, including fungi, while improving cellular health. The only possible way to achieve this is to alter the host immune repertoire to effectively inhibit pathogenic survival. We chose to study the antimicrobial compound K21, a quaternary ammonium silane compound that not only exhibits broad-spectrum effects against viruses, bacteria, and fungi but also demonstrates potent wound healing activity (Gulve et al, 2016; Tweedy et al, 2017; John et al, 2019; Daood et al, 2021, 2025; Bapat et al, 2023). As macrophages and macrophage polarization orchestrate the phases of wound-healing, shifting from pro-inflammatory signaling that combats infection to pro-regenerative signaling that recruits fibroblasts and keratinocytes to repair the epithelial tissue, we reasoned that K21 might operate by modulating macrophage properties, classes, and/or dynamics (Koh & DiPietro, 2011; Al Sadoun, 2022). We chose to examine the effects of K21 on monocyte-derived macrophages from human peripheral blood monocytes (PBMCs) because of their technical accessibility, their ability to be isolated in a relatively naïve state and co-cultured with other cell types, and their controllable M1/M2 polarization (Palmer et al, 2025). We found that supplementing cytokine and cell-surface marker profiling with scRNA-seq transcriptomic analysis provides unparalleled resolution of macrophage diversity and plasticity, allowing us to demonstrate how treatment with K21 remodels the macrophage repertoire both inside and outside the cell. We show that a key aspect of K21 treatment is the induction of mitochondrial fission and autophagy (mitophagy). We turned to the distantly related model system *C. elegans* to examine whether the mechanism used by K21 is used systemically in vivo and evolutionarily conserved. K21 induced DRP-1–mediated mitochondrial fission and mitophagy in vivo without impairing viability, development, or fertility; indeed, it reprogrammed metabolic gene expression and extended lifespan in these nematodes, consistent with its effects observed in mammalian cells. Taken together, our results reveal how metabolic changes in the innate immune system can be leveraged by a potent antimicrobial and sanative agent.

## Results

### Monitoring HMDMs from PBMCs

**Figure 1.**
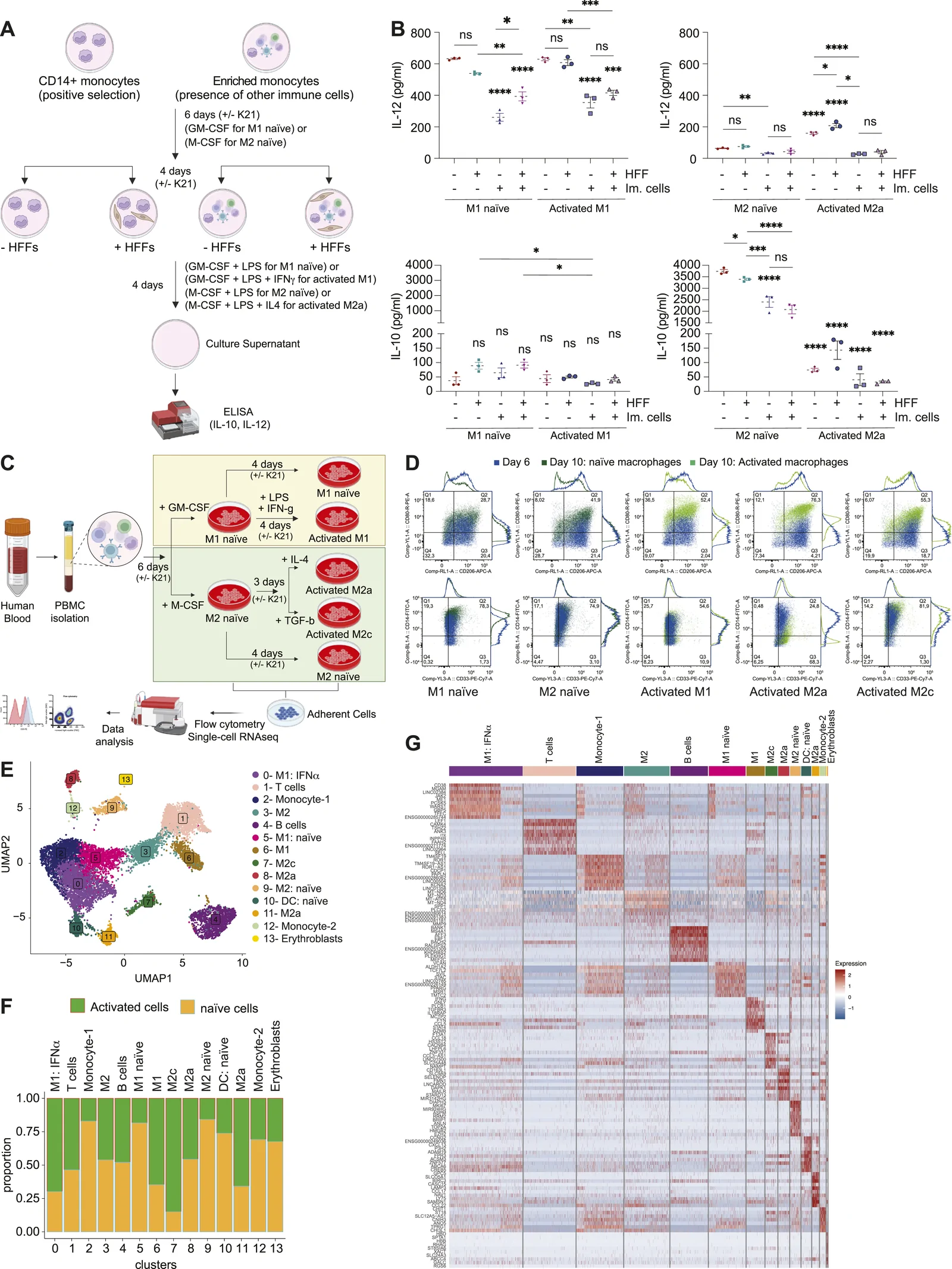
Monitoring human monocyte–derived macrophages from PBMCs. **(A)** Schematic of the experimental setup to study the cytokine secretion profiles (IL-10 and IL-12) of macrophages by ELISA. **(B)** Scatter plots for secreted IL-12 and IL-10 amounts from macrophages from three independent experiments (n = 3). IL-12 values: *P < 0.05, **P < 0.01, ***P < 0.001, and ****P < 0.0001. HFF, human foreskin fibroblast. Two-way ANOVA/Bonferroni post hoc test. ns, not significant. **(C)** Schematics of the experimental setup to study cell-surface protein expression and gene expression profiles of macrophages at the single-cell level. **(D)** Dot plots for CD80 versus CD206 and CD14 versus CD33 cell-surface expression on macrophages in the presence of an immune cell repertoire. Representative image of one replicate experiment. **(E)** UMAP plots of 14 clusters of cells identified from the integrated single-cell RNA-seq analysis. Clusters are numbered, and the respective cell types are indicated. **(F)** A barplot showing the proportion of cells between naïve and activated macrophages. **(G)** Heat map showing differential gene expression from integrated scRNA-seq analysis across scRNA-seq data (without K21 treatment) from naïve and activated macrophages, which were integrated and clustered into 14 populations. The top 10 differentially expressed genes are shown for each cluster. Wilcoxon rank-sum test. DC, dendritic cells; NK, natural killer cells. Source data are available for this figure.

We used an in vitro model of primary HMDMs to test the hypothesis that the antimicrobial K21 modulates innate immunity and macrophage function. We reasoned that human PBMCs would provide an effective system for studying macrophage plasticity and the ability of known macrophage modulators to alter the host immune repertoire. As a first step, we characterized various macrophage populations isolated from human blood that had not yet been exposed to K21. As the presence of immune and/or stromal cells can influence innate immunity and macrophage differentiation, we examined macrophages derived from either enriched monocytes (including other immune cell types) or positively selected CD14^+^ monocytes, which were co-cultured in the presence or absence of human foreskin fibroblasts (HFFs) before initial polarization and activation (Fig 1A). Using ELISA, we analyzed macrophage cytokine secretion profiles (IL-10 and IL-12; Fig 1B) and found that IL-10 and IL-12 secretion patterns clearly distinguished M1 (pro-inflammatory, high IL-12 secretion) macrophages from M2 (anti-inflammatory, high IL-10 secretion) macrophages. Cytokine secretion profiles from total monocytes (i.e., presence of other immune cells) and isolated CD14^+^ monocytes were both as expected, with the presence of other immune cells and/or stromal cells (HFFs) showing a mild depression in the levels of secreted IL-10 and IL-12, similar to previous observations (Vancheri et al, 2001; Ozdemir et al, 2019; Buechler et al, 2021; Mohammad-Hasani et al, 2026).

To look at changes in gene expression at the single-cell level, as well as changes in cell-surface marker expression, we repeated our analysis (Fig 1C), focusing on conditions in which immune cells (enriched monocytes) were present, but without supplementation with stromal cells, which had only moderate effects on cytokine secretion. Initial cytokine treatment was used to promote monocyte-to-macrophage differentiation (6 d of GM-CSF to promote M0 GM-CSF–derived HMDMs henceforth termed naïve M1 cells for simplicity, or M-CSF to promote M0 M-CSF–derived HMDMs henceforth termed naïve M2 cells for simplicity), followed by 3–4 d of either no additional cytokines (to maintain naïve M1 and M2), a combination of LPS and IFN-γ (to promote activated M1 cells), IL-4 (to promote activated M2a cells), or TGF-β (to promote activated M2c cells). Adherent cells were then analyzed by flow cytometry for key cell-surface markers (CD14, CD33, CD80, CD206, and CD163). Similar to our analysis of secreted cytokines, cell- surface markers from human PBMCs showed, as expected, elevated CD80 in activated M1 and M2a cells, and elevated CD206 in activated M2a and M2c (representative images in Fig 1D). By contrast, only modest changes in pan-macrophage markers CD14 and CD33 were observed. Taken together, our results show that monocyte-derived macrophages from human PBMCs display the typical polarization classes, as defined by standard cytokine secretion and cell-surface marker expression, including the modulation typically observed when other immune or stromal cells are present.

To gain a more dynamic and detailed view of macrophage functional diversity beyond simple polarization, we performed single-cell RNA sequencing (scRNA-seq) on the same cells analyzed by flow cytometry. Single-cell transcriptomics elegantly separated M1 and M2 macrophages (both naïve and activated) into distinct population clusters based on their individual gene expression profiles (Fig 1E). To examine the effects of activation on gene expression, we measured the proportion of naïve versus activated cells in each cluster (Fig 1F). The monocyte-to-macrophage ratio and the state of cellular differentiation under naïve and activated conditions clearly captured the differences in gene expression between these two macrophage populations. We found that activation drove more total monocytes into clusters characterized as M1/IFNα, M2c, and M2a, whereas clusters characterized as unpolarized monocytes, naïve M1 and M2 cells, naïve dendritic cells, and erythroblasts were depleted from naïve-treated cells. Each cluster could be identified by a specific set of enriched genes (Fig 1G). These results show that specific subpopulations of macrophages are induced by cytokine activation of PBMCs, and that these populations can be identified by their single-cell transcriptional signatures, corroborating existing knowledge about macrophages and suggesting that each macrophage type carries its signature both inside and outside the cell.

### K21 alters selective properties of macrophages without interfering with monocyte–macrophage differentiation

**Figure 2.**
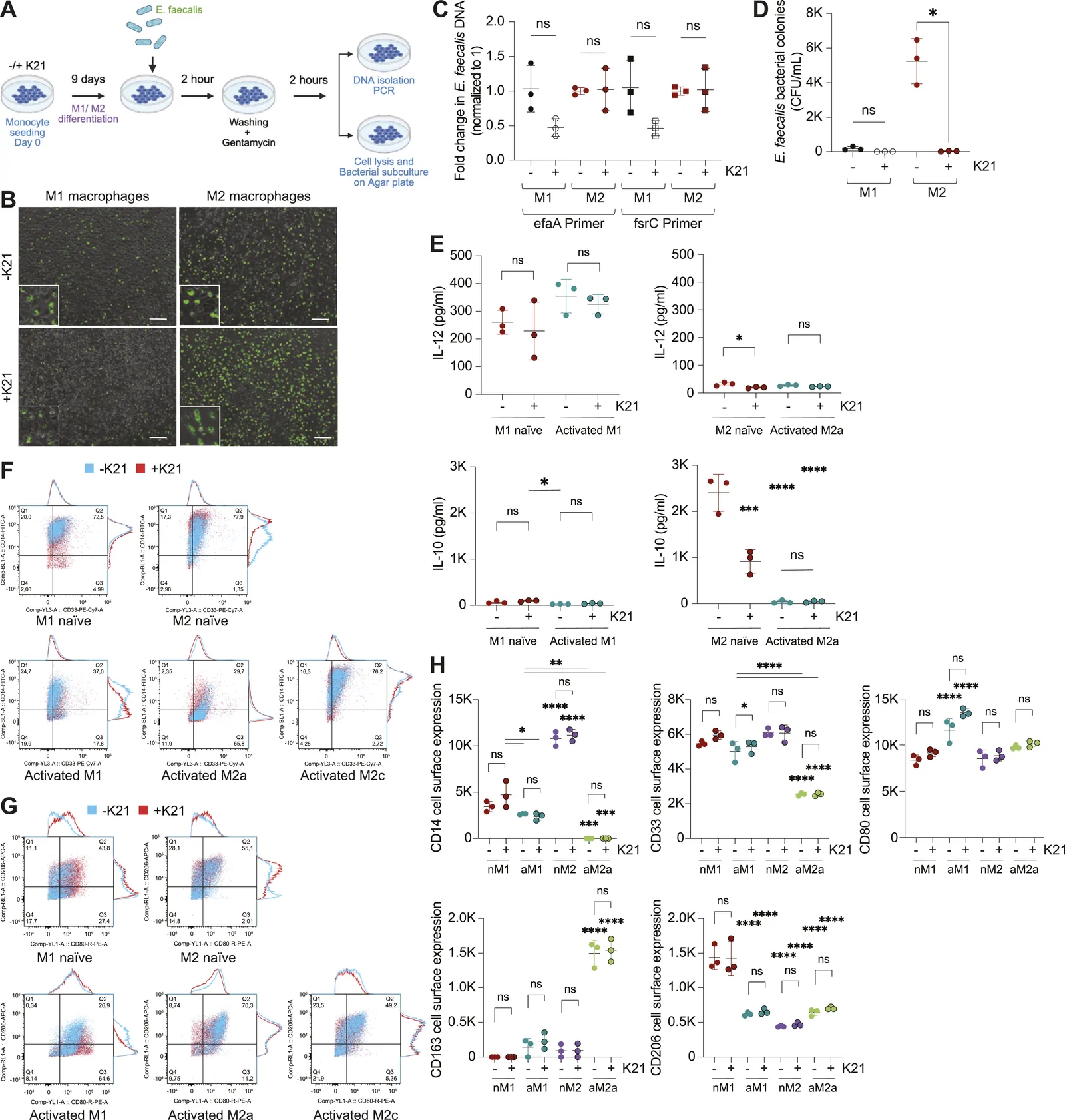
K21 promotes macrophage clearing of infection with only modest effects on cytokine secretion and cell-surface protein expression. **(A)** Schematic of the experimental setup to study the ability of K21-treated macrophages to clear *E. faecalis* infection. **(B)** Fluorescence micrographs of macrophages infected with *E. faecalis* expressing GFP to show uptake of bacteria by phagocytosis. Bar, 10 microns. High-resolution insets of a portion of the image are shown for each image. **(C)** Scatter plots showing copies of the *E. faecalis* genome detected by qPCR in M1 or M2 populations using two different primer sets in three independent experiments (n = 3). **(D)** Number of bacterial colonies isolated from macrophages after infection in three independent experiments (n = 3). Paired *t* test, *P = 0.02. **(E)** Scatter plots for secreted IL-12 and IL-10 amounts from macrophages grown in the presence of other immune cells from three independent experiments (n = 3). IL-12 values: M2 naïve −K21 versus +K21, *P = 0.02. IL-10 values: M1 naïve −K21 versus M1a −K21, *P = 0.03; M2 naïve −K21 versus +K21, ***P = 0.0009; M2 naïve −K21 versus M2a −K21, ****P <0.0001; M2 naïve −K21 versus M2a +K21, ****P < 0.0001. Two-way ANOVA/Bonferroni post hoc test. ns, not significant. **(F, G)** Dot plots for (F) CD14 versus CD33 and (G) CD80 versus CD206 cell-surface expression on macrophages grown in the presence of the immune cell repertoire. Representative image of one replicate experiment. **(H)** Scatter plots for median fluorescent intensity (MFI) of cell-surface markers (CD14, CD33, CD80, CD163, and CD206) from either naïve M1 (nM1), activated M1 (aM1), naïve M2 (nM2), or activated M2a (aM2a) polarized macrophages from three independent experiments (n = 3). CD14 values: nM1 versus nM2 −/+K21, ****P < 0.0001; nM1 versus aM2a −/+, ***P = 0.0003; nM1 +K21 versus aM1 −/+K21, aM1 versus aM2a −/+K21 **P = 0.005; *P < 0.05. K21. CD33 values: nM1 −K21 versus aM2a −/+K21, ****P < 0.0001; M1a −K21 versus +K21, *P = 0.03; aM1 −K21 versus aM2a −/+K21, ****P < 0.0001. CD80 values: nM1 −K21 versus aM1 −/+K21, ****P < 0.0001. CD163 values: nM1 −K21 versus aM2a −/+K21, ****P < 0.0001. CD206 values: nM1 −K21 versus aM1 −/+K21, ****P < 0.0001; nM1 −K21 versus nM2 −/+K21, ****P < 0.0001; nM1 −K21 versus aM2a −/+K21, ****P < 0.0001. Two-way ANOVA/Bonferroni post hoc test. ns, not significant. Source data are available for this figure.

Given previous observations of the antimicrobial and wound-healing properties of the agent K21, which is nontoxic to human PBMCs at micromolar concentrations (Gulve et al, 2016), we reasoned that K21 treatment would alter the immune repertoire of PBMCs in our system. To explore this idea, we monitored the ability of K21 treatment of PBMCs to facilitate the clearing of an *E. faecalis* infection. We chose *E. faecalis* based on its clinical relevance, measurable immune-evasion traits (e.g., its ability to survive and persist within macrophages), and well-validated genetic and quantitative macrophage assays (Archambaud et al, 2024). Monocyte-to-macrophage differentiation was induced (9 d of GM-CSF to promote M1 polarization, or M-CSF to promote M2 polarization) in either the presence or absence of K21 in the culture media (Fig 2A). After 9 d, the media was replaced with *E. faecalis* in K21-free media for 2 h. Any bacteria not taken up by cells were removed by washing with fresh medium and gentamicin, and the cultures were assayed at 2 h using qPCR to detect *E. faecalis* genome copies. In general, the *E. faecalis* infection burden was higher in M2 macrophages compared with M1 as detected by fluorescence microscopy using a strain of GFP-expressing *E. faecalis* (Fig 2B), consistent with the general anti-microbial properties of M1 macrophages. Bacterial DNA copy number analysis revealed no major difference in bacterial entry in the presence of K21 (Fig 2C). However, when the cells were lysed and the bacteria released from them were subcultured, almost no colonies formed from M1 macrophages regardless of treatment, and few colonies formed from M2 macrophages pretreated with K21 compared with untreated M2 controls (Fig 2D), indicating that K21 could reprogram M2 macrophages to interfere with bacterial infectivity.

The ability of K21 treatment to clear macrophages of bacterial infection, combined with previously published observations of its antimicrobial and wound-healing properties, suggested that this agent might modulate immunity. We tested this idea by characterizing the effect of K21 treatment on cytokine secretion under the same culture conditions used for macrophage polarization and activation (Fig 1A), with or without K21. K21 had negligible effects on both IL-12 and IL-10 secretion, except for naïve M2 macrophages, which were suppressed for IL-10 secretion (Fig 2E), suggesting that K21 slightly dampens the anti-inflammatory nature of M2 cells. We also examined cell-surface marker expression by flow cytometry (Fig 1C). K21 had a limited effect on cell-surface protein expression (CD14, CD33, CD80, CD163, and CD206) in both M1 and M2 macrophages, and it did not show any significant effect on the cell-surface expression of CD14 and CD33 (Fig 2F), or on the polarization markers CD80, CD163, and CD206 (Fig 2G and H). These results suggest that K21 does not switch macrophages between the M1 and M2 phenotypes.

### K21 rewires macrophage function and behavior at the transcriptional level

**Figure 3.**
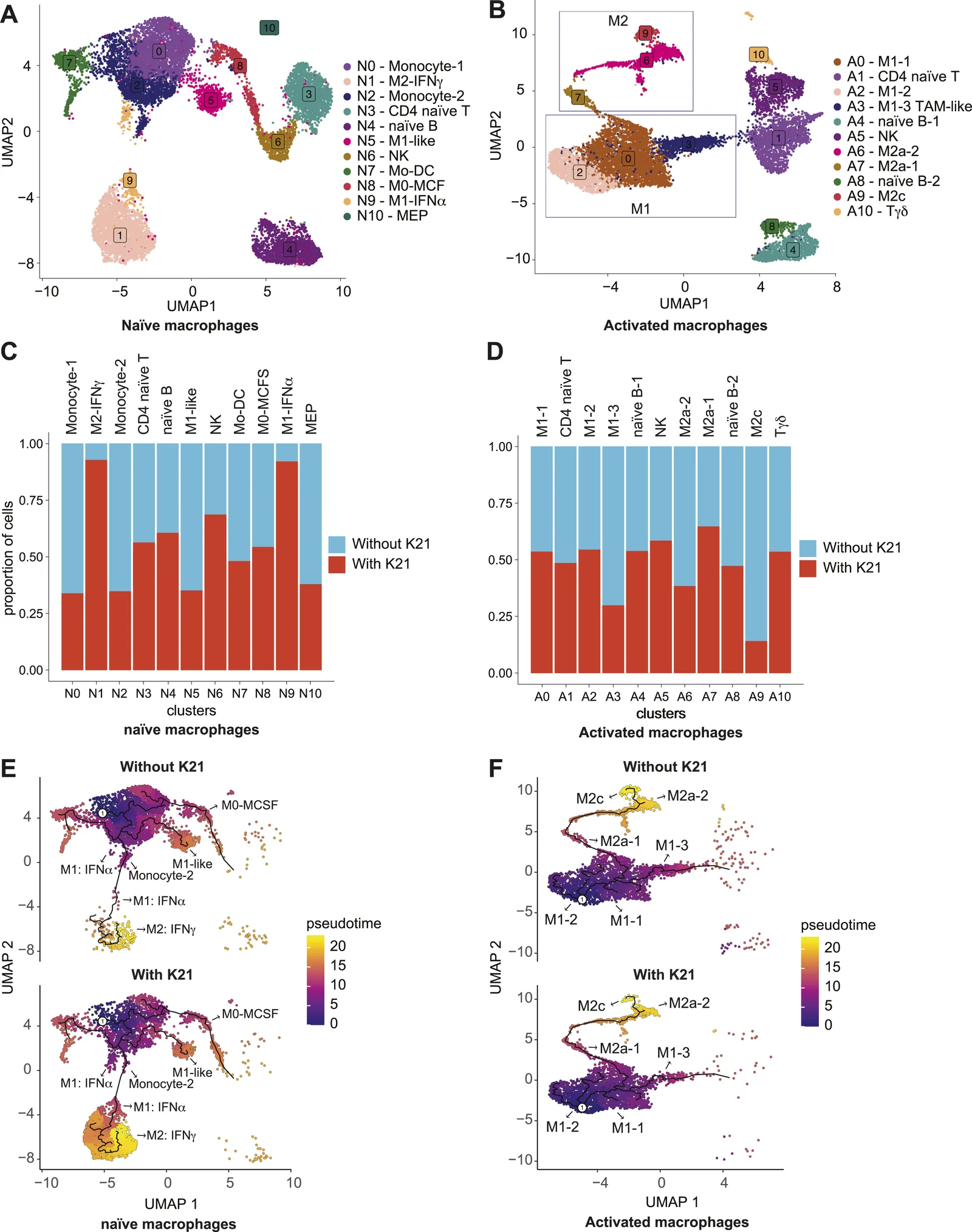
K21 rewires macrophage function and behavior at the transcriptional level. **(A)** UMAP plots of 11 clusters of cells identified from single-cell RNA-seq analysis of K21-treated and untreated naïve macrophages. Cluster-specific cell types are indicated. **(B)** Similar UMAP analysis of activated macrophages. **(C, D)** A barplot showing changes in the proportion of various cell types from (C) naïve or (D) activated populations in the presence (red) and absence (blue) of K21. The distribution of K21-treated versus untreated cells into clusters N0-N6, N9, A0, A2, A3, A5–A7, and A9 was unlikely to be by chance (*P* < 0.05) as determined by binomial analysis adjusted for multiple comparisons using a Bonferroni correction. **(E, F)** UMAP visualization of trajectory and pseudotime computed from macrophage scRNA-seq data from (E) naïve or (F) activated populations (K21-treated on bottom, untreated on top). Cells are colored by pseudotime. Only monocyte-derived cell populations are included within the images. Source data are available for this figure.

**Figure S1.**
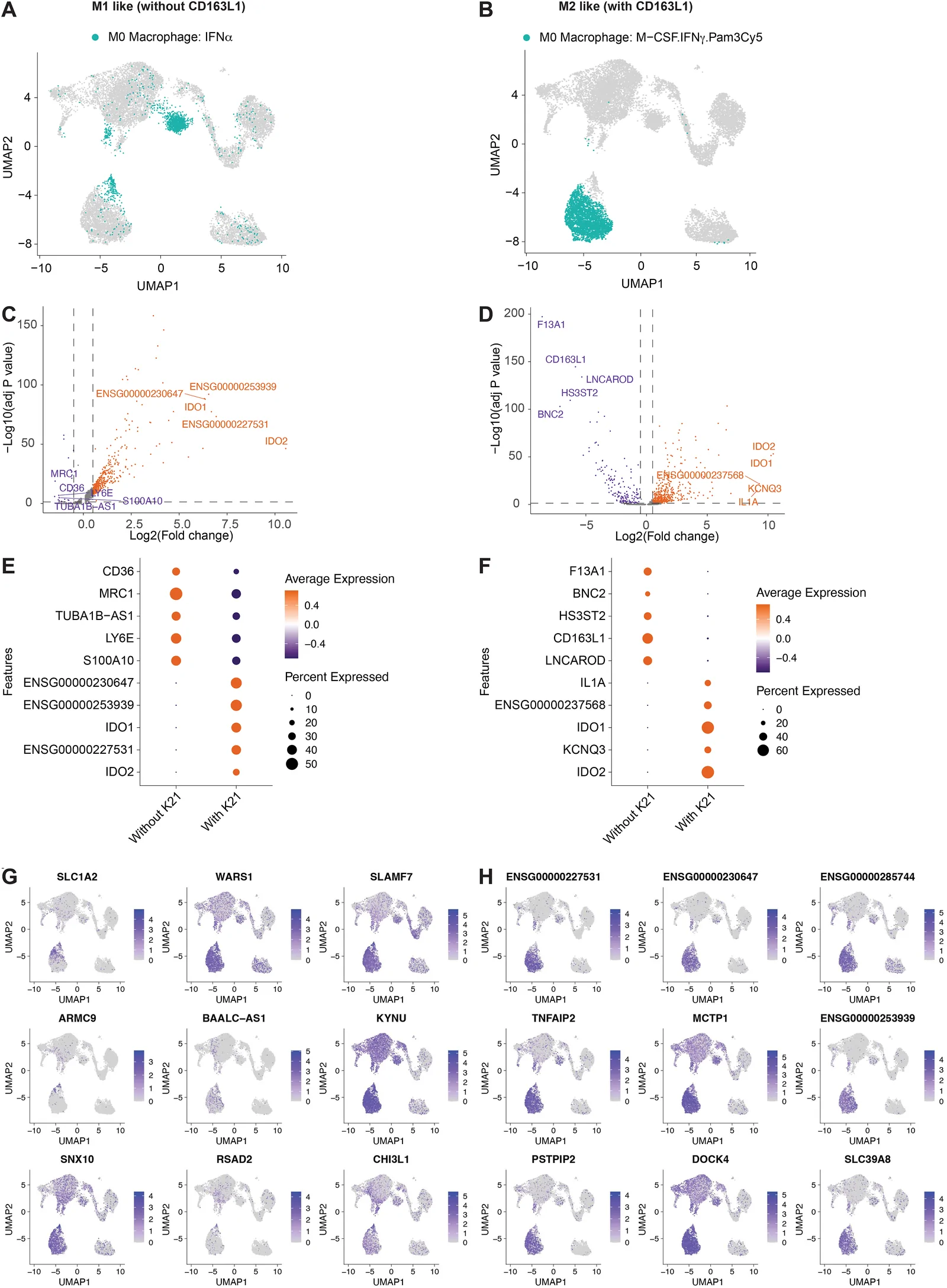
K21-induced transcriptional remodeling of naïve macrophage subpopulations. **(A)** UMAP plots of cluster N9 identified from the integrated single-cell RNA-seq analysis of naïve macrophages. **(B)** UMAP plots of cluster N1 identified from the integrated single-cell RNA-seq analysis of naïve macrophages. **(C, D)** Volcano plots of log_2_ fold changes and log_10_ adjusted *P*-values for individual genes detected by scRNA-seq in cluster N9 (C) and cluster N1 (D) grown in the presence of K21. Top candidate genes up-regulated (orange) or down-regulated (purple) with logFC >0.5 and adjusted *P*-value <0.05 are indicated. **(E, F)** Dot plots showing average differential expression of the top 5 up-regulated or down-regulated genes within cluster N9 (E) and cluster N1 (F) are shown. **(G, H)** UMAP projections colored by gene expression of the top 9 genes within cluster N9 representing M1-IFNα-2 cell types (G) and cluster N1 representing M2-IFNγ cell types (H), which were enriched in the presence of K21 in naïve macrophages.

K21 did not induce complete class-switching behavior of macrophages, but instead induced subtle changes in cytokine secretion and antibacterial properties, suggesting that K21 might be augmenting macrophage function at a level beyond the standard macrophage polarization markers. We reasoned that the finer details of these functional changes would be reflected at the level of gene expression and the transcriptome. We, therefore, carried out single-cell transcriptomics of the immune cell repertoire after 10 d of differentiation, pooling all cells from our culture analysis (Fig 1C) into four separate libraries based on two variables: (1) naïve versus activated (with M1 and M2 macrophage populations combined) and (2) the presence or absence of K21. The four separate pools contained comparable numbers of unique transcripts and mitochondrial RNA species, and UMAP cell clustering comparisons between naïve and activated macrophages, each including both the K21-treated and untreated pools, effectively captured distinct immune cell types in each of the two clustered populations (Fig 3A and B). Clusters were assigned putative identities based on the presence of established markers for known monocyte and immune cell types. Cell type distribution analysis within each clustered population in either the naïve or activated macrophage pools was then categorized by K21 treatment to determine whether any clusters showed enrichment (Fig 3C and D). Two distinct naïve macrophage populations (clusters N1 and N9) were enriched in the presence of K21 (Fig 3C). The smaller macrophage population (N9) lacked CD163L1 expression and was, therefore, identified as M1 naïve macrophages; this population exhibited an IFN-α-stimulated macrophage profile (Fig S1A). The largest K21-induced naïve macrophage population (N1) expressed CD163L1 and was, therefore, identified as M2 naïve macrophages; this population exhibited an IFN-γ–stimulated macrophage profile (Fig S1B). Pseudotime analysis revealed that the M1-IFN-α and M2-IFN-γ populations (clusters N9 and N1, respectively) likely represented terminal differentiation states from naïve M0 monocytes (Fig 3E).

By contrast, another distinct cluster of M1 naïve macrophages with the characteristics of IFNα−stimulated macrophages was underrepresented in K21-treated cells (cluster N5). Activated M1 macrophages with the same gene expression profile were also underrepresented in K21-treated cells (cluster A3). In addition, the number of classical M2c macrophages significantly decreased in the presence of K21 (cluster A9). Pseudotime analysis revealed that the three M2 populations (clusters A6, A7, and A9) from the activated macrophage populations likely represented terminal differentiation states (Fig 3F).

Taken together, our UMAP and pseudotime analyses indicate that K21 promoted the stepwise differentiation of naïve M0 monocytes towards M1-IFN-α and M2-IFN-γ macrophage populations (clusters N9 and N1), whereas it reduced the differentiation of activated M2c macrophages (cluster A9). Thus, scRNA-seq can reveal populations of macrophages otherwise hidden by traditional cytokine/surface marker analyses, and an immunomodulatory agent such as K21 can drive macrophage differentiation towards or away from specific populations.

### K21 promotes the expression of genes associated with immune activation

**Figure 4.**
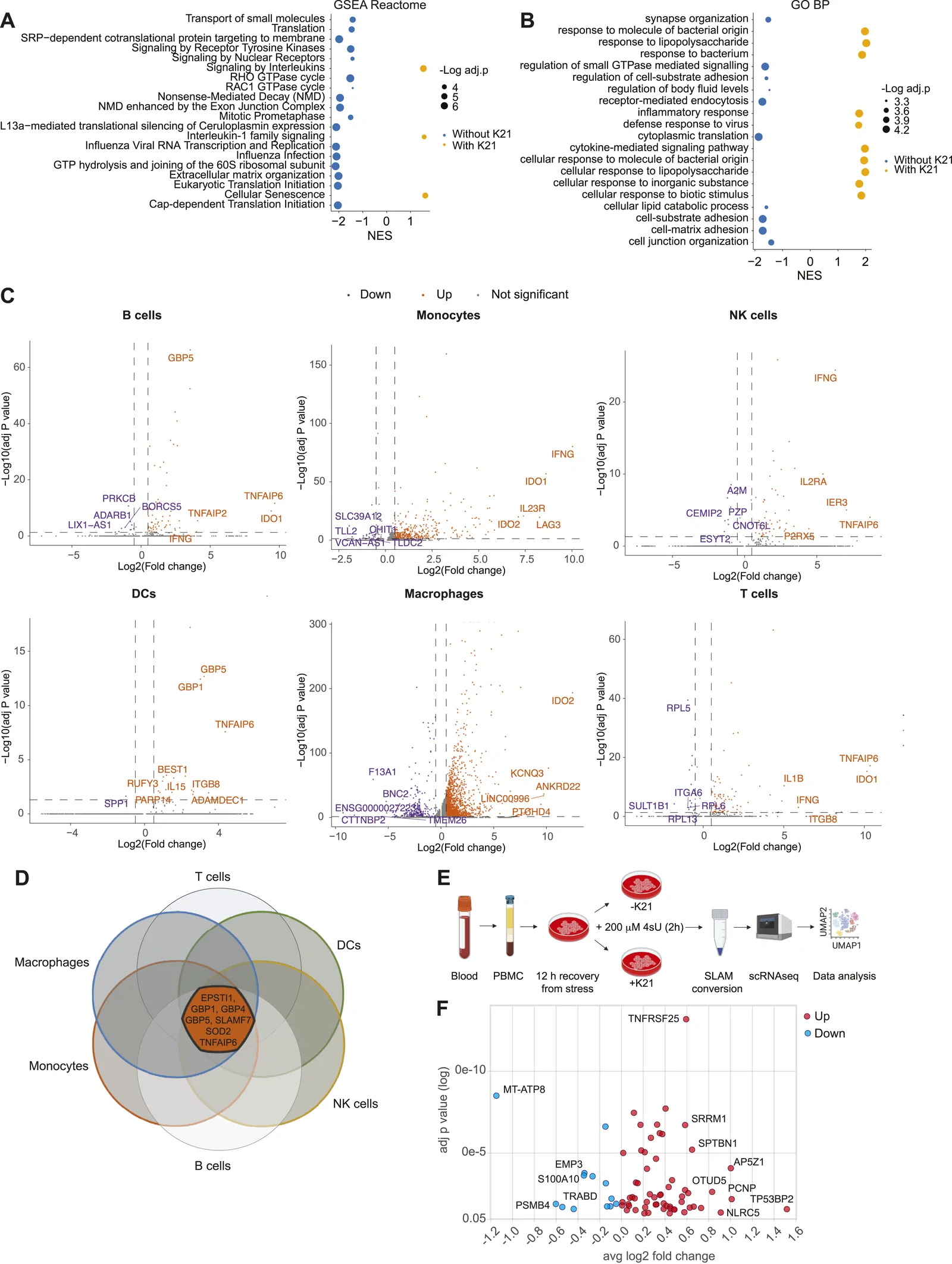
K21 promotes the expression of genes associated with immune activation. **(A, B)** Bubble plots showing significantly enriched pathways from (A) the pairwise GSEA Reactome analysis or (B) Gene Ontology Biological Pathway (GO-BP) term analysis of the two pro-inflammatory macrophage populations enriched within naïve macrophages. The significantly enriched pathways are visualized for cells with and without K21. The size of each bubble is proportional to the number of core genes within the pathway. NES, normalized enrichment score. **(C)** Volcano plots of log_2_ fold changes and log_10_ adjusted *P*-values for individual genes detected by scRNA-seq in various cell types grown in the presence of K21. Top candidate genes up-regulated (orange) or down-regulated (purple) with logFC more than 0.5 and adjusted *P*-value less than 0.05 are indicated. **(D)** A Venn diagram showing the common genes up-regulated by K21 in all the identified cell types. **(E)** Schematics of the experimental setup to study dynamic changes in RNA expression within the first two h of K21 exposure in peripheral blood mononuclear cells (PBMCs). **(F)** Bubble plot of log_2_ fold changes and log_10_ adjusted *P*-values for individual newly synthesized genes with log2FC more than 0.5 and adjusted *P*-value less than 0.05 as detected by scRNA-seq in the presence of K21. Top candidate genes up-regulated (red) or down-regulated (blue) are indicated.

K21 had a more dramatic impact on population clusters in naïve macrophages (Fig 3C) than it did in activated macrophages (Fig 3D); therefore, we focused subsequent analysis on naïve macrophages. Specifically, K21 treatment expanded the naïve pro-inflammatory macrophages found in the M1-IFN-α and M2-IFN-γ clusters (N9 and N1). To better understand the transcriptional basis of these K21-induced cell clusters, we pooled gene expression data from these two populations and analyzed Reactome gene set enrichment analysis (GSEA) and Gene Ontology (GO) term enrichment. Reactome GSEA revealed uniform enrichment for Interleukin signaling, IL-1 signaling, and cellular senescence in the presence of K21 (Fig 4A). GO biological processes analysis revealed enhanced responses in macrophages to LPS, bacteria, viral infections, and other inorganic and biotic stimuli in the presence of K21 (Fig 4B). Interestingly, both these macrophage populations showed similar yet distinct pro-inflammatory gene expression profiles (Fig S1C and D) with enhanced expression of IDO1, IDO2, and KYNU (Fig S1E–H). These systems biology approaches suggested that K21 induces distinct pro-inflammatory macrophage populations, including M1 and M2 macrophages, enhancing their ability to fight infections. In addition, the induction of specific genes in the presence of K21 suggests metabolic reprogramming that makes macrophages more powerful in preventing various types of infections.

We also examined whether K21 treatment might affect gene expression across multiple cell types, perhaps reflecting a common cellular mechanism. We focused on naïve cells, pooling cluster data into 6 pools: B cells (cluster N4), monocytes (clusters N0, N2, N8), NK cells (cluster N6), monocyte-derived DCs (cluster N7), macrophages (clusters N1, N5, N9), and T cells (cluster N3). K21 treatment up-regulated key genes involved in inflammatory response and kynurenine metabolism, depending on the class of immune cell (Fig 4C). Six genes were significantly up-regulated by K21 across all immune cell types (Fig 4D); no genes were commonly down-regulated across all cell types. Three of the six core genes induced by K21 are implicated in either mitochondrial function or immunity: GTPase-binding protein GBP1, which induces mitochondrial fission and enhances macrophage health (Qiu et al, 2018); SOD2, which improves mitochondrial health through its ROS-scavenging functions (He et al, 2016); and GBP5, which is an interferon-inducible antiviral protein (Krapp et al, 2016).

**Figure S2.**
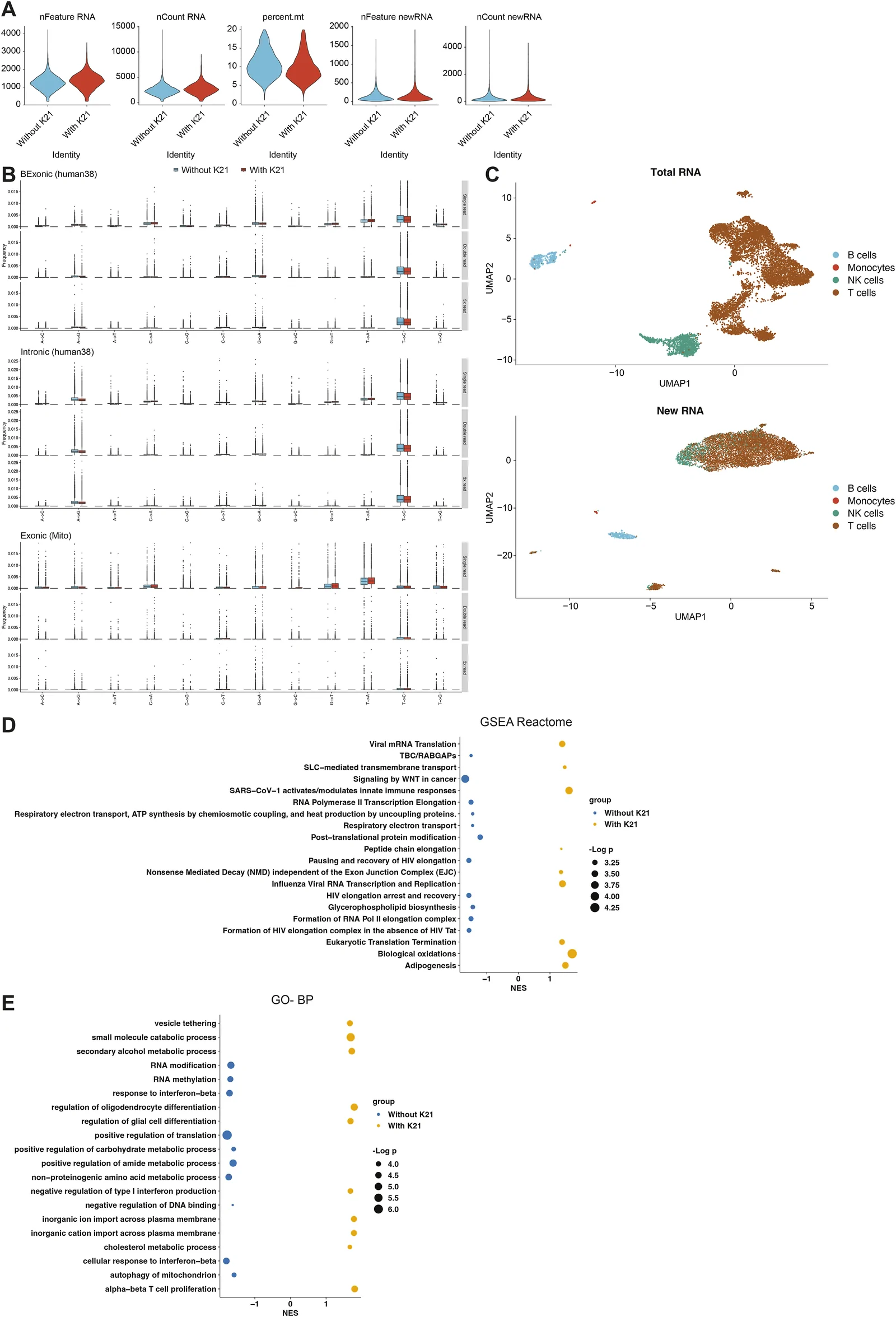
Quality control and pathway analysis of the scSLAM-seq dataset. **(A)** Violin plots showing the quality control of an unbiased scSLAM-seq dataset. The three left plots display the distributions of the number of genes identified in each cell (nFeature RNA), the number of unique molecular identifiers (UMIs) representing transcripts (nCount RNA), and the percentage of mitochondrial transcripts (percent.mt). The two right plots show the distributions of newly synthesized RNA for genes identified in each cell (nFeature RNA) and of UMIs (nCount RNA). **(B)** Box plots showing T to C conversion efficiency within exonic, intronic, and exonic mitochondrial transcripts. **(C)** UMAP plots 4 clusters of cells identified from the integrated single-cell SLAM-seq analysis. The upper panel shows the clustering based on total RNA. Clustering in the lower panel was based on total RNA, but it shows the distribution of clusters for newly synthesized RNA. Clusters are numbered, and the respective cell types are indicated. **(D)** Bubble plot showing significantly enriched pathways from the pairwise GSEA Reactome analysis of the newly synthesized RNA in response to K21. The significantly enriched pathways are visualized for cells with and without K21. **(E)** Bubble plot showing significantly enriched biological processes from the pairwise Gene Ontology (GO)-BP analysis of the newly synthesized RNA in response to K21. The significantly enriched pathways are visualized for cells with and without K21. The size of each bubble is proportional to the number of core genes within the pathway. NES, normalized enrichment score.

Finally, we sought to identify the earliest gene expression changes after K21 treatment, as these might reveal a more direct mechanism. We used a single-cell SLAM-seq approach in human PBMCs to study alterations in gene expression during the first 2 h of K21 exposure (Fig 4E). SLAM-seq pulse-labels newly synthesized RNA within a specific time frame, thereby enabling precise identification of gene transcription rates in response to a particular stimulus (Fig S2A and B). Only T and NK cells were sufficiently metabolically active to capture sufficient 4sU incorporation within 2 h of culture, whereas monocytes and B cells were less in number and did not incorporate enough 4sU within the 2 h of the experiment (Fig S2C) to enable the effects of K21 on these cell types. Both GSEA Reactome and GO-BP analysis (Fig S2D and E) showed altered type I interferon response, biological oxidation in T cells, and enhanced interleukin response in NK cells in the presence of K21. We identified significant suppression of transcription of genes associated with mitochondrial function (Mt-ATP8, TRABD) and calcium signaling (S100A10) in T cells in response to K21 treatment (Fig 4F). The suppression of Mt-ATP8 was relevant, as our scRNA-seq data also identified specific macrophage clusters (N5 and A3) enriched for Mt-ATP8, which were depleted in the presence of K21. Taken together, our results suggest that K21 possibly affects mitochondrial function in all treated cells.

### K21 induces mitophagy and remodels mitochondrial metabolic gene expression

**Figure 5.**
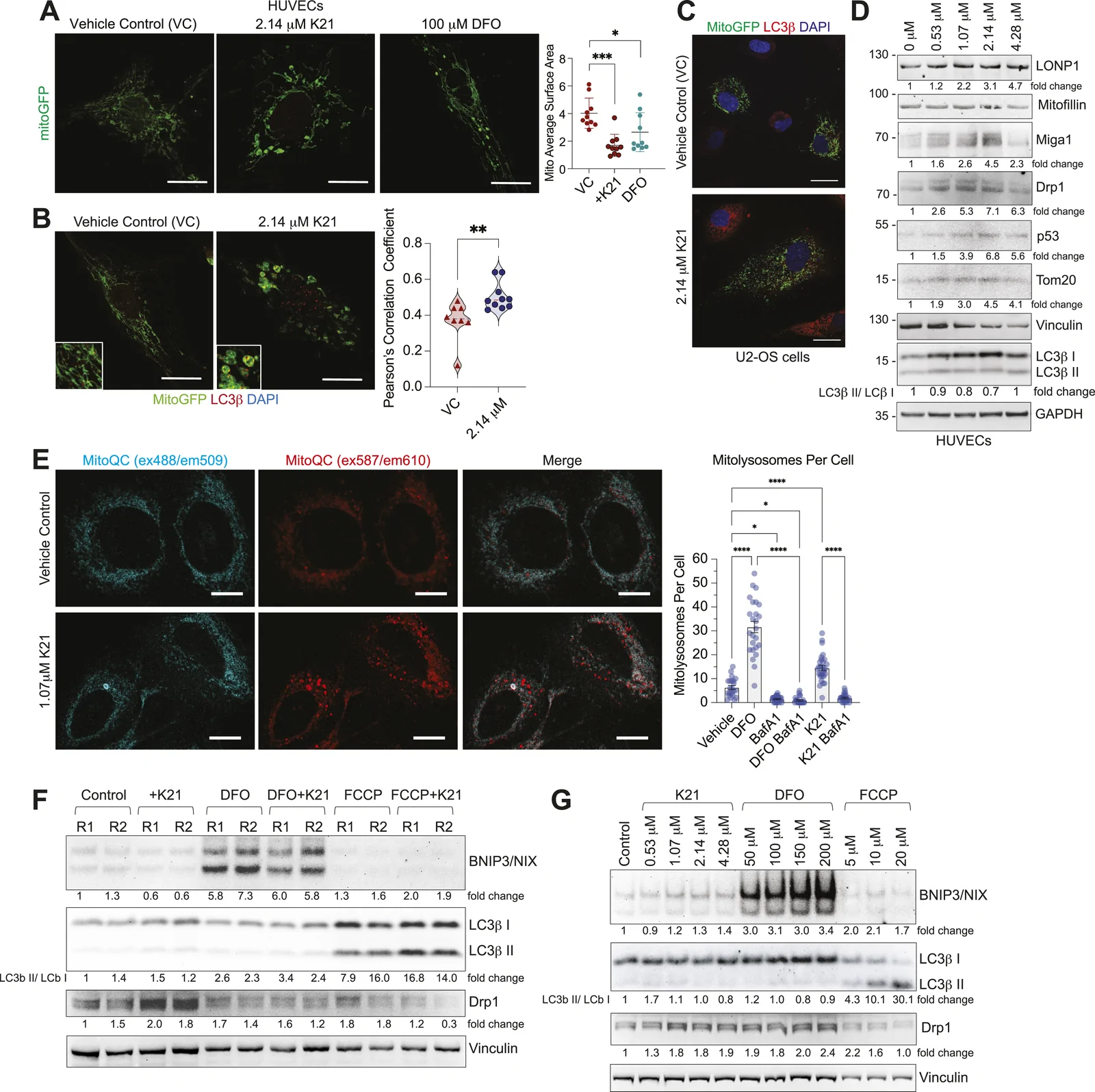
K21 induces mitophagy and remodels mitochondrial metabolism. **(A)** Visualization using confocal imaging of K21-induced or DFO-induced mitochondrial fragmentation in HUVEC cells expressing mitoGFP. Average mitochondrial surface area was quantified from at least five independent images per condition and is plotted as a box-and-whiskers plot. *P = 0.03, ***P = 0.0003, ANOVA/Bonferroni post hoc test. **(B)** Confocal images of HUVEC cells showing colocalization of LC3β to mitochondrial fission compartments labeled with MitoGFP. Violin plot showing Pearson’s correlation coefficient from at least 10 independent images (n = 10). **P < 0.005, unpaired Mann–Whitney test. VC, vehicle control. **(C)** Confocal images of U2-OS cells showing colocalization of LC3β to mitochondrial fission compartments labeled with MitoGFP. **(D)** Immunoblots showing concentration-dependent changes in protein expression in the presence of K21 in HUVEC cells. For each detected protein species, the fold change relative to the untreated control is shown below the blot, with all protein levels normalized to their respective Vinculin or GAPDH loading control. **(E)** Confocal images of U2-OS cells expressing MitoQC, allowing visualization of non-mitophagic mitochondria via 488 nm excitation and 509 nm emission, mitolysosomes via 587 nm excitation and 610 nm emission, and a merged image. Bar/dot plot of the number of mitolysosomes per cell showing K21-induced and DFO-induced mitophagy that is blocked by BafA1 treatment. *P < 0.05, ****P < 0.0001, ANOVA/Bonferroni post hoc test. **(F)** Immunoblots showing changes in protein expression in the presence of K21, DFO, and/or FCCP in HUVEC cells. Two independent biological replicates (R1 and R2) are shown side-by-side. For each detected protein species, the fold change relative to the R1 control is shown below the blot, with all protein levels normalized to their Vinculin loading control. **(G)** Immunoblots showing concentration-dependent changes in protein expression in the presence of K21, DFO, or FCCP in U2-OS cells. Quantification as per panel (F). Bar, 10 microns in all images. Source data are available for this figure.

As reprogramming mitochondrial health and metabolism is integral to immune responses (Mills & O’Neill, 2016; Nassef et al, 2021; Kumar et al, 2024), we next asked if K21 directly targets mitochondria. For example, TRABD enhances mitochondrial fusion; thus, we reasoned that a K21-induced decrease in TRABD transcription should facilitate mitochondrial fission. As macrophage mitochondrial markers and morphology can be rather poor, and as transfection into these cells is inefficient and often disrupts their morphology and function, we first chose to examine the effects of K21 on mitochondria in larger cells with mitochondria more amenable to analysis by fluorescence microscopy. We tested the effects of K21 on mitochondrial architecture in primary human umbilical vein endothelial cells (HUVECs) expressing MitoGFP to label individual mitochondria, and we found a marked induction of mitochondrial fragmentation in the presence of K21, as measured by a decrease in average mitochondrial surface area (Fig 5A). Deferoxamine (DFO), a hypoxia mimic commonly used to induce fission and mitophagy in cells, served as a positive control. This result was further validated by LC3β-specific staining in HUVEC cells, where both mitochondria and LC3β colocalized with a significant Pearson’s correlation coefficient (*P* = 0.0025; Fig 5B), indicating an increase in the number of mitochondria undergoing autophagy. We observed similar results in U2-OS cells (Fig 5C). HUVECs showed increased LC3β-I expression in the presence of K21, along with increases in Drp1, Miga1, and LONP1 (Fig 5D), suggesting that K21 induced mitophagy. Our measurements were for total Drp1 protein; we could not measure phosphorylated Drp1 levels at the time of manuscript preparation.

Finally, we examined the effects of K21 treatment on U2-OS cells stably expressing MitoQC, a fluorescent protein comprising GFP, mCherry, and a mitochondrial matrix localization signal (McWilliams et al, 2016). The low-pH environment of mitochondria undergoing mitophagy within lysosomes quenches GFP fluorescence but not mCherry, allowing the mCherry/GFP fluorescence ratio to mark these mitolysosomes and serve as a direct biosensor for mitophagy. Vehicle control U2-OS cells have diverse non-mitophagic mitochondrial networks (observed with MitoQC at 488-nm excitation and 509-nm emission; Fig 5E) and few mitolysosomes (observed with MitoQC at 587-nm excitation and 610-nm emission; Fig 5E). Treatment with K21 increased the number of MitoQC structures observed at 587-nm excitation and 610-nm emission, indicating additional mitolysosomes in these cells (Fig 5E). The observed increase in mitolysosomes resembled that observed with DFO treatment. Both K21-induced and DFO-induced mitophagy were blocked by treatment with Bafilomycin A_1_ (BafA1), a known inhibitor of the lysosomal V-ATPase and blocker of autophagy (Bowman et al, 1988; Wang & Semenza, 1993), strongly supporting a role for K21 in inducing mitophagy.

**Figure S3.**
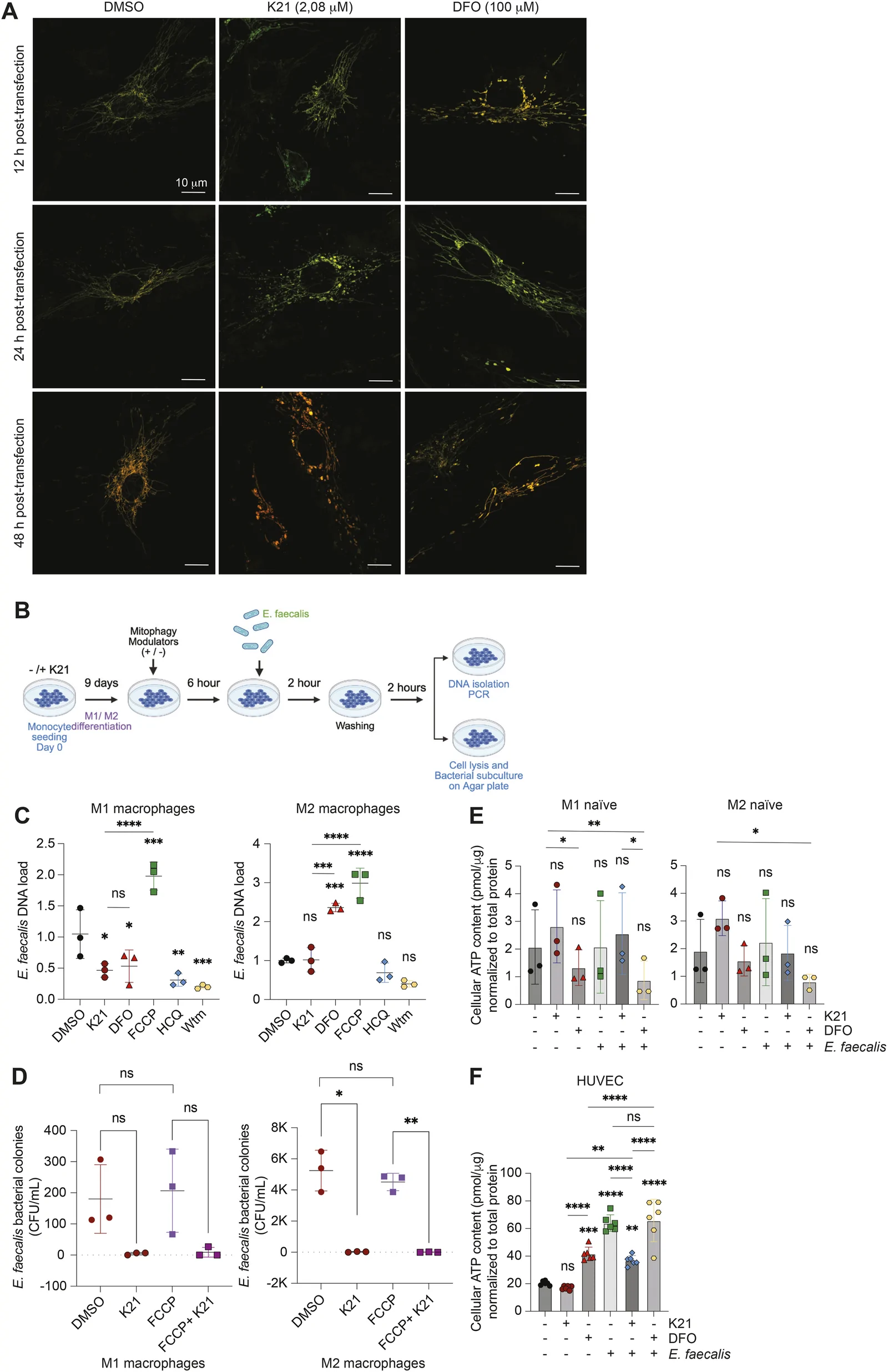
K21 modulates mitochondrial turnover, cellular bioenergetics, and macrophage control of *E. faecalis* infection. **(A)** Dual-channel fluorescence microscopy images of HUVEC cells expressing MitoTimer, with red indicating 543–558 nm excitation and 572–592 nm emission (previously synthesized and oxidized MitoTimer protein), and green indicating 483–488 nm excitation and 500–520 nm emission (newly synthesized MitoTimer protein). Times after transfection are indicated, as are treatments with DMSO (vehicle), K21, or DFO. Bar, 10 microns. **(B)** Schematic of the experimental setup to study the ability of K21-treated macrophages to clear *E. faecalis* infection. **(C)** Scatter plots showing copies of the *E. faecalis* genome detected by qPCR in M1 (left graph) or M2 (right graph) populations in three independent experiments (n = 3). Two-way ANOVA/Bonferroni post hoc test. ns, not significant. DMSO versus K21 (M1), *P = 0.01; DMSO versus DFO (M1), *P = 0.02; DMSO versus FCCP (M1), ***P = 0.0003; DMSO versus DFO (M2), ***P = 0.0002; DMSO versus FCCP (M2), ****P < 0.0001; DMSO versus HCQ (M1), **P = 0.002; DMSO versus Wtm (M1), ***P = 0.0007; K21 versus DFO (M2), ***P = 0.002. **(D)** Number of bacterial colonies isolated from macrophages after infection in M1 (left graph) or M2 (right graph) populations in three independent experiments (n = 3). Paired *t* test. DMSO versus K21, *P = 0.02; FCCP versus FCCP+K21, **P = 0.005. **(E, F)** Cellular ATP measurements in (E, left) M1 naïve macrophages, (E, right) M2 naïve macrophages, or (F) HUVEC cells after the indicated treatment. Paired *t* test. **(E)** Untreated versus K21-treated M1, **P = 0.002. (E) *P < 0.05, **P < 0.01. Two-way ANOVA/Bonferroni post hoc test. ns, not significant. **(F)** Untreated versus DFO, ***P = 0.0005; untreated versus *E. faecalis*, ****P < 0.0001; K21 versus K21+*E.faecalis*, **P = 0.001; *E. faecalis* versus K21+*E.faecalis*, ****P < 0.0001; DFO+*E.faecalis* versus K21+*E.faecalis*, ****P < 0.0001; untreated versus K21+*E.faecalis*, **P = 0.009; DFO versus DFO+*E.faecalis*, ***P = 0.027. Two-way ANOVA/Bonferroni post hoc test. ns, not significant.

To assess mitochondrial turnover, which in turn reflects mitochondrial health, we used MitoTimer proteins in HUVECs (Hernandez et al, 2013). MitoTimer, which comprises a mutant DsRed protein, DsRed1-E5, combined with a mitochondrial matrix localization signal, is a red fluorescent protein targeted to mitochondria whose fluorescence shifts over time from green to red as the protein matures, providing temporal and spatial information on mitochondrial turnover (Hernandez et al, 2013). Accumulation of purely red mitochondria inside the cell indicates a dysfunctional state of mitochondrial biogenesis. For example, DFO treatment results in the accumulation of large, globular mitochondria labeled with a high red/green MitoTimer ratio within 12 h, suggesting the relatively rapid accumulation of dysfunctional mitochondria (Fig S3A). By contrast, K21 treatment induces fission into smaller mitochondrial units with relatively stable red/green MitoTimer signals within 12 h, followed by a slower accumulation of large globular mitochondria 12–36 h later, suggesting that K21 does not induce mitochondrial dysfunction in a manner similar to that of DFO. The presence of both green and red mitochondria in cells undergoing either treatment suggests robust mitochondrial turnover over a 48-h time course.

Mitophagy can be induced in response to mitochondrial dysfunction (e.g., mitochondrial membrane depolarization activating the PINK1/Parkin pathway) or to environmental stress (e.g., hypoxia and HIF1α activating the expression of the mitophagy receptor BNIP3L/NIX) through distinct mechanisms (Clague & Urbe, 2025). To determine whether K21 induces mitophagy via one of these two mechanisms, we treated HUVEC cells and U2-OS cells with either DFO (to induce BNIP3L/NIX–mediated mitophagy), the mitochondrial membrane decoupler FCCP (to induce PINK1/Parkin–mediated mitophagy), or K21, and then examined the levels of key protein mediators by immunoblot (Fig 5F and G). DFO treatment (but not K21 or FCCP treatment) induced BNIP3L/NIX expression and a modest increase in Drp1 fission factor stabilization, as expected for this hypoxia mimetic. By contrast, FCCP treatment (but neither K21 nor DFO treatment) induced the formation of lipid-modified LC3β II, consistent with previous observations that mitochondrial depolarization is a strong inducer of overall autophagy (Lyamzaev et al, 2018). K21 did not induce BNIP3L/NIX expression nor the LC3β II lipidation indicative of gross mitochondrial impairment; rather, it strongly induced the stabilization of Drp1, consistent with the observed increase in mitochondrial fission.

**Figure S4.**
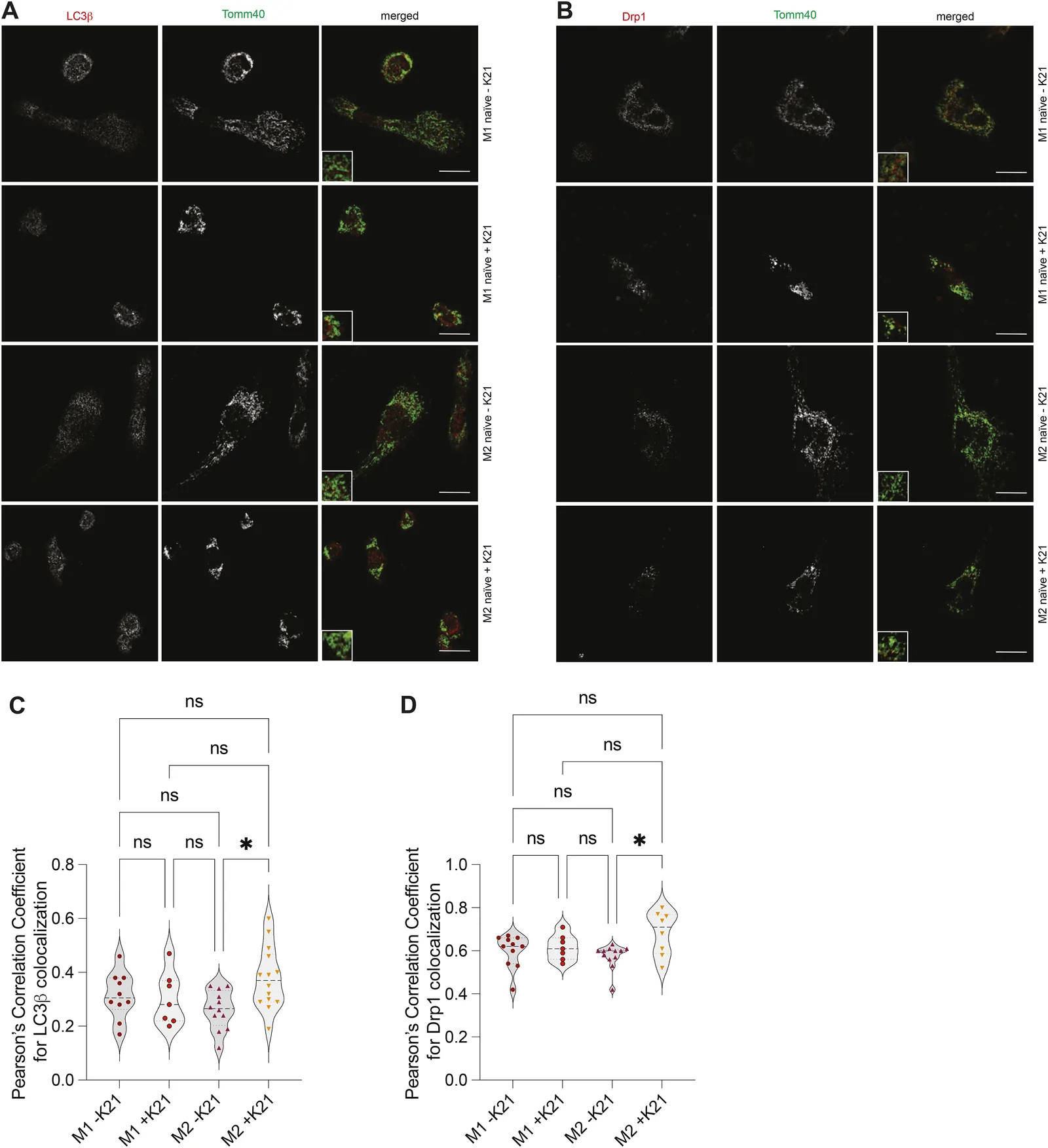
K21 alters mitochondrial association of LC3β and Drp1 in naïve macrophages. **(A, B)** Dual-channel immunofluorescence images of naïve macrophages (either M1 or M2, and either treated with vehicle or K21). High-resolution insets of a portion of the channel-merged image are shown for each row. **(A)** Confocal microscopy images of macrophages showing colocalization of LC3β (red) to mitochondria labeled with Tomm40 (green). **(B)** Confocal microscopy images of macrophages showing colocalization of Drp1 (red) to mitochondria labeled with Tomm40 (green). **(C, D)** Violin plots showing Pearson’s correlation coefficient for either (C) LC3β and Tomm40, or (D) Drp1 and Tomm40 from at least 5 independent images (n = 5). *P < 0.05, one-way ANOVA test. Bar, 10 microns in all images.

Finally, we examined mitochondria directly in our PBMC-derived macrophages. Conventional tools for visualizing mitochondria, which were developed and validated in other cell types around the macroautophagy paradigm, do not translate well to these cells (Li et al, 2020; Lu et al, 2025). Nevertheless, we tested the effects of K21 on mitochondrial architecture in naïve M1 and M2 macrophages using Tomm40-specific immunofluorescence to monitor mitochondria, combined with either LC3β-specific staining to monitor mitophagy (Fig S4A) or Drp1-specific staining to monitor mitochondrial fission (Fig S4B). Mitochondria in both types of untreated macrophages were smaller and had a more fragmented morphology compared with those observed in HUVEC and U2-OS cells, consistent with previous observations (Li et al, 2020; Lu et al, 2025). K21 treatment resulted in coalesced mitochondria with a more globular morphology compared with untreated macrophages. K21 also triggered an increase in Tomm40 colocalization with both LC3β and Drp1 in naïve M2 macrophages (Fig S4C and D), suggesting a possible increase in fission and mitophagy in these cells, consistent with our observations in HUVEC and U2-OS cells. Taken together, these observations corroborated scRNA-seq data from macrophages, suggesting that K21-mediated fission and mitophagy operate distinctly from previously described mitophagy pathways and likely enhance mitochondrial health, metabolism, and quality control.

### Mitophagy enhances bacterial phagocytosis but not infectivity

The ability of K21 to induce mitophagy prompted us to check if mitophagy can directly alter bacterial growth in macrophages. We tested the phagocytosis abilities of HMDMs against *E. faecalis* in both M1 and M2 macrophages. The macrophages were pretreated with either DFO or FCCP as positive inducers of mitophagy, or with wortmannin or hydroxychloroquine as negative regulators of mitophagy, for 6 h before bacterial infection. K21 treatment was initiated at the start of monocyte-to-macrophage differentiation (Fig S3B). The mitophagy inducer FCCP significantly enhanced bacterial entry into both M1 and M2 macrophages, whereas DFO increased bacterial phagocytosis only in M2 macrophages (Fig S3C). As expected, wortmannin significantly decreased bacterial phagocytosis only in M1 macrophages (Fig S3C). Even though FCCP significantly enhanced bacterial phagocytosis, bacterial infectivity was not proportionately increased (Fig S3D), suggesting that mitophagy enables better phagocytosis of bacteria but does not allow them to remain infective. K21’s negative effect on bacterial growth was maintained even in the presence of FCCP-mediated increased bacterial entry. These results suggest that K21 and other mitophagy inducers enhance bacterial phagocytosis, whereas K21 is extremely effective in suppressing bacterial infectivity.

*E. faecalis* does not require a cellular ATP source for its survival (Keogh et al, 2018); however, host cell ATP plays a key role in mounting an innate immune response, a cellular stress response, and other antibacterial functions (Tan et al, 2026). We, therefore, assessed the energetics of macrophages and HUVECs after *E. faecalis* infection in the presence or absence of K21 and DFO. Monocyte-derived macrophages did not show any major changes in cellular ATP content in response to bacterial infection, K21 treatment, or DFO treatment, except for M1 macrophages, in which K21 induced an increase in cellular ATP levels (Fig S3E). Macrophages are metabolically hyperactive, and measuring subtle changes in cellular ATP is difficult due to their underlying variability. Hence, we repeated the same experiment in HUVECs. K21 suppressed cellular ATP content in HUVECs, whereas DFO up-regulated the same (Fig S3F). In response to bacterial infection, cellular ATP content in HUVECs is strongly up-regulated. This tendency was maintained even in the presence of K21. However, the total cellular ATP content was significantly lower than that of non-K21–treated cells. This might be due to a reduction in bacterial load within cells in the presence of K21. DFO-treated HUVECs did not show further increases in cellular ATP after bacterial infection. These results show that K21 has metabolic effects on cells that might contribute to its antibacterial properties.

### K21 induces mitophagy and lifespan extension in *C. elegans*

**Figure S5.**
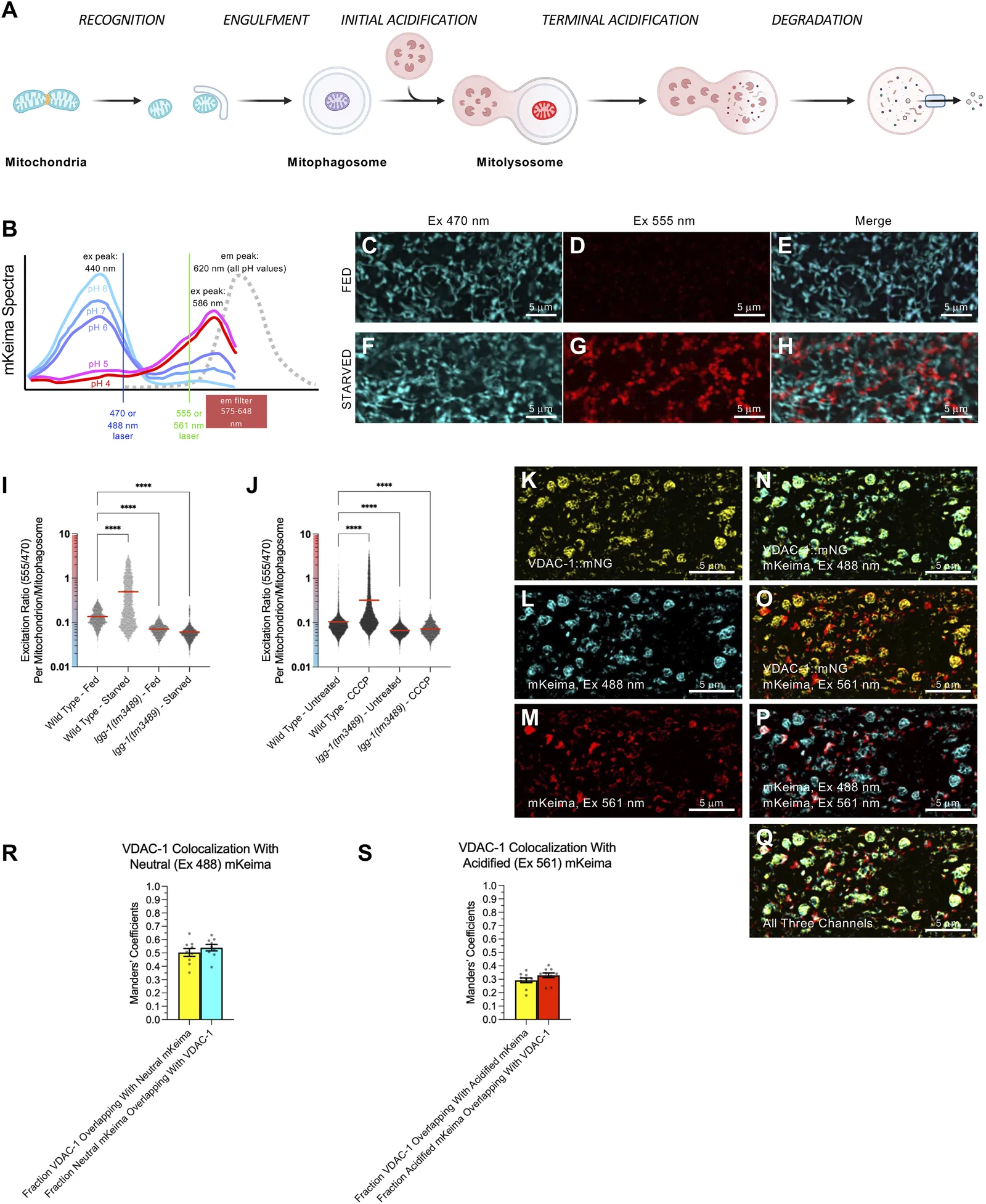
Validation and *in vivo* characterization of MitoKeima as a reporter of mitophagy in *C. elegans*. **(A)** Cartoon model of the mitophagy process. Mitochondria (cyan) targeted for mitophagy first undergo mitochondrial fission, followed by recognition by phagophoric membranes that begin to engulf the mitochondrial products of fission. Once enclosed within a complete autophagosome, mitophagic mitochondria are exposed to a low-pH environment (purple) that becomes increasingly acidic (red) after fusion with lysosomes. **(B)** The MitoKeima chimeric protein comprises a mitochondrial localization signal and mKeima, a pH-sensitive fluorescent reporter with dual pH-dependent excitation peaks. To visualize MitoKeima in non-mitophagic mitochondria, we excited samples using either a 470-nm or 488-nm laser, both of which overlap with the 440-nm excitation peak used by mKeima at pH values greater than 6 (cyan and purple curves). To visualize MitoKeima in mitophagic mitochondria, we excited using either a 555-nm or 561-nm laser, both of which overlap with the 586-nm excitation peak used by mKeima at pH values lower than 6 (magenta and red curves). The emission peak for both pH-dependent forms of mKeima is 620 nm (dotted gray line), which we collected with a bandpass filter (575–648 nm). **(C, D, E, F, G, H)** Fluorescent images of MitoKeima in the intestines of nematodes, as visualized at 620 nm during (C, F) excitation at 470 nm (non-mitophagic mitochondria) or (D, G) excitation at 555 nm (mitophagic mitochondria) under either (C, D, E) well-fed or (F, G, H) starved conditions used to induce mitophagy. (E, H) Merged images show both channels simultaneously, revealing the induction of mitophagic mitochondria in starved animals. **(I, J)** Fluorescence quantification of individual mitochondrial structures labeled with MitoKeima and detected by either 470-nm excitation or 555-nm excitation. The Y-axis plots the ratio of fluorescence emission values at 620 nm after excitation by either 555-nm (numerator) or 470-nm excitation (denominator). Individual dots show the ratio values for individual mitochondrial structures. The red bar indicates the mean value for the population described along the X-axis. ****P < 0.0001, ANOVA/Dunnett’s post hoc compared with (I) wild-type fed animals or (J) wild-type untreated animals. The mitophagic form of MitoKeima detected by 555-nm excitation after two known stimulating treatments for mitophagy (starvation and treatment with the mitochondrial membrane potential decoupler CCCP) is lost when the LC3/Atg8 ortholog LGG-1, which is critical for mediating all forms of autophagy, is knocked out in the *lgg-1(tm3489)* mutant, further validating MitoKeima as a faithful in vivo reporter of mitophagy. **(K, L, M, N, O, P, Q)** Super-resolution confocal fluorescence images of (K) VDAC-1mNeonGreen, (L) neutral (non-mitophagic) mitochondria labeled with MitoKeima, and (M) acidified (mitophagic) mitochondria labeled with MitoKeima. (N, O, P, Q) Different merged images from (K, L, M) as indicated. **(R, S)** Manders’ coefficients showing colocalization between VDAC-1mNeonGreen and either (R) neutral (non-mitophagic) mitochondria or (S) acidified (mitophagic) mitochondria labeled with MitoKeima. The bar in all images is 5 microns. Error bars indicate SEM. Representative replicates from three independent trials, 15–20 animals/genotype/condition/trial.

**Figure S6.**
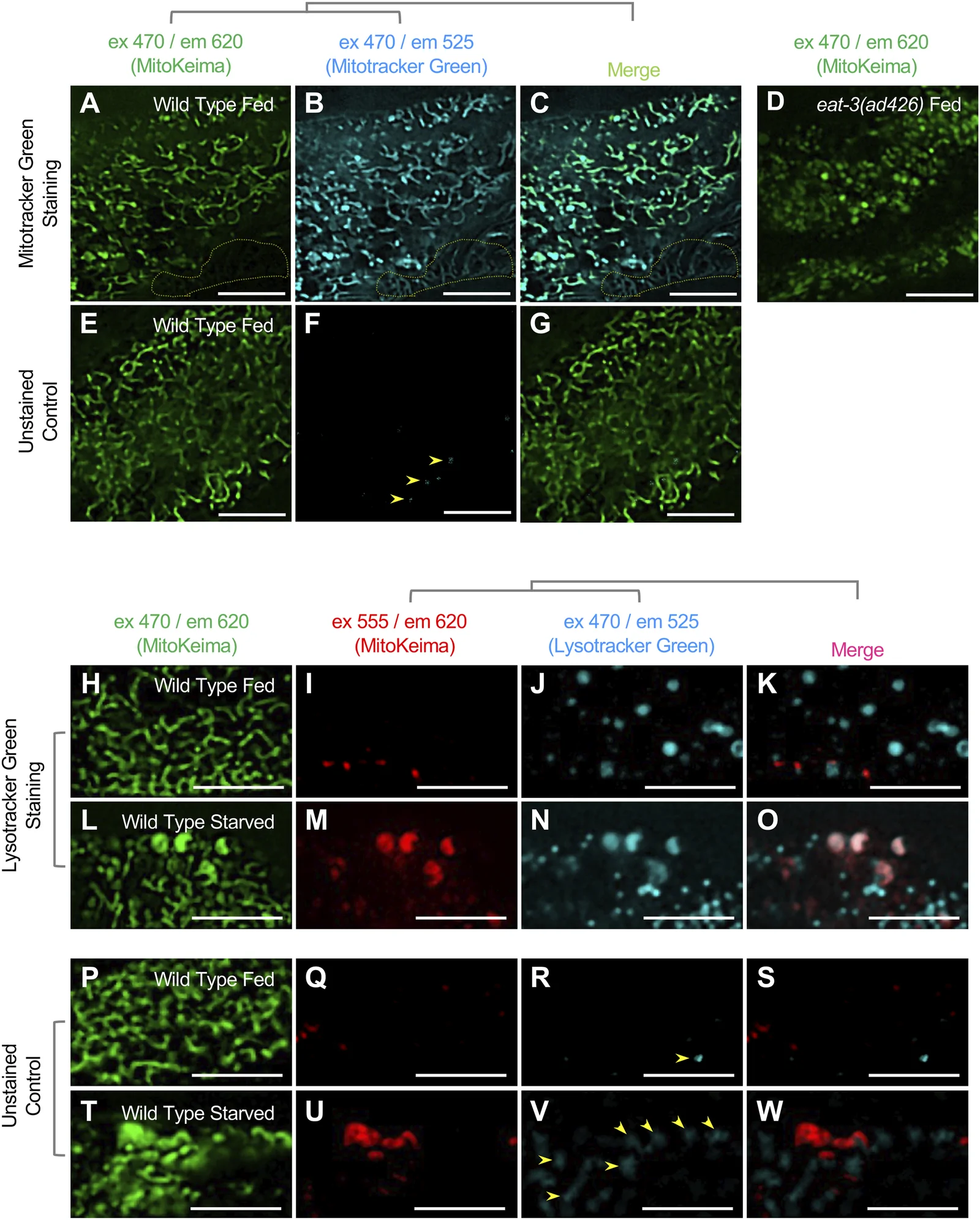
Validation of mitochondrial and lysosomal localization of MitoKeima in *C. elegans*. Fluorescent images of (A, D, E, H, L, P, T) non-mitophagic mitochondria (470-nm excitation, 620-nm emission), (I, M, Q, U) mitophagic mitochondria (555-nm excitation, 620-nm emission), (B) mitotracker green dye staining, or (J, N) lysotracker green dye staining in either (A, B, C, E, F, G, H, I, J, K, L, M, N, O, P, Q, R, S, T, U, V, W) wild-type nematodes or (D) *eat-3* mutants expressing MitoKeima under either (A, B, C, D, E, F, G, H, I, J, K, P, Q, R, S) well-fed or (L, M, N, O, T, U, V, W) starved conditions, with channel merges shown in (C, G, K, O, S, W). The non-mitophagic form of MitoKeima is present in elongated and networked structures in (A) the intestine, where it colocalizes with (B, C) MitoTracker Green. **(D)** Validating the mitochondrial localization of MitoKeima, the non-mitophagic form of this reporter loses its networking and assumes a globular shape when fusion is impaired in an *eat-3* mutant. **(M)** Starvation induces the formation of mitophagic MitoKeima-labeled mitochondria, as detected by 555-nm excitation, including in (O) large globular autolysosomes that colocalize with LysoTracker Green, further validating the reporter. Unstained controls are shown in (E, F, G) and (P, Q, R, S, T, U, V, W) to show low-level background autofluorescence (yellow arrowheads) under the same detection conditions for MitoTracker Green and LysoTracker Green (470-nm excitation, 525-nm emission) as the stained samples, showing robust signal. The yellow dotted line in (A, B, C) shows mitochondria in the hypodermis detected with MitoTracker Green but not expressing MitoKeima. Bar, 10 microns. Representative replicates from three independent trials, 15–20 animals/genotype/condition/trial.

Our scSLAM-seq and fluorescence microscopy analyses suggested that K21 acts through changes in mitochondrial fission and mitophagy, including in cells that are not involved in the immune system. Given the broad evolutionary conservation of these processes, we reasoned that K21 might similarly modulate them in a genetic model system, thereby providing in vivo support for our analysis. Thus, we turned to the in vivo model system *C. elegans*, as mitophagy can be observed in these nematodes using transgenes expressing MitoKeima, a protease-resistant, pH-sensitive fluorescent protein localized to the mitochondrial matrix that is used to monitor mitophagy through dual-excitation fluorescent imaging (Katayama et al, 2011; Lim et al, 2020; Fig S5A and B). We generated a transgene, *odIs167*, that expresses MitoKeima in the *C. elegans* intestine. Healthy (non-mitophagic) intestinal MitoKeima was detectable in the mitochondrial network with 470-nm excitation (Fig S5C); these labeled networks could be decorated with MitoTracker green staining (Fig S6A–G). Known inducers of mitophagy, including starvation and CCCP treatment, generated a second population of MitoKeima-labeled spherical structures detectable at 555-nm excitation (Fig S5D–H); these structures could be stained with LysoTracker green (Fig S6H–W). Formation of the acidified mitolysosomes labeled with MitoKeima could be blocked by mutations in *lgg-1*, which encodes a *C. elegans* homolog of the autophagy adaptor LC3 essential for mitophagy (Fig S5I and J). Both the non-acidified and acidified versions of MitoKeima colocalized with VDAC-1::mNeonGreen expressed from a knock-in of mNeonGreen at the endogenous *vdac-1* locus encoding the mitochondrial resident ortholog of VDAC (Fig S5K–S). These results demonstrate that MitoKeima is a faithful in vivo mitophagy reporter for the *C. elegans* intestine.

**Figure 6.**
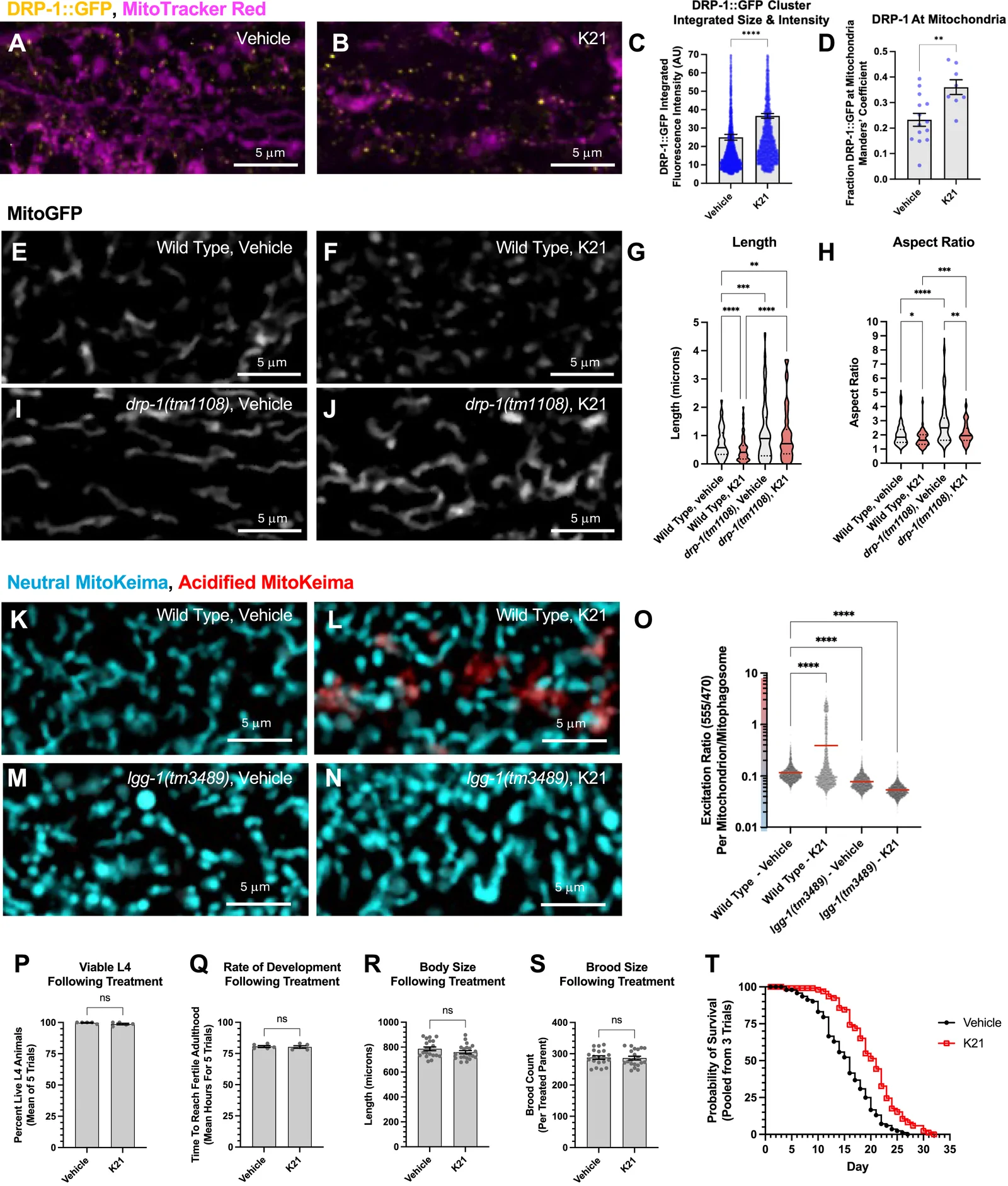
K21 induces mitophagy and lifespan extension in *C. elegans*. **(A, B)** Dual-channel image of intestinal DRP-1::GFP fluorescence (yellow) and mitochondria stained with MitoTracker Red dye (magenta) in (A) vehicle-treated or (B) K21-treated animals. **(C)** Quantification of mean DRP-1::GFP integrated density (sum of pixel values for each fluorescent cluster, reflecting the amount of DRP-1::GFP at each cluster). **(D)** Mean fraction of DRP-1::GFP colocalized with MitoTracker Red fluorescence (Manders’ coefficient) per animal. **P < 0.01, ****P < 0.0001, *t* test. **(E, F, I, J)** Fluorescence from mitochondria-localized GFP (MitoGFP) in the intestine of (E, F) wild-type or (I, J) *drp-1(tm1108)* mutant animals, either (E, I) vehicle-treated or (F, J) treated with K21. Violin plots showing quantification of mean individual mitochondrial (G) length or (H) aspect ratio (longest path divided by shortest path) from wild-type and *drp-1* animals treated with K21. **(K, L, M, N)** Dual-channel merged images of intestinal MitoKeima fluorescence combining emission from 470 nm excitation (neutral pH, non-mitophagic mitochondria colored cyan) and 555 nm excitation (acidified mitophagic mitochondria colored red) in (K, L) wild type or (M, N) *lgg-1(tm3489)* mutants of either (K, M) vehicle-treated or (L, N) K21-treated L4 nematodes. *P < 0.05, **P < 0.01, ***P < 0.001, ****P < 0.0001 ANOVA with Bonferroni post hoc test. **(O)** Ratio of fluorescence from excitation at 555 nm relative to that at 470 nm for individual mitochondria from five vehicle-treated versus five K21-treated animals. ****P < 0.0001, ANOVA with Dunnett’s post hoc test. The mean is indicated by the red bar. **(A, B, C, D, E, F, G, H, I, J, K, L, M, N, O)** Representative replicates from three independent trials. **(P)** Mean percentage over five trials (indicated as individual dots) of live animals reaching the L4 stage after hatching on vehicle-treated or K21-treated NGM plates. K21 does not impair viability. **(Q)** Mean number of hours over five trials (indicated as individual dots) required for vehicle-treated or K21-treated animals to reach fertile adulthood. K21 does not alter the rate of development. **(R)** Mean length as an indicator of body size at L4 for vehicle-treated or K21-treated animals. Dots indicate the values for individual animals. K21 does not alter body size or growth. **(S)** Mean number of eggs deposited over the fertile period of individual animals (dots) as a measure of brood size. K21 does not reduce fertility. “ns” indicates no difference based on a Student *t* test. **(T)** Kaplan–Meier survival curves for vehicle-treated (black circles) or K21-treated (red squares) animals, pooled from three independent trials (n = 128 and 151 animals, respectively). *P* < 0.0001, Mantel–Cox log-rank test. Bar indicates 5 microns in all images. Error bars indicate SEM. Source data are available for this figure.

To deliver K21 to *C. elegans*, we generated a colloidal suspension of micron-sized K21 particles and layered these over a bacterial OP50 lawn. Nematode eggs were hatched on this OP50/K21 mixture and allowed to feed and develop to the L4 stage, at which point they were assessed for MitoKeima fluorescence. DRP-1::GFP expressed from a GFP knock-in at the *drp-1* locus can be used to monitor DRP-1 recruitment to mitochondria (Lowry et al, 2015), appearing as small punctate foci that typically colocalize with mitochondria labeled with MitoTracker Red staining (Fig 6A). K21-treated nematodes showed increased DRP-1::GFP puncta size and fluorescence intensity (measured as an integrated fluorescence density per puncta; Fig 6B and C), as well as increased colocalization with MitoTracker Red compared with vehicle-treated controls (Fig 6D). Consistent with the increased recruitment of DRP-1 to mitochondria, K21-treated nematodes had shorter and rounder (i.e., smaller aspect ratio) mitochondria (labeled with a MitoGFP transgene) compared with vehicle controls (Fig 6E–H), consistent with increased mitochondrial fission. Indeed, mutations in *drp-1* resulted in elongated mitochondria and completely blocked the effects of K21 on mitochondrial size and shape (Fig 6G–J). Treatment of *odIs167* transgenic MitoKeima nematodes with K21 resulted in an increase in mitophagic structures labeled with the acidified form of MitoKeima; this increase was blocked by mutations in the Atg8-like autophagy receptor *lgg-1* (Fig 6K–O). Taken together, our results demonstrate that K21 induces DRP-1–dependent mitochondrial fission and LGG-1-dependent mitophagy, both in vitro and in vivo, and that these processes are conserved from nematodes to humans.

**Figure S7.**
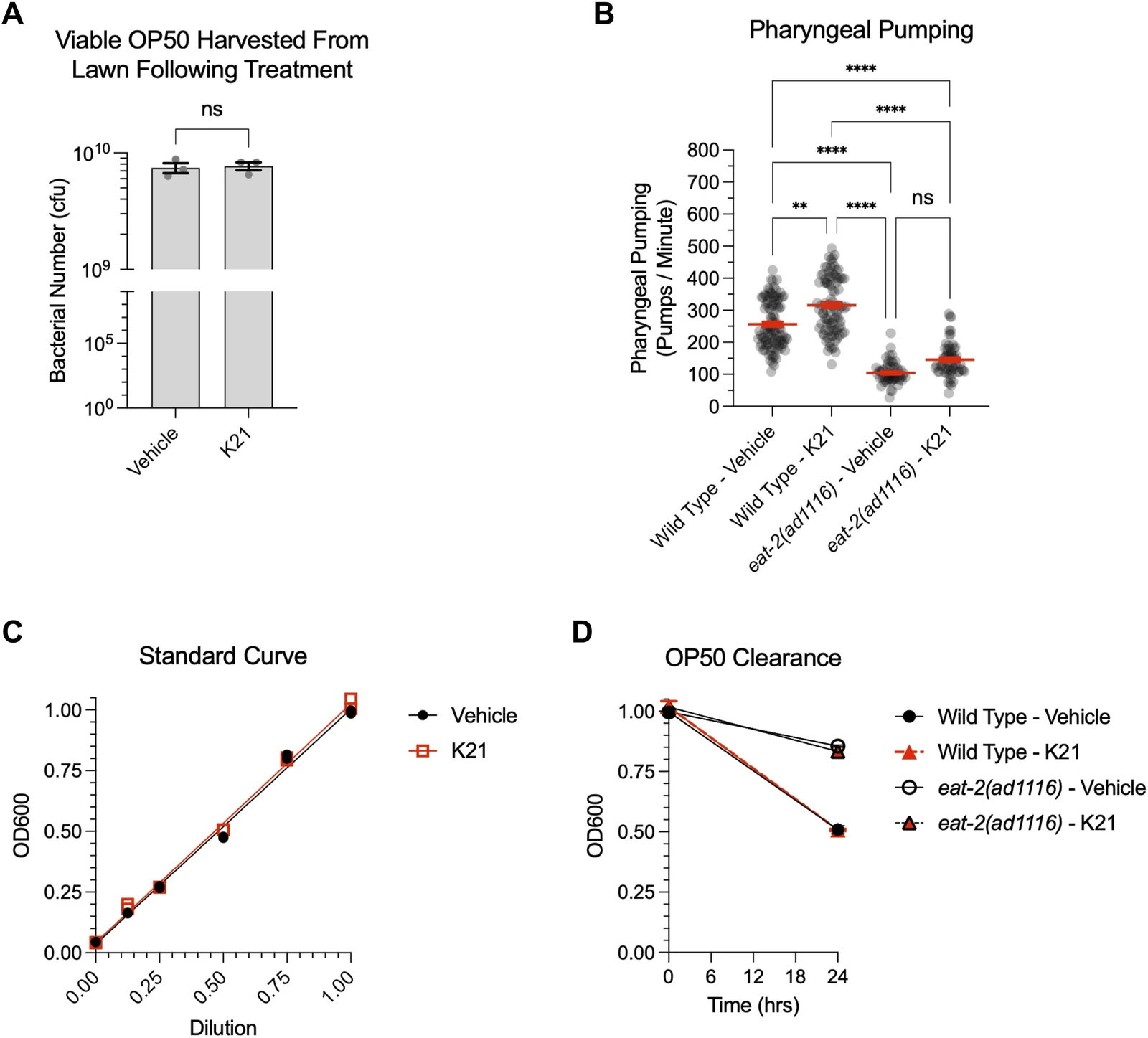
K21 does not impair bacterial food availability or feeding behavior in *C. elegans*. **(A)** OP50 lawns were treated with vehicle or K21, as per our standard treatment protocol for *C. elegans* on these bacterial lawns, and then the lawns were harvested from the NGM plates and titrated to obtain colony-forming units (cfu) representing culture density. No drop in OP50 viability was detected after K21 treatment. N = 3 biological replicates. **(B)** Nematodes were treated with K21 and then captured on video to quantify the rates of pharyngeal pumping, averaged over one min. Individual dots indicate pumping rates from individual animals pooled from three separate trials. The red bar indicates the mean, with error bars indicating the SEM. ****P < 0.0001, **P < 0.01 by ANOVA/Sidak for the individual comparisons shown. **(C)** OP50 lawns were treated with vehicle or K21 as per our standard treatment protocol, then harvested after three d, subjected to a dilution series, and analyzed by spectrophotometry at 600 nm to assess bacterial density to generate a standard curve. A linear relationship between culture amounts (dilution) and OD600 was detected at all values over three replicates. **(D)** OP50 lawns were treated with vehicle or K21 and then incubated with *C. elegans* as per our standard treatment protocol. At L4 stage, animals were each moved to two fresh plates with vehicle or K21. One plate was immediately harvested for bacteria and analyzed by spectrophotometry, as above. The other plate was grown for 24 h, then harvested and analyzed by spectrophotometry. Both K21-treated and vehicle-treated animals consumed their bacterial food source at the same rate, as shown by the average of three biological replicates. By contrast, mutants for *eat-2* are shown as a positive control for impaired pharyngeal pumping and feeding. SEM values were small (bars obscured by the circles and triangles).

**Figure S8.**
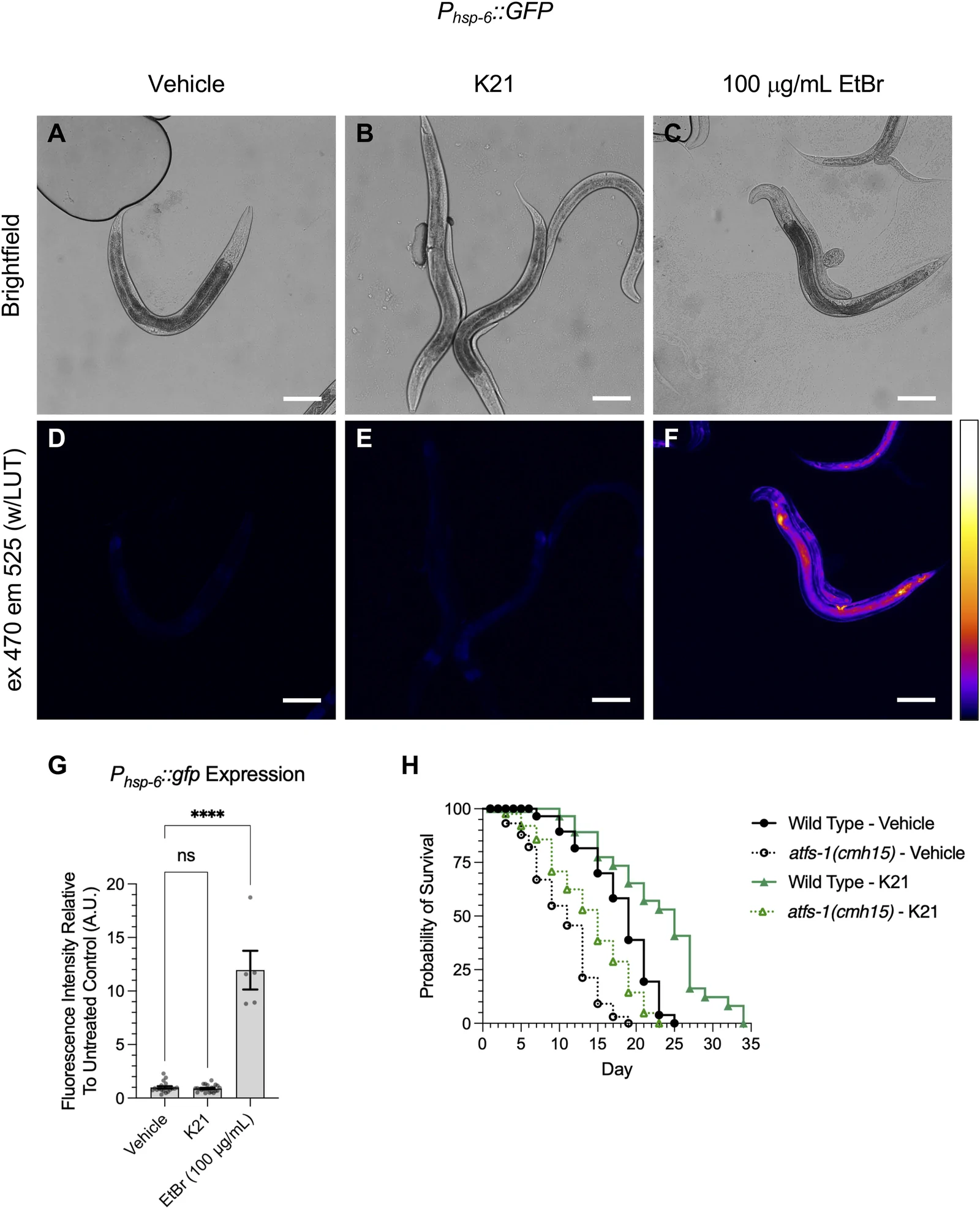
K21 extends *C. elegans* lifespan independently of the *atfs-1*-mediated mitochondrial unfolded protein response. Bright-field (A, B, C) and fluorescence (D, E, F) images of transgenic nematodes expressing GFP under the *hsp-6* promoter. Animals were treated with either (A, D) vehicle, (B, E) K21, or (C, F) ethidium bromide (EtBr), a known inducer of the UPRmt and *hsp-6* expression, used here as a positive control. GFP fluorescence was detected with 470-nm excitation and 525-nm emission, then converted to the indicated color-coded LUT. Bar, 50 microns. **(G)** Quantification of mean GFP fluorescence for individual L4 animals (dots), with error bars indicating SEM. ****P < 0.0001, ANOVA/Dunnett’s post hoc comparison to vehicle. K21 treatment did not activate *hsp-6* expression. **(H)** Kaplan–Meier survival curves for vehicle-treated (circles and black lines) or K21-treated (triangles and green lines) animals that were either wild type (solid circles and lines) or *atfs-1* loss-of-function mutant (empty circles and dotted lines), pooled from two independent trials (50 animals for each condition). *P* < 0.01 using Mantel–Cox log-rank for wild-type vehicle compared with the other three conditions, as well as *atfs-1* vehicle compared with K21 treatment. K21 can still induce lifespan extension in *atfs-1* mutants impaired for the UPRmt.

One relatively uninteresting explanation for why K21 induced mitochondrial fission and mitophagy is that the drug could kill the OP50 food source or impair nematodes’ ability to feed. We carefully quantified OP50 viability after treatment (Fig S7A), as well as the ability of K21-treated nematodes to pump their pharyngeal feeding organ (Fig S7B) and clear their plates of OP50 (Fig S7C and D); however, we did not see a reduction in these measurements, particularly compared with *eat-2* mutants, which served as a benchmark control of impaired feeding and reduced pharyngeal pumping. Treatment also did not impair survival, development, growth, or fertility (Fig 6P–S). Indeed, K21 extended the lifespan of treated nematodes (Fig 6T), consistent with increased mitophagy. Mitophagy can be induced by mitochondrial stress (Song et al, 2020; Picca et al, 2023), and stress-induced activation of the mitochondrial unfolded protein response (UPR^mt^) extends lifespan in *C. elegans* mutants with impaired mitochondrial function (Haynes & Hekimi, 2022). However, we did not observe activation by K21 of the established UPR^mt^ reporter *hsp-6::gfp* (Fig S8A–G), and mutants for *atfs-1*, the key activator of the UPR^mt^ (Nargund et al, 2012), still showed lifespan extension after K21 treatment (Fig S8H), suggesting that K21 does not induce mitophagy or lifespan extension by inducing the mitochondrial unfolded protein response.

### K21 induces changes in metabolic and stress response gene expression in *C. elegans*

**Figure 7.**
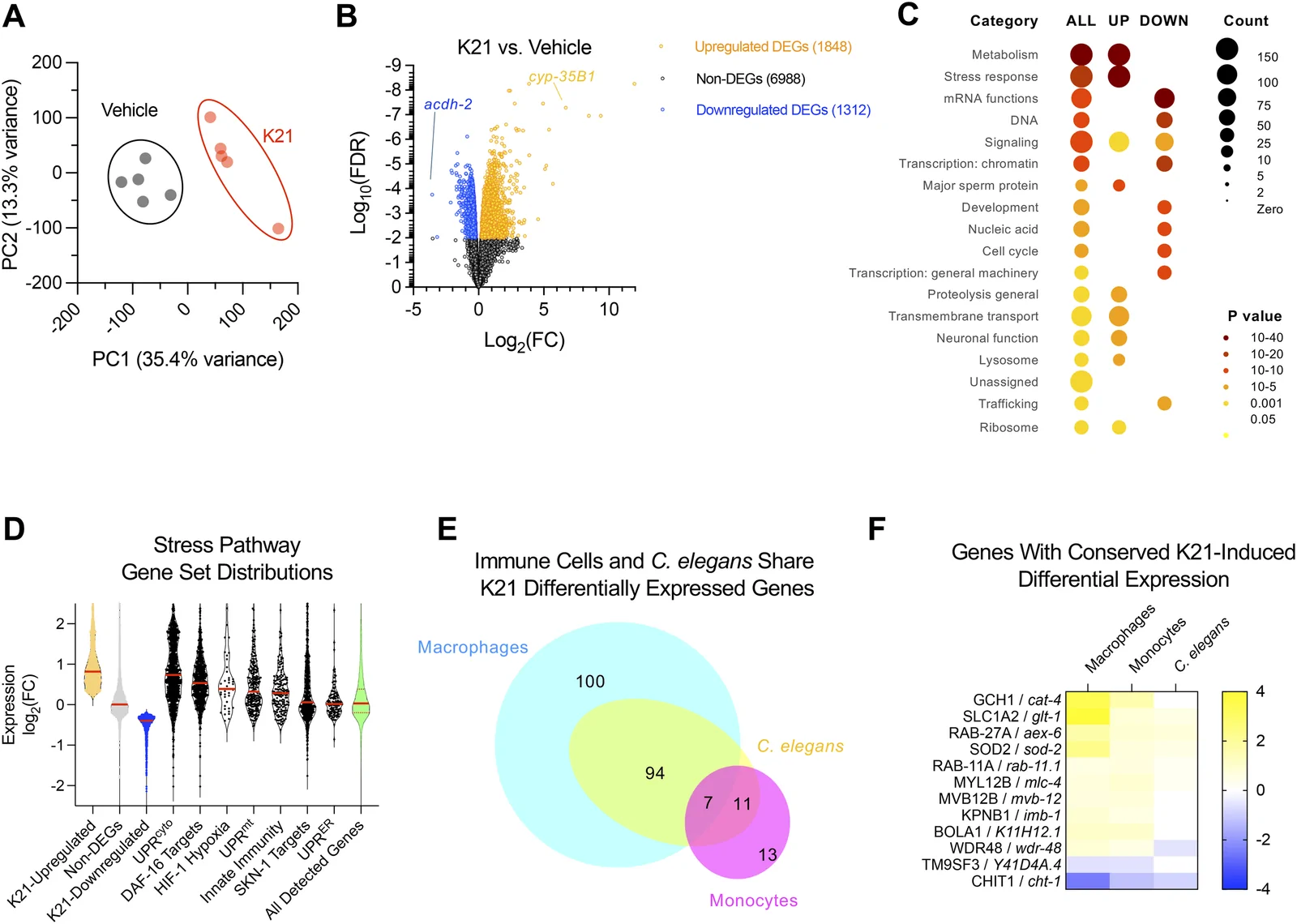
K21 induces changes in metabolic and stress response gene expression in *C. elegans*. **(A)** Principal component analysis of variance displayed along two PC dimensions for gene expression levels detected by RNA-seq for vehicle (gray circles) versus K21-treated (red circles) animals collected at the L4 stage. The two sets of samples cluster into distinct groups (outlined by ovals) primarily along the first PC. **(B)** Volcano plot of log_2_ fold changes in expression and log_10_ FDR rates for individual genes detected by RNA-seq in K21-treated L4 animals versus vehicle-treated animals. Genes up-regulated (gold) or down-regulated (blue) by K21 with an FDR<0.01 are indicated. The total number of genes in each category is indicated in the boxed figure legend. One up-regulated gene (*cyp-35B1*) and one down-regulated gene (*acdh-2*), which we validated with transgenic reporters, are highlighted. **(C)** WormCat category enrichment for either all DEGs induced by K21 or DEGs either up-regulated or down-regulated by K21 versus vehicle, as indicated by the column headers. Circle size and shading represent the number of genes identified in the category and the *P*-value for that category (adjusted for multiple comparisons), respectively, as per the legend. **(D)** Violin plots of mRNA expression levels (as log2 fold change) for individual genes for K21-treated versus vehicle-treated nematodes, separated into significantly (FDR < 0.01) up-regulated genes (gold), down-regulated genes (blue), genes showing no significant differential regulation (gray), and all detectable genes combined (green). Other columns show the distribution of expression values from RNA-seq for genes up-regulated in the indicated stress-response pathway gene set. The red solid line indicates the mean. **(E)** Venn diagram showing overlap between differentially expressed genes for K21 versus vehicle treatment from macrophage scRNA-seq (cyan), monocyte scRNA-seq (magenta), and *C. elegans* RNA-seq (yellow). Only genes showing orthology between humans and *C. elegans*, as well as differential expression in one or more datasets, are shown. **(F)** Hierarchically clustered heat map of log2 fold changes in mRNA levels from K21 versus vehicle-treated samples (as labeled in the columns) for only orthologous genes, and only for those showing differential expression in all three samples: macrophages, monocytes, and *C. elegans*. Source data are available for this figure.

**Figure S9.**
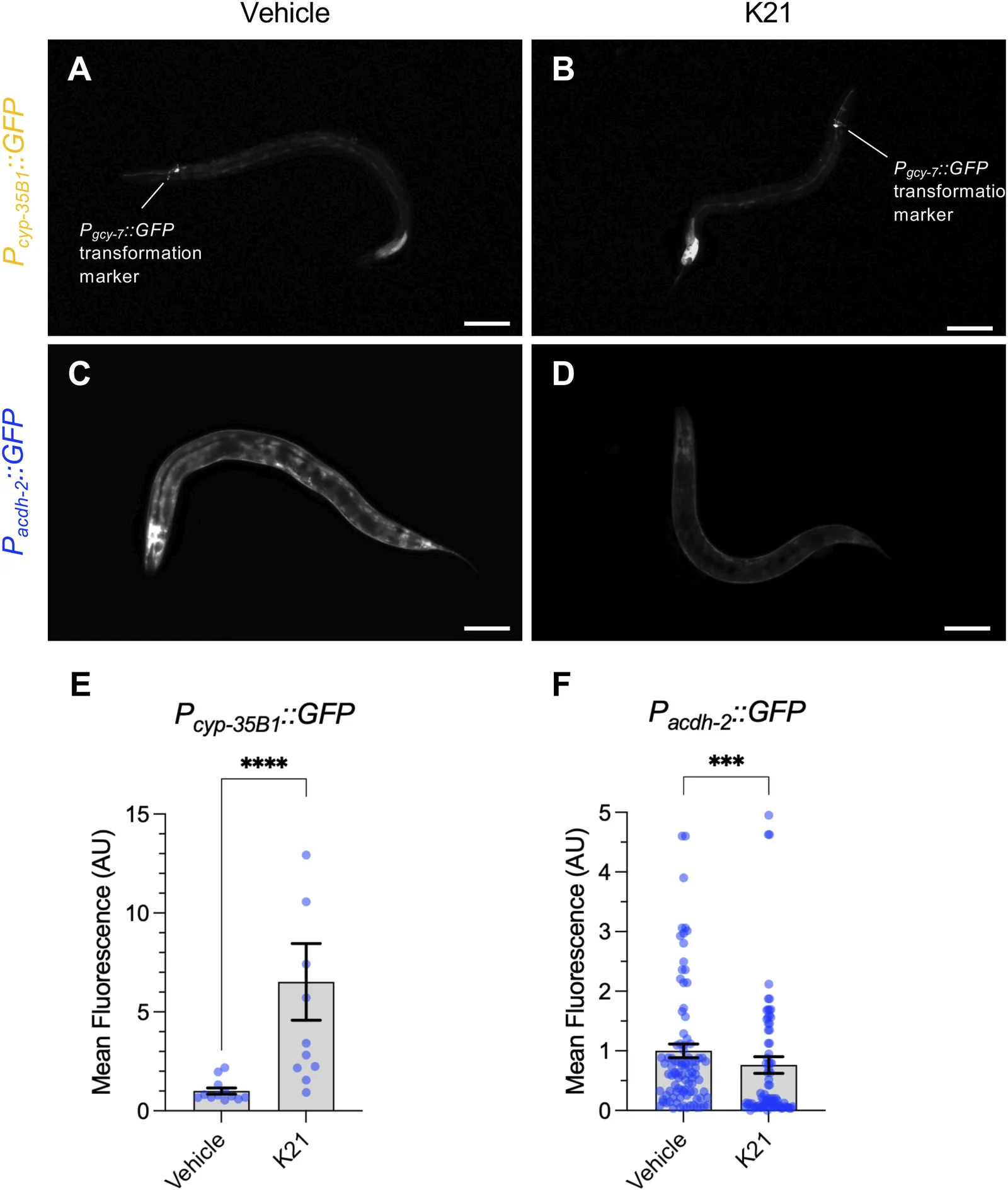
Validation of K21-induced altered genes from RNA-seq analysis using reporter expression in *C. elegans.* **(A, B, C, D)** Fluorescence images of L4 animals expressing GFP-based reporters for (A, B) *cyp-35B1* or (C, D) *acdh-2* are shown for (A, C) vehicle-treated versus (B, D) K21-treated L4 animals. Bar, 100 microns. **(E, F)** Quantification of mean GFP fluorescence for (E) *cyp-35B1* or (F) *acdh-2* transgenic animals for the treatment shown. Dots indicate the values for individual animals. ****P < 0.0001, ***P < 0.001 Mann–Whitney test. *P*_*gcy-7*_*::GFP* is a co-transformation marker expressed in the pharynx (as indicated by the line); its fluorescence was not included in the quantification of *P*_*cyp-35B1*_*::GFP*.

To understand how K21 might be altering *C. elegans* physiology, we used RNA-seq to obtain a transcriptional profile of K21-treated nematodes, as K21-induced changes in gene expression might reflect an underlying mechanism, and thus, be conserved with the changes observed in K21-treated mammalian cells. Principal Component Analysis (PCA) showed tight clustering across five independent biological replicates (Fig 7A), and we identified 1848 differentially expressed genes (DEGs) up-regulated by K21 and 1312 DEGs down-regulated by K21 using a false discovery rate (FDR) ≤ 0.01 (Fig 7B and Supplemental Data 1). We validated the K21-induced up-regulation of one of the up-regulated genes, *cyp-35B1*, from the RNA-seq analysis (Fig S9A, B, and E), quantifying fluorescence from a transgene expressing GFP from the *cyp-35B1* promoter (but not quantifying it from the co-injected transformation marker *P*_*gcy-7*_*::GFP*). Similarly, we validated the K21-induced down-regulation of one of the down-regulated genes, *acdh-2*, using a GFP-based transcriptional reporter transgene (Fig S9C, D, and F). GO term analysis using WormCat (Fig 7C) showed that K21 induced the expression of genes involved in metabolism, stress response, proteolysis, the endolysosomal system, and general mitochondrial and peroxisomal function. By contrast, K21 reduced the expression of genes associated with DNA replication, the cell cycle, transcription, mRNA processing, and translation.

Supplemental Data 1. Gene expression data from RNA-seq analysis of *C. elegans*.

The WormCat enrichment for overall stress-response genes prompted us to analyze individual stress-response pathways. We used GSEA (with an FDR cutoff of 0.05) to examine the distribution of gene sets known to be up-regulated by various stress-response pathways (Soo et al, 2021) in the K21 RNA-seq data. Genes known to be up-regulated by the heat shock response pathway (UPR^cyto^), DAF-16, HIF-1, the UPR^mt^, SKN-1, and innate immune response showed a significant enrichment in the K21-up-regulated genes (Fig 7D). Although the UPR^mt^ gene set was enriched in the full set of K21-up-regulated genes, its enrichment distribution was bimodal. Moreover, *hsp-6* expression did not show a statistically significant up-regulation by K21 in our RNA-seq data, matching our *hsp-6::gfp* analysis (Fig S8G) and suggesting that the full UPR^mt^ response was not activated by K21 treatment.

Finally, we reasoned that there might be a conserved core of K21-regulated genes conserved across evolution. We examined common K21-induced DEGs from scRNA-seq analyses of monocytes and macrophages, which together comprised the two largest cellular populations. We then used OrthoList2 to identify potential *C. elegans* orthologs of these DEGs in the nematode RNA-seq data (Kim et al, 2018). Nematode orthologs that showed K21-induced differential expression were identified for many of the DEGs induced by K21 in monocytes or macrophages (Fig 7E), and we observed differential regulation in the *C. elegans* orthologs of eight genes that also showed differential regulation in both monocytes and macrophages (Fig 7F). Taken together, these results demonstrate that K21 remodels metabolism, activates stress response pathways, and promotes mitophagy in *C. elegans*, with many of these aspects conserved from nematodes to humans.

## Discussion

Antimicrobial compounds demonstrating broad-spectrum effects against viral, bacterial, and fungal pathogens typically achieve these effects through surfactant properties and other physical and chemical mechanisms, including lipid membrane solubilization and protein denaturation. Such agents at high concentrations make for excellent disinfectants and topical treatments when formulations are carefully chosen to be nonirritating to human skin, but can have limited utility to treat infections that have breached barrier tissues (e.g., wounds) and/or formed microbial biofilms, or to treat internal infections (Ohadi et al, 2023; Dhadwal et al, 2024). A small number of compounds, including the quaternary ammonium silane compound K21, demonstrate broad-spectrum effects even at low concentrations, with minimal toxicity to human cells. Here, we provide evidence that K21 achieves its broad-spectrum effectiveness by modulating the innate immune system. In this study, pretreatment of monocyte-derived macrophages with K21 before infection enhanced their ability to combat bacterial load when subsequently challenged with *E. faecalis*. Using a powerful combination of cytokine profiling, cell-surface marker flow cytometry, and scRNA-seq to characterize the granular spectrum of macrophage differentiation and plasticity, we find that K21 promotes the differentiation of macrophages from monocyte populations without altering their overall polarization or their ability to secrete their respective cytokines. Moreover, K21 treatment increased the expression of pro-inflammatory factors in these cells. The combined effects of these changes likely explain the ability of K21 to combat a broad array of pathogens and accelerate wound healing (Gulve et al, 2016; Tweedy et al, 2017; John et al, 2019; Daood et al., 2021, 2025; Bapat et al, 2023).

Monocytes isolated from PBMCs can be differentiated into M0 GM-CSF–derived HMDMs or M0 M-CSF–derived HMDMs, depending on the cytokine used; for simplicity, we have termed these “naïve” macrophages (i.e., M1 and M2, respectively). These derived naïve macrophages can be activated and further polarized by treatment with additional cytokines, yielding several additional HMDM subtypes; for simplicity, we have termed these “activated” macrophages (i.e., M1, M2a, and M2c, depending on the expression of specific markers). K21 significantly induced two clusters of naïve macrophages characterized by their strong pro-inflammatory and pathogen-clearance properties. These two cell clusters (cluster N1 and cluster N9, Fig 3C) were not detected in activated macrophages, regardless of whether K21 was present, and they did not cluster when comparing non-K21 samples (Fig 1E and F).

Cells from cluster N9 resembled typical IFNα-induced M1 macrophages. They lacked expression of M2 markers, such as CD163L1 and PPARδ (Mukundan et al, 2009; Garrido-Trigo et al, 2023), and exhibited very low expression of other M2 macrophage markers, including FMN1 and DAPK1. However, activated M1-specific markers, such as CCL-5 and CD38, were strongly induced in the presence of K21 in these cells. From these observations, we concluded that these cells are M1 macrophages that were modulated into another M1 subtype capable of a stronger pro-inflammatory response.

Similarly, cells from cluster N1 were unique and resembled M-CSF-IFNγ-Pam3Cy5 cells in the SingleR annotation. M2-specific markers, such as CD163L1 and PPARδ, were detectable in these cells and were further suppressed by K21. These cells also had very strong expression of M2 macrophage markers, including FMN1 and DAPK1. Activated M1-specific markers, such as CCL-5 and CD38, were strongly induced in the presence of K21 in these cells. K21 further induced M1-specific proteins, such as CXCL8 and CXCL9, in this cluster of cells (Mantovani et al, 2004). These cell clusters also induced IL-17 expression, which is essential for M1 differentiation. From these observations, we concluded that these cells were originally M2 macrophages that had been switched towards an M1-like phenotype in the presence of K21. Both clusters were characterized by K21-induced expression of proteins such as SLC1A2, WARS1, SLAMF7, KYNU, SNX10, and CHI3L1, which are critical for initiating and amplifying a pro-inflammatory response necessary for pathogen clearance and the recruitment of other healing cell types. SLAMF7, in particular, is known to superactivate macrophages under chronic and inflammatory conditions (Simmons et al, 2022).

K21 induces the expression of kynureninase (KYNU), IDO1, and IDO2, key enzymes of the tryptophan-kynurenine pathway. IDO1 and IDO2 catalyze the cleavage of tryptophan to kynurenine, which is subsequently converted to anthranilic acid by KYNU. Tryptophan is an essential amino acid for the synthesis of serotonin, a neurotransmitter that plays a crucial role in regulating mood, sleep, appetite, and other functions. Tryptophan is also used for the synthesis of nicotinamide adenine dinucleotide (NAD^+^), a critical molecule in cellular energy metabolism. As another intermediate in the kynurenine metabolism of tryptophan, 3-hydroxykynurenine is converted to 3-hydroxyanthranilic acid by KYNU, leading to NAD^+^ generation. A delicate balance between these two pathways (tryptophan-serotonin versus tryptophan-kynurenine) is essential for human life.

The kynurenine pathway of tryptophan degradation is involved in T lymphocyte differentiation, influencing immune tolerance and inflammation (Stone & Williams, 2023). The IFNγ-IDO1-kynureine pathway plays a critical role in inducing autophagy in cervical cancer cells and promoting phagocytosis by macrophages (Yang et al, 2021). Kynurenine is a stable intermediate of the kynurenine pathway, and increased levels (along with increased IDO1 and IDO2) are often detected within the tumor microenvironment, suggesting their negative impact on tumor growth (Zuo et al, 2024). However, overexpression of KYNU should resolve this negative aspect of the kynurenine pathway by efficiently salvaging kynurenine. We did not detect KYNU within untreated macrophages. However, KYNU was strongly induced within the two pro-inflammatory macrophage clusters induced by K21. Increased IDO1 and IDO2 are indirectly linked to reduced serotonin levels, depression, and mood disorders. At the same time, the positive role of IDO1 and IDO2 in immune system development is also documented (Merlo et al, 2020; Choudhary et al, 2022). We observed increased IDO1 expression in T cells, B cells, monocytes, and macrophages in the presence of K21. However, IDO2 induction was observed only in macrophages, along with the induction of KYNU. These results suggest a specific positive role for the kynurenine pathway in the pro-inflammatory pathogen-clearing function of macrophages. These arguments could be tested in future studies using an animal model where serotonin levels could be measured after treatment with K21.

We also observed an increase in K21-induced M2-primed IFN-γ–stimulated IDO1^+^ macrophages, characterized by decreased CD274 expression and absence of IL4L1 and CD40/CD40L expression (cluster N1). These minor variations within macrophages could suggest significant alterations in their properties. Two clusters of naïve macrophages (clusters N0 and N2), which were identified as CXCL4 monocytes by SingleR annotation, resembled M4 macrophages and were suppressed in the presence of K21. M4 macrophages are a recently identified macrophage subset (Cai et al, 2020) with high expression of chemokine CXCL4, and they are known for their distinct characteristics and roles in various inflammatory and fibrotic processes. They are considered atherogenic due to their production of pro-inflammatory cytokines and reduced phagocytic capacity (Yu et al, 2024a). Using scRNA-seq and spatial transcriptomics, Coulis et al have recently identified a novel population of macrophages with high expression of galectin-3 and SPP1 in skeletal muscle fibrosis (Coulis et al, 2023). Our data showing that K21 suppresses these macrophages with high SPP1 and MMP9 expression could be beneficial for reducing fibrosis and sclerosis.

Metabolic differences, including the regulated balance between glycolysis, OXPHOS activity, and lipid metabolism, are critical drivers of macrophage differentiation, activation, and M1 versus M2 polarization. Mitochondria lie at the fulcrum of this balance, and we found that K21 treatment induces mitochondrial fission and mitophagy. K21 also regulates the expression of multiple genes involved in mitochondrial metabolism, morphology, and quality control, suggesting that mitochondria could be the mechanistic target of this agent. We observed that K21 could induce mitochondrial fission and mitophagy in many different cell types, including macrophages. We note that macrophages rely heavily on microautophagy; thus, it is likely our assessment of K21-induced mitophagy in these cells is an underestimate (Li et al, 2020; Lu et al, 2025). M1 macrophages rely on glycolysis, the pentose phosphate pathway, and fatty acid synthesis, whereas M2 macrophage metabolism is characterized by elevated fatty acid oxidation and OXPHOS activity (Galván-Peña & O’Neill, 2014; El Kasmi & Stenmark, 2015; Jha et al, 2015; Kumar et al, 2024); thus, the ability of K21 to modulate mitochondrial metabolism, dynamics, and quality control could explain how this agent promotes the antimicrobial abilities of these cells without dramatically altering their cytokine secretion profiles. Interestingly, agents known to induce mitophagy through stress (e.g., DFO, FCCP) enhanced bacterial phagocytosis and bacterial load within macrophages without causing a proportional decrease in bacterial infectivity (Fig S3C and D) or dramatic changes in cellular ATP (Fig S3E and F), whereas K21 not only induced mitophagy but also reduced bacterial infectivity and partially restored cellular ATP levels in infected cells, suggesting a more homeostatic mechanism of action compared with that of DFO or FCCP (Fig S3E and F). Beyond inducing mitochondrial fission and mitophagy, DFO treatment activates HIF-1, which promotes immunometabolic gene expression and antimicrobial properties, and chelates iron, which restricts pathogen growth, complicating interpretation (Teran et al, 2022; Kotey et al, 2024). FCCP treatment can impair the ability of macrophages to clear bacteria depending on the specific model and stimulation conditions, and PINK1/Parkin–mediated mitophagy has been suggested to protect against macrophage-promoted fibrosis (Bhatia et al, 2019; Patoli et al, 2020). By contrast, K21 treatment might provide a more nuanced approach for testing the relationship between mitophagy and macrophage differentiation, polarization, and activation.

Aside from mitochondrial quality control and ATP production, differences in macrophage metabolism along the M1/M2 polarization axis also affect these cells’ ability to produce metabolites and the small-molecule signals that help fight infections and communicate between innate and adaptive immune cells. Indeed, K21-mediated immune cell remodeling was observed only in a complex multicellular co-culture environment. Indeed, T cells and NK cells, rather than macrophages, were the first cells to show gene expression changes in our SLAM-seq analysis, suggesting that an interplay among various cell-derived components is required for K21 to act. The example of how K21 can modulate innate immunity below the surface of cytokine secretion and cell-surface marker expression highlights the need to understand the differentiation and plasticity of innate immune cells using detailed and granular systems approaches, including single-cell RNAseq and transcriptomics.

Interestingly, the effects of K21 on mitochondrial fission and mitophagy were observed in the model organism *C. elegans.* Why compare the effects of K21 on *C. elegans* versus PBMCs? If the target of K21 action is mitochondria and their associated quality control mechanisms, then the effects of K21 observed in human tissue culture cells should be deeply conserved across animal phyla, as well as observed in a whole-animal model. Our *C. elegans* experiments satisfied both of these criteria, demonstrating an in vivo context shared by our observation of the effects of K21 on PBMCs in culture. Nematodes lack macrophages and other well-established components of human innate and adaptive immunity; nevertheless, genes associated with innate immunity in *C. elegans* were up-regulated by K21 treatment. Although the effects of K21 on immunity may not be analogous to those in humans, we found that K21 treatment extended the lifespan of *C. elegans* without compromising survival or fertility, consistent with increased activity of mitophagy as a quality control mechanism that boosts health through hormesis (Bauer et al, 2024; Calabrese et al, 2024). One less interesting explanation for why K21 treatment increases lifespan could have been that K21 impairs pharyngeal pumping and feeding, leading to starvation; however, we did not observe a decrease in feeding rates in treated animals, ruling this explanation out. Indeed, K21-treated animals showed slightly higher rates of pharyngeal pumping compared with untreated animals, which could be explained by changes in mitochondrial function or metabolism induced by K21 treatment. As nematodes and humans are separated by nearly a billion years of evolution, the broadly conserved effects of K21 suggest that its molecular or pharmacological target is deeply conserved and not specific to immune cells.

Although the specific molecular target of K21 remains to be elucidated, our analysis of K21-modulated mitochondrial dynamics in human immune cells, non-immune cells, and *C. elegans* cells points to some universal principles for its cellular mechanism of action. K21 induces Drp1/DRP-1–dependent mitochondrial fission, including the accumulation of Drp1/DRP-1 protein at mitochondria. It also induces mitophagy mediated by the autophagy receptor protein LC3β/LGG-1. K21 comprises four fatty acid chains attached to a core group of quaternary ammonium silanes, forming a lipophilic cation. Thus, it is reasonable to speculate that the molecule might be targeted to mitochondrial membranes, similar to other lipophilic cations (Battogtokh et al, 2018). Specific lipid species play critical roles in recruiting both fission and mitophagy factors to mitochondria (Frohman, 2015); K21 might be mediating mitophagy in a similar fashion. K21-induced mitophagy appears distinct from that induced by DFO (a hypoxia mimetic that operates through up-regulation of BNIP3L/NIX) or FCCP (a mitochondrial membrane polarization decoupler that operates through activation of PINK1/Parkin), both of which are characterized by cellular and mitochondrial stress. Transcriptomic analysis suggests that K21 treatment has modest effects on certain stress-response pathways, raising the possibility that it acts as an inducer of mitohormesis. Indeed, the K21-induced increase in *C. elegans* lifespan is consistent with this interpretation. Given that the mitochondrial organelle is fundamentally conserved and plays critical roles in metabolism, immunity, and lifespan, we contend that a deeper understanding of how K21 acts on or through mitochondria should facilitate the development of additional powerful therapeutics.

## Materials and Methods

### HMDM generation and polarization

Macrophages were generated from human PBMCs of healthy donors. For the initial comparison of the effects of immune cells and HFFs on macrophage differentiation, PBMCs were collected from the EDTA blood of three healthy blood donors. Peripheral blood for these studies was obtained with ethical approval from the Riga Stradiņš University (RSU) Research Ethics Committee (2-PĒK-4/784/2025).

### PBMC isolation

Blood samples from apparently healthy individuals were collected in EDTA tubes (Cat# 367525; BD Vacutainer). PBMCs were isolated via centrifugation in a density gradient using Histopaque-1077 (Cat# 10771-500ML; Sigma-Aldrich) at 550*g* for 35 min at 24˚C. After which, the PBMC layer was gently aspirated using a Pasteur pipette and washed with PBS at 800*g* for 10 min at 24°C, then at 300*g* for 10 min at 24°C.

### Isolation of CD14^+^ monocytes by positive selection

PBMCs were pelleted in 80 μl of Miltenyi MACS buffer (1:20 dilution of Miltenyi MACS BSA Stock Solution Cat# 130-0910376 in Miltenyi autoMACS Rinsing Solution Cat# 130-091-222) per 10^7^ cells. 20 μl of CD14 Beads per 10^7^ cells were added to the cells, mixed thoroughly, and incubated for 15 min at 4°C. The cells were then washed and resuspended in 500 μl of MACS buffer (Phosphate-buffered saline (PBS), pH 7.2, containing 0.5% bovine serum albumin (BSA) and 2 mM EDTA). The cells were incubated with CD14 microbeads (human, Cat. 130-050-201). The bead-conjugated cell suspension was loaded into a Miltenyi MS separation column (Cat. No. 130-042-201) on a MiniMACS separator and rinsed three times with 500 μl of buffer. Finally, cells were eluted with 1 ml of elution buffer using a plunger. The process was repeated on a second MS column to increase cell purity. For M1 macrophage differentiation, 50 ng/ml GM-CSF (Cat# GF304; Sigma-Aldrich) was added to the culture media, and for M2 macrophage differentiation, M-CSF 50 ng/ml (Cat# SRP3110; Sigma-Aldrich) was added to the culture medium.

### Isolation of total monocytes, including copurified immune cells

PBMCs were resuspended in Monocyte Attachment Medium (#C-28051; PromoCell) and incubated for 1–2 h at 5% CO2 and 37°C in the incubator to facilitate cell attachment. Adherent cells were washed thrice intensively with warm Monocyte Attachment Medium to loosen non-adherent cells. M1 or M2 Macrophages were generated using M1 or M2 Generation Medium XF, respectively (PromoCell M1 #C-28055 containing GM-CSF or M2 #C-28056 containing M-CSF).

### Monocyte to macrophage polarization in the presence or absence of fibroblasts

For monocyte differentiation, 150,000–200,000 monocytes were seeded per well in a 24-well plate, with an 18 mm coverslip for microscopy analysis or without for other studies. K-21 at a 0.28 μM concentration was added to the desired wells, whenever required. Primary HFFs (50,000 cells/well) were added to the culture on day 6, as and when required. LPS (10 ng/ml) was added to naive macrophages on day 6 for cell stimulation for cytokine secretion. For activated M1 differentiation, cells were treated with GM-CSF + IFNγ (25 ng/ml) (Cat# SRP3058-100UG; Sigma-Aldrich) + LPS (10 ng/ml) on day 6 and were allowed to grow for 4 more days. For activated M2a differentiation, cells were treated with M-CSF, IL-4 (25 ng/ml) (Cat# SRP3093-20UG; Sigma-Aldrich), and IL-13 (25 ng/ml) (Cat# SRP3274-10UG; Sigma-Aldrich) and allowed to grow for an additional 4 d. LPS was added to the activated macrophages on day 9 to stimulate cytokine production. On day 10, supernatant was collected for cytokine measurement, and the remaining cells were detached for FACS staining. Cells without LPS stimulation were used as controls. On day 10, macrophages were washed with PBS and detached in the presence of 1 ml Accumax solution (#SLCR6580; Sigma-Aldrich) for 5–10 min at 37°C for further studies.

### Monocyte to macrophage differentiation for scRNA-seq

For single-cell RNA-seq, we used heterogeneous donor-derived PBMCs. PBMCs were prepared from healthy blood donors as a byproduct of platelet concentrates obtained using leukoreduction system chambers, kindly provided anonymously by the Department of Transfusion Medicine at the University Hospital Würzburg, in accordance with the guidelines of the Ethics Committee of the Medical Faculty of the University of Würzburg. PBMCs were isolated using a density gradient (Histopaque-1077, #1077-100ml; Sigma-Aldrich), washed twice with PBS, and resuspended in Monocyte Attachment Medium (#C-28051; PromoCell). The frequency of mononuclear cells was analyzed by Flow cytometry (Attune Nxt Flow Cytometer). As the frequency of mononuclear cells exceeded 25% of the total, 1.5 × 10^6^ cells/cm^2^ were seeded into 6-well plates (1.4 × 10^7^ per well) and incubated for 1–2 h at 5% CO2 and 37°C to allow cell attachment. Adherent cells were washed thrice intensively with warm Monocyte Attachment Medium to loosen non-adherent cells.

One group was treated with 0.28 μM K21 and the control group received vehicle control. Incubation at 37°C for 6 d yielded immature macrophages. On day 6, M1 immature macrophages were activated into classical M1 macrophages by adding 50 ng/ml IFN-γ (#11343534; Immunotools rh-IFNγ) and 10 ng/ml LPS (Sigma-Aldrich, lipopolysaccharides from *Escherichia coli* O111:B4 #L5293), or they remained without an activation stimulus. M2 immature macrophages were activated into M2a macrophages by adding 20 ng/ml IL-4 (rh IL-4, #SRP3093; Sigma-Aldrich) or into M2c macrophages by adding 20 ng/ml TGFβ (#7754-BH/CF; R&D rhTGFβ), respectively. The cells were further incubated until day 10 in the presence of K21 or in its absence as a control.

### Flow cytometry

For most of the flow cytometry analyses shown in this study, macrophages were rinsed with PBS to remove residual media. 500 μl of ice-cold EDTA (10 mM) was added to each well, and the wells were incubated at 4°C for 30 min to 1 h. Cells were pipetted off the surface and transferred to a 96-well plate. Cells were centrifuged at 550*g* for 5 min to remove the debris. Cells were then blocked with 50 μl Fc block (1:200) at 4°C for 30 min. Cells were then centrifuged at 550*g* for 5 min to remove the blocking solution and washed with 200 μl of FACS buffer; 50 μl of antibody cocktail was added to each well, and the wells were incubated at 4°C for 30 min. Afterward, cells were washed twice with 200 μl of FACS buffer and resuspended in 200 μl of FACS buffer. Protein expression was measured using BD FACS Aria III. The following antibodies were used for flow cytometry (Table 1).

**Table 1.** Details of antibodies used for the flow cytometry experiments.

Antibody	Channel	Dilution	Cat. number	Lot	Clone
CD163	BV421	1:100	333611	B425652	GHI/61
CD80	BV650	1:100	305227	B436598	2D10
CD33	PE/Cy7	1:300	366617	B417457	P67.6
CD206	AlexaFluor 647	1:200	321116	B410991	15-2
CD14	APC/Cy7	1:200	325619	B363390	HCD14
CD11b	BV510	1:100	301334	B449280	ICRF44
Fc Block (1:200)		1:200	130-059-901	5241102824	​

For flow cytometry of the same batch of cells processed for single-cell RNA-seq, macrophages were first blocked using the blocking antibody 2.4G2 for 15 min at 4°C. Next, macrophages were labeled with saturating amounts of human antibodies in FACS buffer (PBS containing 0.1% BSA and 0.02% NaN_3_) for 15 min at 4°C. The following antibodies were used against the cell-surface antigens: CD66b, CD11b, CD14, CD16, CD163, CD80, CD206, CD33, CD11c, CD1c, and CD209. Stained macrophages were directly measured using the Attune Nxt Flow Cytometer (Thermo Fisher Scientific). The following antibodies were used for flow cytometry (Table 2).

**Table 2.** Details of antibodies used for the flow cytometry experiment using the same samples processed for scRNA-seq.

Antibody	Distributor	Cat. number	Clone	Dilution
CD66b A700	BioLegend	305114	G10F5	1:25
CD11b BV510	BioLegend	301334	ICRF44	1:19
CD14 FITC	R&D	FAB3832F-100	FAB3832F	1:21
CD16 BV605	BioLegend	302040	3G8	1:21
CD163 PerCp	R&D	FAB1607C	215927	1:12
CD80 PE	R&D	FAB140P-100	37711	1:30
CD206 APC	R&D	FAB25342A	685641	1:15
CD33 PE-Cy7	BioLegend	303434	WM53	1:21
CD11c APC-Cy7	BioLegend	337218	Bu15	1:30
CD1c PE-Dazzle 594	BioLegend	331532	L161	1:30
CD209 BV421	BioLegend	330118	9E9A8	1:25

### Single-cell RNA-seq and single-cell SLAM-seq

For single-cell RNA-seq analysis, monocytes were collected from three individuals and combined for the final assay. One million cells per condition were washed with PBS on day 10 of the experiment and then detached in the presence of 1 ml Accumax solution (#SLCR6580; Sigma-Aldrich) for 5–10 min at 37°C. Cells were washed once again with PBS, fixed in a PBS:methanol mixture (1:4), and stored at −80°C until subsequent use. On the day of the experiment, methanol-fixed cells were thawed on ice and rehydrated in 1X PBS for 30 min on ice, then filtered with a 40-μm cell strainer. Cells were barcoded using MULTI-seq Lipid Modified Oligos (#LMO001; Sigma-Aldrich). After cell counting, single-cell suspension samples were pooled into four separate groups (Activated macrophages, -K21; Activated macrophages, +K21; naïve macrophages, –K21; and naïve macrophages, +K21). Ten thousand cells per sample were loaded on a Chromium Controller (10× Genomics). The scRNA-seq libraries were constructed using the Chromium Single Cell 3′ v3.1 Reagent Kit according to the manufacturer’s guidelines (10× Genomics). cDNA libraries were uniquely sample-indexed and pooled for sequencing. A HiSeq (Illumina) sequencing run was used to sample-balance on a NovaSeq 6000 SP flow cell (Illumina), targeting >25,000 reads/cell.

For single-cell SLAM-seq, freshly isolated PBMCs were grown for 2 h in the presence (0.2 μM K21) or absence of K21, along with 200 μM 4sU added to the cell culture media at the time of K21 addition, which allowed 2 h of 4sU incorporation into the cells. PBMCs were washed and fixed in methanol as mentioned above. The methanol fixation was allowed to proceed for 30 min at −20°C. Then, 10 mM iodoacetamide (IAA) was added to each methanol-fixed cell, and the mixture was left at 4°C overnight with gentle agitation. The next day, cells were centrifuged down and resuspended in quenching buffer containing DTT (DBPS, 0.1% BSA, 1 U/μl RNaseOUT, and 100 mM DTT) for 5 min at room temperature. Cells were then centrifuged down and resuspended in resuspension buffer (DBPS, 0.01% BSA, 0.5 U/μl RNaseOUT, 1 mM DTT). Cells were then passed through a 0.4-µm filter, counted, and analyzed by 10X Genomics microfluidics. PBMCs with and without K21 were loaded into two separate lanes on the same chip, targeting 5,000 cells per lane. The scRNA-seq libraries were constructed using the Chromium Single Cell 3′ v3.1 Reagent Kit according to the manufacturer’s guidelines (10× Genomics). cDNA libraries were uniquely sample-indexed and pooled for sequencing. A HiSeq (Illumina) sequencing run was used to sample-balance on a NovaSeq 6000 SP flow cell (Illumina), targeting >25,000 reads/cell.

### Data processing and visualization

Experimental data were demultiplexed using the Cell Ranger Single-cell Software Suite, mkfastq command wrapped around Illumina’s bcl2fastq. Cell Ranger software was used to perform demultiplexing, alignment, filtering, barcode counting, UMI counting, and gene expression estimation for each sample according to the 10× Genomics documentation (https://support.10xgenomics.com/single-cell-gene-expression/software/pipelines/latest/what-is-cell-ranger). The gene expression estimates from each sample were aggregated using Cellranger (cellranger aggr) to compare experimental groups with normalized sequencing depth and expression data.

The sequencing data were processed using the CellRanger (v8.0.0) GEX+ADT pipeline, which conducts QC, cell filtration, read alignment, and generation of feature-barcode matrices. The reads were aligned to the human reference genome (GRCh38). To obtain higher numbers of usable cells, we set the parameter “--expect-cells=10000” within CellRanger, which resulted in slightly higher numbers of cells: 5,472, 5,315, 6,303, and 7,928 cells, respectively. The Seurat (v4.1, https://satijalab.org/seurat/) R package was used to calculate QC matrices and filter cells and features. For cell filtering (https://github.com/satijalab/seurat/issues/3396), we first removed empty droplets. Subsequently, we also removed low-quality cells with unique feature counts greater than 8,000 or less than 200 (nFeature_RNA more than 200, nFeature_RNA less than 8,000). Furthermore, low-quality or dying cells that exhibited extensive mitochondrial contamination (percent.mt >20) were removed. Genes expressed in at least three cells were retained for filtering (Hao et al, 2021). Quality control (QC) was based on (1) nFeature_RNA: number of unique genes detected in each cell; (2) nCount_RNA: total number of molecules detected within a cell; (3) percent. mt: percentage of reads that map to the mitochondrial genome. Mitochondrial QC metrics were generated by calculating the percentage of counts originating from a set of features (the set of all genes starting with “MT-,” mitochondrial genes).

Demultiplexing was performed to identify the 8-base Multiseq barcodes and assign reads to presumed Macrophage subtypes (e.g., M1, M2a). Seurat (v4.1) R package was used to process and analyze the data. We first analyzed the data for each of the four samples separately, focusing on the demultiplexing outcome. The feature expression count for each cell was normalized by the total count. The normalized count for each cell was multiplied by a scale factor (10,000), then log-transformed. The expression measure was then adjusted to account for variation in mitochondrial gene proportions. To identify highly variable features, the 2,000 most variable features were selected. Afterward, cell clustering and principal component analysis (PCA) were performed. Uniform Manifold Approximation and Projection (UMAP) and clustering were performed to identify clusters. Graph-based cell clustering was performed. The PBMC SLAM-seq data were processed using the GrandR package, developed by the Erhard group (Rummel et al, 2023).

### GSEA for scRNA-seq data

GSEA analysis for each major annotated cell type was carried out using the GO database, including Biological Process (BP), Cellular Component (CC), and Molecular Function (MF). GSEA Reactome analysis was carried out using the C2 signature dataset from MSigDB. Differentially expressed genes were identified using the FindMarkers() function in Seurat with parameters logfc.threshold=0 and return.thresh =1. The log2-fold change calculation from FindMarkers() was used for GSEA. GSEA was performed using the fgsea (version 1.30.0) and clusterProfiler (version 4.10.0) packages.

### Pseudotime trajectory analysis

CytoTRACE R package (version 0.3.3) was used to predict the differentiation state of cells and absolute development potential for the samples (Gulati et al, 2020). CytoTRACE scores range from 0 to 1, with higher scores indicating greater stemness (less differentiation). Monocle3 was then used to construct cell trajectories and predict pseudotime (Trapnell et al, 2014). The root cell was identified using the function “get_earliest_principal_node()” provided in Monocle3. Using the root cell (marked with cell “1”) as a starting point, we constructed the cell trajectory and predicted pseudotime.

### Cell culture for mitophagy studies

U2-OS cells were purchased from ATCC and cultured in RPMI 1640 medium supplemented with 10% (vol/vol) FBS and 200 units/ml penicillin-streptomycin (Schreiner et al, 2020). TERT2-immortalized HUVEC cells were purchased from Evercyte, Austria (Cat. CHT-006-0008) and were grown in EBM basal medium (Cat#CC-3121; Lonza) supplemented with components of the EGM SingleQuot Kit (Cat# CC-4133; Lonza: BBE, hEGF, hydrocortisone, and ascorbic acid), 10% FBS, and 20 μg/ml G418. Primary HFFs (ATCC SCRC-1041) were maintained in primary fibroblast basal medium (ATCC PCS-201-030) supplemented with recommended growth factors and antibiotics (ATCC PCS-201-040). All cell lines were cultured at 37°C with 5% CO_2_. Cells carrying stable GFP or RFP expression within mitochondria were developed as mentioned before (Chowdhury et al, 2017). DFO treatment involved 260 μM DFO for 24 h. K21 treatment involved 1.072 μM K21 for 24 h.

### Average mitochondrial area and mitochondrial number analysis

The average mitochondrial surface area was measured in U2-OS and HUVEC cells expressing soluble GFP within mitochondria, which were developed using previously described protocols from our laboratory (Chowdhury et al, 2017). Software and a modified algorithm for measuring mitochondrial size and number were previously described in detail by us (Chowdhury et al, 2017). The Pearson’s correlation coefficient was calculated using the coloc2 plug-in of ImageJ.

### Immunofluorescence microscopy

For macrophage staining, M1- and M2-differentiated cells grown on 18 mm coverslips were washed with 1X PBS (+Mg and Ca_2_) on day 10 of polarization. Cells were then fixed with 4% formaldehyde, permeabilized with 0.1% Triton X-100 for intracellular staining, and blocked with 10% FBS in PBS for 1 h at room temperature in a humidified chamber. Cells were stained with primary antibodies against LC3β (#sc-376404; Santa Cruz Biotechnology), Drp1 (6Z-82#sc-101270; Santa Cruz Biotechnology), and TOMM40 (#18409-1-AP; Proteintech) in 2% FBS-PBS solution. After 3× washes with PBS, cells were incubated with secondary antibodies against rabbit Cy2 (for TOMM40), mouse Cy3 (for Drp1), and mouse Cy5 (for LC3β). Cells were then mounted in Fluromount-G mounting medium (#0100-01; SouthernBiotech) and stored at 4°C until imaging. Fluorescence signals were detected, and images were captured using a Nikon Eclipse Ti inverted confocal microscope with CFI Plan Apochromat Lambda D_100× oil objective (NA 1.45). Images were acquired with a DS-Qi2 camera using NIS-Elements Advanced Research software. Raw image files were then further processed using FIJI/ImageJ software (version 1.54p).

For mitochondrial imaging, stably transduced MitoGFP-expressing HUVEC and U2-OS cells were cultured on coverslips in EBM2 (#CC-3162/6; Lonza) and MyCoy 5A modified (#16600082; Gibco) cell culture medium respectively with 10% FBS at 37°C in 5% CO2 humifified incubator. HUVEC and U2-OS cells were treated with either 2.14 μM of K21, or 100 μM of DFO (#D9533; Merck), or 5 μM of FCCP (#C2920; Sigma-Aldrich) for 12, 24, and 48 h. Upon finishing of timepoint, the cells were washed with 1x PBS (+Mg and Ca_2_) and were processed and imaged, as mentioned above.

Confocal images of U2-OS cells expressing MitoQC were acquired using a Leica Stellaris 8 laser scanning confocal microscope, a 63× oil immersion objective lens (NA 1.4), and a 2× zoom setting, with 587-nm excitation and 610-nm emission used to collect the mCherry component of MitoQC (which was not quenched by the acidic pH of the endolysosomal system), whereas 488-nm excitation and 509-nm emission was used to collect the GFP component of MitoQC (which was quenched by the acidic pH of the endolysosomal system). The resulting images were analyzed in Fiji (v2.16.0/1.54p) using background subtraction (rolling paraboloid = 25), Gaussian Blur (sigma = 2), and Auto Threshold (Li) to select individual cells and remove background. The Image Calculator function was used to generate a 32-bit floating-point ratio image (mCherry channel divided by the GFP channel), where each pixel value represented pH-dependent differences in MitoQC fluorescence. The mitolysosome threshold was set at a cutoff 4 standard deviations above the mean for a Bafilomycin A1 control, and the Analyze Particles function was then used to identify and quantify individual mitolysosomes in each identified cell.

### Immunoblotting

Immunoblotting was carried out as described before (Gulve et al, 2016; Prusty et al, 2018). The following antibodies were used for immunoblotting. LONP1 antibody (#ab224316; Abcam), Mitofilin antibody (#ab245764; Abcam), Miga1 (FAM73A) Antibody (#PA553611; Invitrogen), Drp1 antibody (#sc-271583; Santa Cruz Biotechnology), Parkin antibody (#sc-32282; Santa Cruz Biotechnology), Pex14 antibody (#10594-1-AP; Proteintech), p53 antibody (#sc-126; Santa Cruz Biotechnology), LC3β antibody (#18725-1-AP; Proteintech), Tom20 antibody (#sc-17764; Santa Cruz Biotechnology), and BNIP3L/Nix (D4R4B) Rabbit Monoclonal Antibody (#12396; Cell Signaling). Equal protein loading was confirmed by Vinculin antibody (#sc-73614; Santa Cruz Biotechnology). All the primary antibodies were used at a dilution of 1:200∼1:1,000. HRP-conjugated secondary antibodies, which are Goat Anti-Mouse IgG Antibody, HRP-conjugate (#12-349; Sigma-Aldrich), and Goat Anti-Rabbit IgG Antibody, HRP-conjugate (#12-348; Sigma-Aldrich), were used at a dilution of 1:2,000.

### Cytokine ELISA assays

Secreted IL-12 and IL-10 levels were measured in culture media after 10 d of macrophage differentiation. The supernatants were collected and centrifuged at 10,000*g* for 15 min at 4°C to remove cellular debris. Cleaned supernatants were aliquoted and frozen at −80°C. The following kits were used for cytokine measurements: IL-12 p70 ELISA kit (Cat# 88-7126-88, Lot# 36132-005; Invitrogen, Thermo Fisher Scientific) and IL-10 ELISA Kit (Cat# 88-7106-88, Lot# 399831-005; Invitrogen, Thermo Fisher Scientific). As cytokine levels did not differ statistically on days 2, 3, and 4 post-LPS/IFNγ treatment, the day 4 levels are shown for simplicity. Every set of experiments included a naïve counterpart (i.e., no LPS/IFNγ treatment); no detectable levels of IL-10 or IL-12 were observed in these naïve controls.

### *E. faecalis* culture and infection

*E. faecalis* (OG1RF, pMV 158:gfp) bacteria were inoculated in 1.5 ml of Brain Heart Infusion (BHI) broth supplemented with 10 μg/ml tetracycline and incubated overnight at 37°C. After incubation, 1 ml of bacterial culture was centrifuged at 1,000*g* for 5 min. The pellet was washed two times with 1 ml of PBS. After washing, bacteria were resuspended in 1 ml of PBS with a final concentration of 3 × 10^8^ cells/ml. Serial dilution experiments were carried out, and CFU counts were performed on the overnight culture to determine the bacterial concentration.

Monocytes were treated with K21 (0.28 μM) from day 0. After 10 d of monocyte-to-macrophage differentiation, cells were treated with either FCCP (10 μM) or DFO (100 μM), or wortmannin (10 nM), or hydroxychloroquine (1 μM), for 6 h. After the treatment, macrophages were washed thoroughly to remove traces of K21 from the culture media and were infected with *E. faecalis* at an MOI of 100 for 2 h. After infection, the cells were washed three times with PBS and incubated in media supplemented with Gentamicin (200 μg/ml) for another 2 h. Finally, cells were washed three times with PBS, and DNA was isolated using the Qiagen DNeasy DNA isolation kit (69506).

Genomic qPCR was performed with TaqMan primers for *E. faecalis* genes efaA (APEP3TP) and fsrC (APKCE3E), and Actin B as a housekeeping gene, using Maxima Probe qPCR Master Mix (K0263). For the gentamicin protection assay, macrophages were prepared as before, with the only treatment being FCCP (10 μM) for 6 h. After the final wash, cells were lysed with 1 ml of cold distilled water and incubated for 15 min at 4°C; 10-fold serial dilutions were prepared from the lysate, and 10 μl of the 10:1, 10:2, and 10:3 dilutions were plated on BHI agar plates in triplicate, then incubated overnight at 37°C. After incubation, colonies were counted, from which CFU/ml was calculated.

### ATP measurement

Intracellular ATP levels were quantified using the ATP Determination Kit (A22066; Invitrogen) following the manufacturer’s instructions. ATP content was normalized to total protein amount, as determined by the Pierce Detergent Compatible Bradford Assay Kit (23246; Thermo Fisher Scientific).

### MitoTimer transfection in HUVEC cells

HUVEC cells were cultured on 18-mm glass coverslips in EGM-2 growth medium (#CC-3162/6; Lonza), supplemented with EGM-2 SingleQuots (#CC-4176; Lonza). Cells were transfected with 1 μg of pMitoTimer plasmid (Addgene; plasmid#52659) using the 4D nucleofection system (#AAF-1003X; Lonza) and reagents for primary cell transfection (#V4XP-5032; Lonza) according to the manufacturer’s protocol. After transfection, cells were incubated for 24 h at 37°C with 5% CO_2_. Successful transfection was validated by observing cells under a fluorescence microscope for green and red mitochondrial signals. Transfected cells were then treated with either 2.14 μM of K21, or 100 μM of DFO (#D9533; Merck), or 5 μM of FCCP (#C2920; Sigma-Aldrich) for 12, 24, and 48 h. After reaching the timepoint, cells were washed with PBS (+Mg and +Ca_2_), fixed with 4% formaldehyde, and mounted for microscopy using Fluromount-G (#0100-01; SouthernBiotech) mounting medium. Confocal microscopy was performed on a Nikon Eclipse Ti inverted confocal microscope, and images were captured with a 100× oil objective for both green (excitation/emission 488/518) and red (excitation/emission 543/572) channels. Raw image files were analyzed using FIJI software.

### Preparation of K21 for *C. elegans* experiments

A 50% stock solution of K21 QASD in ethanol (kindly provided by FiteBac LLC) was used to create a working stock colloidal suspension of 0.2% K21 in 0.8% ethanol. A 0.8% ethanol solution without K21 was used as a vehicle-only control. For treatment, agar plates seeded with OP50 were dosed with 150 μl of either K21 or vehicle, and the plates were rotated to ensure the entire OP50-containing surface was covered with either substance. Plates were then left to dry for approximately 20 min, allowing the liquid to be absorbed into the agar surface. Embryos harvested after bleach isolation were then deposited on the plates and allowed to hatch. Hatched animals were typically assessed at the L4 stage unless otherwise noted.

### Strains and transgenic animals

*C. elegans* strains were derived from the N2 strain, with hermaphrodites analyzed in all experiments. Strains, including *lgg-1(tm3489)*, *drp-1(tm1108)*, *atfs-1(cmh15)*, *eat-2(ad1116)*, *eat-3(ad426)*, *wwEx53[P*_*acdh-2*_*::gfp]*, *bvIs5[P*_*cyp-35B1*_*::gfp, P*_*gcy-7*_*::gfp]*, and *zcIs13[P*_*hsp-6*_*::gfp]*, were obtained from the *C. elegans* Genetics Center. The strain PHX3848 expressing VDAC-1::mNeonGreen from the endogenous *vdac-1(syb3848[vdac-1::mNeonGreen::3xFLAG])* was generated by SunyBiotech using CRISPR/Cas9 editing. Nematodes were cultured on OP50 *E. coli* seeded on NGM plates unless otherwise stated.

The *C. elegans* version of the mitochondrial Keima (*MitoKeima*) sequence was synthesized by GenScript Biotech Corporation using a codon-optimized sequence derived from the mammalian mKeima gene that was modified to reflect the preferred codon usage of *C. elegans* while preserving the original amino acid sequence. To generate the construct *P*_*vha-6*_*::MitoKeima* for expression of MitoKeima in the intestine of *C. elegans*, a Gateway LR recombination reaction was performed using the donor vector (*pUC57_Mito-mKeimaWormattL*) and the destination vector (*pPS2_pCFJ150_vha-6_GTWY*), which contains the *vha-6* promoter. The destination vector was kindly provided by Barth Grant (Rutgers University, NJ, USA). Transgenic strains were generated by microinjecting the *P*_*vha-6*_*::MitoKeima* plasmid (25 μg/μl) along with the co-injection marker *P*_*ttx-3*_*::rfp* (a gift from Oliver Hobert, Columbia Univ.) into the germline of the wild-type N2 strain. To obtain integrated lines, X-ray irradiation was applied to six plates (each containing ∼25 worms on a 6 cm NGM plate), followed by standard protocols for isolating stable integrants. The resulting integrated strain, called *odIs167*, was backcrossed to N2 six times before use in experiments.

### Fluorescence microscopy and image analysis

Transgenic strains were grown at 20°C, allowed to lay eggs overnight, and then removed from the plate. K21, vehicle, or ethidium bromide (for examining *P*_*hsp-6*_*::gfp* expression) were added to these plates, which were then monitored until hatched nematodes reached L4 stage. Fluorescent proteins were visualized in nematodes by mounting on 2% agarose pads with 10 mM levamisole.

For most MitoKeima analyses, *odIs167[P*_*vha-6*_*::MitoKeima]* transgenic animals were examined via confocal imaging using a spinning disk confocal microscope equipped with a 100× Zeiss Plan-APOCHROMAT oil immersion objective (1.4 NA; Carl Zeiss Microscopy), a CREST OPTICS X-Light V2TP Confocal Imager (BioVision Technologies), an 89NORTH LDI laser illumination system (89 North), and a PRIME 95B sCMOS camera (Teledyne Photometrics). Dual-excitation imaging of MitoKeima was achieved using 470-nm and 555-nm excitation wavelengths, with a single emission collected at 620 nm. Mitochondrial morphology and intensity analysis were performed based on the ImageJ macro protocol described by Valente et al (2017), including rolling ball background subtraction (50-pixel paraboloid), unsharpening mask filtering (2 pixels, weighted 0.6), CLAHE (block size 127, 256 bins, maximum slope of 3.0), and median filtering and despeckle filtering to reduce pixel noise (2.0 pixels). Regions of interest (ROI) and signal thresholding were used to direct the quantification of mean pixel intensity from each of the dual channels. For each identified mitochondrion, the ratio of mean pixel intensity emitted at 620-nm from 555-nm excitation (mitophagic) versus 470-nm excitation (non-mitophagic) was calculated and plotted.

For analysis of MitoKeima colocalization with VDAC-1::mNeonGreen, *odIs167[P*_*vha-6*_*::MitoKeima]* transgenic animals were introduced into animals with *vdac-1(syb3848[vdac-1::mNeonGreen::3xFLAG])*, then mounted on slides in BIO-133 (MY Polymers Ltd.) biocompatible polymer followed by a 30-s crosslinking with a 365-nm UV light to cure the polymer between the slide and coverslip. Super-resolution imaging was performed on a ZEISS ELYRA 7 with Lattice SIM microscope equipped with an sCMOS camera and a 63X oil immersion (NA 1.4 with RI correction) objective to detect fluorescence. Fluorophores were sequentially excited with diode lasers (i.e., 488 nm for VDAC-1::mNeonGreen and the neutral version of MitoKeima; 561 nm for the acidic version of MitoKeima) using an LBF 405/488/561/642 main beam splitter. Fluorescence emission was separated with a secondary beamsplitter to direct MitoKeima emission (from both excitation wavelengths) to camera 1 (BP 570–620 nm and LP 650 nm) and VDAC-1::mNeonGreen emission to camera 2 (BP 495–550 nm). Three-dimensional stacks were acquired using nine phases of lattice illumination followed by reconstruction using ZEN Black SIM^2^ software (Zeiss) with the strong fixed processing preset. Single representative planes from the stacks were chosen to visualize colocalization.

Triple labeling experiments to detect mitochondria or lysosomes in nematodes expressing MitoKeima were performed with commercial dyes. To detect mitochondria, transgenic nematodes were stained by incubating them on NGM plates seeded with OP50 and supplemented with 4 μM MitoTracker Green FM (Cat.#: M7514; InvitrogenTM by Thermo Fisher Scientific) for 24 h. To detect lysosomes, transgenic nematodes were stained with 2 μM LysoTracker Green DND-26 (Cat.#: L7526; InvitrogenTM by Thermo Fisher Scientific) in M9 buffer, incubating them in the dark for 6 h. After staining, nematodes were transferred to standard NGM plates for 1 h in the dark to allow excess dye to clear before imaging.

Starvation was used as a positive control for inducing mitophagy in the intestine. Late L4-stage *odIs167* nematodes expressing MitoKeima were first transferred to an unseeded (OP50-free) NGM plate for 1 h to allow them to cast off the food coating their cuticle, then moved to a different unseeded plate and maintained there for 24 h before imaging.

For quantification of GFP-expressing strains, including *wwEx53[P*_*acdh-2*_*::gfp]*, *bvIs5[P*_*cyp-35B1*_*::gfp]*, and *zcIs13[P*_*hsp-6*_*::gfp]* transgenic strains*,* nematodes were mounted on agarose pads as described above, and the resulting slides were visualized on an AxioImager M1m (Carl Zeiss). A 5× (NA 0.15) or 10× (NA 0.30) PlanApo objective was used to detect fluorescence. Images were acquired with an ORCA charge-coupled device camera (Hamamatsu) using iVision software v4.1 (BioVision Technologies). Exposure times were chosen to capture at least 95% of the dynamic range of fluorescent intensity of all samples. Quantification was performed by obtaining outlines of nematodes using transmitted light images. The mean fluorescence intensity within each outline was calculated after subtracting away background coverslip fluorescence using a rolling ball filter in Fiji/ImageJ 2.1.0/1.53c55. All data with normal distributions were analyzed with GraphPad Prism 9.3.0 in most cases using ANOVA with Dunnett’s post hoc test correction for multiple comparisons.

### Lifespan analysis

*C. elegans* strains were grown at 20°C on food for at least two generations before the experiments. For synchronization, 20–30 gravid, well-fed adults were transferred to a new NGM plate with live *E. coli* OP50-1 bacteria to lay eggs for 4–5 h, and then removed. Progeny hermaphrodite animals were maintained at 20°C until the late L4 larval stage, and lifespan assays were counted as days after the L4 stage. Animals were transferred away from their progeny to fresh OP50-seeded plates every 1–2 d until the end of their reproductive period. Survival analyses were performed using the Kaplan–Meier method, and the significance of differences between survival curves calculated using the log-rank test. Animals that showed defects due to aberrant vulval development or egg laying (e.g., bursting at the vulva and bagging) or desiccated on the side of the dish were censored at the time of their demise. Three independent biological replicates were performed; all three gave the same mean lifespan.

### OP50 titration experiments

Bacterial lawns of OP50 grown on NGM plates were exposed to K21 or vehicle using the same protocol used for *C. elegans* exposure. After 3 d, bacteria were harvested by adding 1.6 ml of sterile M9 buffer to the plates and gently scraping to collect the remaining bacteria. Harvested bacteria were diluted in LB broth over multiple 10-fold serial dilutions, plated on LB plates, and counted for colonies after an overnight incubation at 37^o^C. Each condition was performed in triplicate.

### Pharyngeal pumping analysis

Nematodes were allowed to grow on OP50 lacking drug until L4, when 5–10 were transferred to test plates (either K21 or vehicle) and allowed to acclimate at 20^o^C for 30 min. These plates were then placed under a dissection microscope and recorded using StreamPix SingleCam 10.1.0.0 (x64) under normal monochrome brightfield conditions. Each worm was recorded for approximately 20 s, and each video was then observed at 0.25× speed, with each pharyngeal pump recorded on a counting app. This number was then used to calculate the rate per minute for each worm, and then the averages were taken of each condition.

### OP50 plate clearance analysis

Feeding behavior was assessed by measuring the optical density at 600 nm (OD_600_) of *E. coli* OP50 cultures using a Tecan Infinite M200 Pro plate reader with the Tecan i-control software. Greiner 96-well flat-bottom transparent plates were used for analysis. Bacterial lawns of OP50 grown on NGM plates were harvested by adding 1.6 ml of sterile M9 buffer to the plates and gently scraping to collect the remaining bacteria. Under these conditions, OD_600_ values for control and K21-treated plates without nematodes were approximately 1, whereas the blank M9 buffer had an OD_600_ value of approximately 0.04. For OD_600_ measurements, 300 μl of the sample was loaded per well, followed by 12 s of linear shaking at 1.5 mm amplitude before readings. Each well was measured using 20 flashes with triplicate reads (3 × 3 points per well), and the average value was recorded for each sample. A standard curve was generated using serial dilutions of harvested bacteria (concentrations: 1, 0.75, 0.5, 0.25, 0.125, and 0), prepared 24 h beforeo the experiment, and OD_600_ values were recorded for each dilution. After generation of the standard curve, 200 hand-picked young adult stage *C. elegans* N2 and *eat-2(ad1116)* animals, grown from eggs under identical conditions, were added to both control and K21-treated plates. Food consumption rates were assessed by measuring the OD_600_ of the remaining bacteria 24 h after introducing the worms. Each condition was performed in triplicate.

### *C. elegans* growth and development assays

All genotypes were synchronized by alkaline bleaching and arrested at the L1 stage overnight in M9 buffer. Synchronized genotypes were assayed 43–46 h after reaching the L4 stage, when embryos present within adult animals were counted using a dissection microscope. From 40 to 60 animals were analyzed and pooled from three biological replicates. Averages represent unlaid eggs per animal.

### Bulk RNA-seq of *C. elegans*

Developmentally synchronized animals were obtained by hypochlorite treatment of gravid adults and embryos hatched overnight for 15–17 h in M9. Starvation-arrested L1s were plated on NGM plates and grown at 20°C until the L4 stage. Total RNA was isolated from animals using TRIzol (Invitrogen) combined with Bead Beater lysis in five biological replicates for each genotype. RIN values for the RNA ranged from 9.4 to 9.9. An mRNA library (paired-end, 150-bp reads) was prepared for each sample/replicate using Illumina TruSeq with PolyA selection (Azenta/GeneWiz). Libraries were sequenced across two lanes on an Illumina HiSeq2500 (Azenta/GeneWiz), resulting in a range of 27–37 M reads per sample, with peak mean Phred scores of 38.76. Reads were mapped to the *C. elegans* genome (WS245) and gene counts generated with STAR 2.5.1a. Normalization and statistical analysis of gene counts were performed with EdgeR using generalized linear model functionality and tagwise dispersion estimates. Multidimensional scaling analysis showed tight clustering within five biological replicates, with a clear separation between treatments. Likelihood ratio tests were conducted in a pairwise fashion between genotypes with a Benjamini–Hochberg correction. Genes were considered to be differentially regulated if they were differentially expressed with an FDR<0.01 in the same direction (up or down). RNA-seq data sets are available at NIH/NCBI GEO through accession number GSE296571.

### GO and GSEA

For GO enrichment, three separate sets of differentially expressed genes from the RNA-seq analysis were analyzed: “UP” genes, which were genes showing elevated expression after K21; “DOWN” genes, which were genes showing depressed expression after K21; and “ALL” genes, which were a combination of the UP and DOWN genes. GO enrichment was performed using two separate approaches. WormCat 2.0 (http://www.wormcat.com) and gProfiler annotational clustering (https://biit.cs.ut.ee/gprofiler/gost) (Holdorf et al, 2020; Kolberg et al, 2023).

To test enrichment of user-defined gene lists, GSEA was performed on DEGs from the RNA-seq data. GSEA version 4.3.3 [build:16] was used with the preranked RNA-seq data to determine whether there was enrichment of specific stress response pathways, as described by Soo et al (2021). These experiments were run using the preranked version of the software, with the default permutation count of 1,000.

### Orthology analysis

Differentially expressed genes (FDR≤0.05) induced by K21 treatment in the macrophage and monocyte populations, as identified in the scRNA-seq, were used as queries in Ortholist2 (http://ortholist.shaye-lab.org) to identify putative orthologs based on matches in at least two Ortholist2 databases. Identified *C. elegans* orthologs were then cross-referenced with a strict threshold (FDR≤0.001) of DEGs from the *C. elegans* RNA-seq induced by K21 treatment. Log2 fold changes from the PBMC scRNA-seq and *C. elegans* RNA-seq for each *C. elegans*/Human ortholog combination were then visualized in a heat map and organized based on presence or absence in overlapping datasets.

### Statistical analysis

Simple calculations were done in MS Excel v16.64 and using GraphPad Prism 10.0. Data for phenotypic analysis (e.g., survival, lifespan, and fertility assays, and individual-animal fluorescence measurements) were analyzed using GraphPad Prism 9.3.0. Larger datasets (e.g., RNA-seq) were analyzed with scripts written in R or as otherwise indicated above. Where possible, researchers were blind to genotype or experimental treatment. Statistical tests were as indicated in the figures; two-sided tests were used unless otherwise indicated. Data were tested for normality using the Kolmogorov–Smirnov test and adjusted for multiple comparisons, as indicated in the figure legends.

Error bars displayed on graphs represent the means ± SD of three or more independent replicates of an experiment unless otherwise stated. Statistical significance was calculated separately for each experiment and is described within individual figure legends. For image analysis, six or more biological replicates per sample condition were used to generate the represented data. The results were considered significant at *P* ≤ 0.05. The distribution of cells into clusters based on the presence or absence of K21 pretreatment was subjected to binomial analysis comparing observed cell numbers to expected cell numbers, assuming an equal binomial distribution between the two pretreatment options. The resulting *P*-value was adjusted for multiple comparisons and family-wise error using a Bonferroni correction.

## Supplementary Material

Reviewer comments

## Data Availability

Further information and requests for resources and reagents should be directed to Bhupesh K Prusty (bhupesh.prusty@rsu.lv) and Christopher Rongo (crongo@waksman.rutgers.edu). Files for the HMDM scRNA-seq data sets are available at the NIH/NCBI GEO under accession number GSE301383, and the same for PBMC scSLAM-seq data GSE301385. Files for the *C. elegans* RNA-seq data sets are available at the NIH/NCBI GEO under accession number GSE296571. Files can be directly accessed at the web link https://www.ncbi.nlm.nih.gov/geo/query/acc.cgi?acc=GSE296571. The files GSM8972749, GSM8972750, GSM8972751, GSM8972752, and GSM8972753 contain data for five independent biological replicates for N2 wild-type nematodes treated with K21. The files GSM8972744, GSM8972745, GSM8972746, GSM8972747, and GSM8972748 contain data for five independent biological replicates for N2 wild-type nematodes treated with vehicle alone. Other source data are provided online as a single Source Data file with this article.
