# Supplementary material for: Drug-induced metabolic remodeling promotes antimicrobial activity in macrophages and extends longevity in vivo
Source: Life Sci Alliance. 2026 Sep 17;9(12):e202603852. doi: 10.26508/lsa.202603852 (PMC13586677; doi:10.26508/lsa.202603852)
Supplement: Supplementary file 1 [file LSA-2026-03852_review_history.pdf]

Drug-induced metabolic remodeling promotes antimicrobial activity in macrophages and extends longevity in vivo

Claudia Hollmann, Eun Chan Park, Zheng Liu, Faye Nourollahi, Emils Bašēns, Jonathan Dietz, Trushnal Waghmare, Mehul Vora, Georgy Nikolayshvili, Nanci Kane, Tongbin Li, Fabian Imdahl, Kay Macleod, Zaiga Nora-Krukle, Mohamed Elashiry, Christopher Rongo, and Bhupesh Prusty
DOI: https://doi.org/10.26508/lsa.202603852

Corresponding author(s): Bhupesh Prusty, Riga Stradins University and Christopher Rongo, Waksman Institute/Rutgers University

Review Timeline:		
	Submission Date:	2026-07-17
	Editorial Decision:	2026-08-20
	Revision Received:	2026-08-27
	Accepted:	2026-09-01

Scientific Editor: Tim Fessenden

Transaction Report:

(Note: With the exception of the correction of typographical or spelling errors that could be a source of ambiguity, letters and reports are not edited. The original formatting of letters and referee reports may not be reflected in this compilation.)

Please note that the manuscript was reviewed at *Review Commons* and these reports were taken into account in the decision-making process at *Life Science Alliance.*

Reviews

Review #1

Evidence, reproducibility and clarity (Required)

K21 is a silane derivative previously reported to be effective against viral, bacterial, and fungal infections. In this study, the authors investigated its effects on macrophage differentiation and activation. They found that K21 alters cytokine secretion and cell surface protein expression, selectively promoting the growth of pro-inflammatory macrophages. Additionally, K21 enhances mitochondrial health in peripheral blood mononuclear cells by stimulating mitochondrial recycling through mitophagy. Finally, K21 induces mitophagy and extends lifespan in C. elegans.

Major concerns:

1) The authors repeatedly refer to the "effective broad-range antimicrobial effect" of K21, including in the title. This wording is somewhat misleading, as the study does not include experiments directly linking the reported findings, such as K21's effects on macrophage responses and mitochondrial health to antimicrobial activity. I suggest that the authors tone down this claim or conduct additional experiments to substantiate these connections.

2) The experimental rationale for co-culturing macrophages with HFF or immune cells, as shown in Fig. 1, is not clearly explained. Instead of this artificial experimental setup, I suggest examining the effects of K21 on cell differentiation and gene expression in vivo, for example, by performing single-cell RNA sequencing on tissues from mice treated or untreated with K21.

Significance (Required)

Technically, the study is well executed. The second part of the manuscript, which examines the effects of K21 on mitophagy and lifespan, is particularly significant. However, the connection between the first part (K21's effects on macrophage differentiation and activation) and the second part is weak. This lack of a clear mechanistic link makes the overall narrative somewhat descriptive. I suggest that they focus their message on the effects of K21 on mitochondrial health and tissue homeostasis or regeneration.

How much time do you estimate the authors will need to complete the suggested revisions:

Estimated time to Complete Revisions (Required)

(Decision Recommendation)

Between 1 and 3 months

***Review Commons* values the work of reviewers and encourages them to get credit for their work. Select 'Yes' below to register your reviewing activity at Web of Science Reviewer Recognition Service (formerly Publons); note that the content of your review will not be visible on Web of Science.**

Web of Science Reviewer Recognition

Yes

Review #2

Evidence, reproducibility and clarity (Required)

Summary:

In this study entitled 'Drug-induced metabolic remodeling of immune cell repertoire generates an effective broad-range antimicrobial effect' Hollmann et al. investigate how the silane derivative K21, previously described as a broad-spectrum antimicrobial, modulates innate immune cell metabolism. Using human peripheral blood mononuclear cells (PBMCs), the authors report that K21 induces a subtle remodelling of macrophage repertoire without a complete M1/M2 polarisation shift, promotes mitochondrial fission and mitophagy, and increases expression of metabolic stress-response genes. They further show that C. elegans exposed to K21 show increased mitophagy and lifespan, suggesting an evolutionary conservation of the mechanism. The authors conclude that K21 exerts its antimicrobial effect by remodelling host metabolism and mitochondrial quality control, leading to an impairment of the innate

immune responses.
While the dataset is extensive and the topic is of high interest, the causal link between K21-induced metabolic remodelling is largely correlative and mechanistic depth is required. The text lacks clarity and figures, data presentation, and methodological details are often confusing or incomplete, making the manuscript difficult to follow.

Major comments:

1. The manuscript is very difficult to follow due to unclear data descriptions, missing panels, and inconsistencies between text and figures. For example:

a. Extended Figure 1a and Figure 1a appear identical.

b. Figure 1b, Figure 3f-g are not mentioned in the text.

c. Some datasets referenced in the text are missing (e.g. M2c macrophages in Figure 2c, HUVECs and U2-OS cells in Figure 3, Pex14 mentioned but data not shown, CCCP treatments in Extended Figure 12-13, Pgcy-7::GFP...)

d. M2a in Extended f Figure 1e and Figure 2c is mentioned several times in the graphs.

e. Figure legends are often too minimal to interpret the panels (e.g. Extended Figure 8)

f. Several panels are too small to read.
Altogether, these issues significantly hinder readability and comprehension.
2. The central claim that K21 antimicrobial activity is mediated through macrophage metabolic and mitochondrial remodelling is not convincingly demonstrated. The data presented don't show direct evidence of enhanced antimicrobial activity in K21- treated macrophages. Functional infection assays (co-culture of macrophages treated with K21, with bacteria (fungi or viruses)) should be done to establish the link between remodelling and antimicrobial effect.
3. The evidence for mitophagy mostly relies on LC3 staining, which is insufficient. Other markers of mitophagy have not been assessed (such as PINK1, Parkin, BNIP3L/Nix, P62). Moreover, LC3 imaging was performed only in HUVECs, rather than in the main experimental models (HFFs or macrophages), raising questions about cell-type relevance.
4. DRP1 localisation and activation were not evaluated. The authors interpret the process as 'DRP1-dependent'. DRP1 localisation, recruitment or DRP1 phosphorylation states (Ser616 or Ser637) were not analysed to confirm activation. Additional markers of mitochondrial fusion/fission should also be assessed. These gaps make the claim of DRP1-dependent mitophagy premature and largely speculative.
5. Mitochondrial metabolism is discussed extensively but not directly measured. Techniques such as Seahorse metabolic flux analysis, ATP measurement, flow cytometry would probably strengthen the claims. Mitochondrial length should be quantified rather than area, which is less informative.
6. The intracellular localisation or the cellular uptake of K21 are not demonstrated in the manuscript. Imaging or biochemical evidence of intracellular accumulation would be important for mechanistic interpretation.
7. Replicate numbers, biological variability, and donor information are inconsistently presented. Statistical analyses should be well annotated across figures.
Essential experiments to do:
- Functional infection assays to test antimicrobial activity.
- Mitophagy pathway validation.
- K21 uptake/localisation.
- Direct metabolic analysis.
These are feasible and would significantly strengthen the manuscript.

Minor comments:

1. Improve figure legends by clearly identifying sample type, treatment duration, number of replicates, and statistical tests.
2. Ensure all panels are mentioned and appear in the manuscript.
3. Increase panel size for readability.

4. Justify the use of HUVECs instead of macrophages or HFFs for LC3 imaging, or replicate staining in the relevant immune cells.

Significance (Required)

The study investigates a promising concept in host-directed antimicrobial therapy based on metabolic reprogramming. If validated, these findings could provide a conceptual bridge between immunometabolism, mitochondrial quality control, and host-directed antimicrobial therapy.
However, in its current form, the conceptual advance remains preliminary. The data are interesting but insufficient to support the proposed mechanistic and functional conclusions. The manuscript needs improved presentation, correction of inconsistencies, and additional validation experiments would greatly enhance the manuscript's impact and credibility.

This study will primarily interest researchers working on immunometabolism, mitochondrial metabolism, host-pathogen interactions and antimicrobial therapies.

Reviewer expertise:

Immunometabolism, macrophage biology with a focus on mitochondrial metabolism, function and dynamics in kidney and mitochondrial diseases. Competent to evaluate cellular and molecular aspects of the study, but not an expert on C. elegans analyses.

How much time do you estimate the authors will need to complete the suggested revisions:

Estimated time to Complete Revisions (Required)

(Decision Recommendation)

Between 3 and 6 months

***Review Commons* values the work of reviewers and encourages them to get credit for their work. Select 'Yes' below to register your reviewing activity at [Web of Science Reviewer Recognition Service] (formerly Publons); note that the content of your review will not be visible on Web of Science.**

Web of Science Reviewer Recognition

Yes

Review #3

Evidence, reproducibility and clarity (Required)

Summary

Hollmann and Park et al, have investigated the effects of broad range antimicrobial K21 on macrophage differentiation via a combined approach of surface staining and single cell RNAseq. Having identified that mitochondrial fission and mitophagy occurs following K21 treatment, they investigated the effects of K21 on C. elegans biology, further identifying its role in promoting mitophagy and extending the life span of this model organism.

Major comments

1. M1 and M2 is status is defined by LPS+IFNg and IL-4 treatment respectively. GM-CSF-derived HMDM and CSF-1derived are not often labelled "M1" and "M2" and it would be more appropriate to label them "M0 GM-CSF-derived HMDM" and "M0 CSF-1-derived HMDM". Your writing and labelling should be updated to fit this convention.
2. The papers premise is that K21 reprograms immune cell metabolism to enhance antimicrobial activity. It's important to verify that you observed changes in immune cell phenotype is accompanied by enhanced antimicrobial capacity. Subjecting your macrophages (both purer CD14+ and your HFF supplemented HMDM) to a gentamicin exclusion assay/infection CFU assay with pathogenic and non-pathogenic bacteria in the presence or absence of K21 would bolster all your findings. Due to direct antimicrobial nature of K21, you should consider removing it from media just prior to infection.
3. OPTIONAL: Figure 1A-B. IL12 and IL-10 cytokine release would occur rapidly following your LPS/IFNg treatments, peaking by 24-48 hours. The 4-day time point could be confounded by cytokine degradation or reuptake by the

macrophages. Earlier time points might capture the greatest level of cytokine release and maybe capture the greatest effect size of K21 treatment. Also, IL-12 and IL-10 are both induced by LPS stimulation and you lack a "unstimulated" population in which they only receive GM-CSF or M-CSF-1.
4. Supplement mitochondrial fission microscopy data with DRP1 phosphorylation as a direct marker of DRP1 activation and fission via K21. (phosphorylated DRP1 S616 should suffice)
5. Does K21 influence immune cell mitochondrial dynamics? It would be worth examining naïve m-CSF1-derived HMDM and perhaps T- cells to determine if they also have K21-inducible mitochondrial fission and mitophagy. This can be done using mitotracker staining or via IF staining against Tom20.

Minor comments

1. Definition of HFFs needs to occur in first results paragraph as opposed to page 9
2. I appreciate the consistent Y axis scaling in Figure 1D to compare your macrophage sub populations, but it is difficult see and interpret the effects sizes like the one you're describing the activated M1 population. Can you either decrease the Y axis range or put a break in the Y axis of this panel?
3. Some of the effects on protein expression in Figure 3D are not every clear. It would be appropriate to quantify band intensity normalised to your housekeeping protein control for each assessed protein.
4. Can you make dedicated method sections for "HMDM generation (please include your CD14 enrichment vs total monocyte approach) and HMDM polarisation (please include your methodology on HFF culture and co-culture with HMDM).
5. The method section is two method sections put one after the other, presumably form the two labs. Please integrate your two methods sections and remove duplicate sections (statistics and data availability).
6. Please provide references for description of GBP1, SOD2 and GBP5 protein functions on page 8
7. In Extended data figure 13, can you have an image of your MitoKeima colocalising with mitotracker green in starved C. elegans? If the dye no longer works within the lysosome, perhaps IF detection of a mitochondrial protein?
8. eat-2 line not described in the results section at all and extended data figure 16C-D aren't referenced at all?
9. "Despite K21-treated animals showing similar feeding compared to untreated animals (Figure 4F)" I think this refers to Extended Figure 16 and I think the statement is at odds with your observed increase with pharyngeal pumping. Can this be discussed and explained?
10. In text referencing for Extended Figures 11 and onwards lack reference to specific figure panels.
11. Statistical analyses in Figure 1B&D, Extended Figure 1B, Extended Figure 2A&C, should be performed using two-way ANOVA with a post hoc statistical test as opposed to unpair T-tests.
12. Statistical analysis in Figure 3C should be performed using a one-way ANOVA with a post hoc statistical test.

Significance (Required)

The paper significance is drawn from the findings that K21 influences macrophage cell identity and presumably function. The finding that K21 influences mitochondrial dynamics and promotes mitophagy was identified in cell lines and in vivo using C. elegans. There is however a disconnect as there is no functional data that K21 is boosting macrophage antimicrobial function or if the effect on mitochondria contributes to observed chances in macrophage polarisation. The observed effects on longevity in C. elegans are interesting but quite disconnected from immunological scope at the start of the paper. A lot of the previous work into K21 relates to dentistry and wound healing, so this paper provides development into innate immunology and some conceptual advances in its role in immune cell regulation. Audiences interested in this paper would be split between immunologists interested in developing host directed therapies and mitochondrial/mitophagy biologists interested in longevity and mitochondrial health/maintenance. As a reviewer, my research experience is in macrophage biology pertaining to inflammatory and antimicrobial responses downstream of metabolic rewiring. I have some experience collaborating with a C. elegans laboratory but my input to that section of this manuscript is limited.

How much time do you estimate the authors will need to complete the suggested revisions:

Estimated time to Complete Revisions (Required)

(Decision Recommendation)

Between 3 and 6 months

***Review Commons* values the work of reviewers and encourages them to get credit for their work. Select 'Yes' below to register your reviewing activity at Web of Science Reviewer Recognition Service (formerly Publons); note that the content of your review will not be visible on Web of Science.**

Web of Science Reviewer Recognition

No

Rebuttal to Reviews
We are grateful that two reviewers found our work "technically well-executed", with the "effects of K21 on mitophagy and lifespan… particularly significant." Both reviewers provided important feedback. We have added additional experiments, expanded our description of the work to clarify key points, and addressed reviewer questions and concerns (written in blue italics) in the following point-by-point rebuttal (authors' response in non-italics).

Reviewer #1
Evidence, reproducibility and clarity
K21 is a silane derivative previously reported to be effective against viral, bacterial, and fungal infections. In this study, the authors investigated its effects on macrophage differentiation and activation. They found that K21 alters cytokine secretion and cell surface protein expression, selectively promoting the growth of pro-inflammatory macrophages. Additionally, K21 enhances mitochondrial health in peripheral blood mononuclear cells by stimulating mitochondrial recycling through mitophagy. Finally, K21 induces mitophagy and extends lifespan in C. elegans.

Major concerns:

1) The authors repeatedly refer to the "effective broad-range antimicrobial effect" of K21, including in the title. This wording is somewhat misleading, as the study does not include experiments directly linking the reported findings, such as K21's effects on macrophage responses and mitochondrial health to antimicrobial activity. I suggest that the authors tone down this claim or conduct additional experiments to substantiate these connections.

Authors' Response: This statement comes largely from the published literature, which we have cited. But the reviewer makes a good point that it is worth re-testing these properties in our specific system, which we have done. We have included new experiments in which we have pre-treated PBMCs with K21, washed out the drug, and then challenged the modified monocytes and macrophages in the culture with Enterococcus faecalis, a common bacteria used to test innate immune response. As expected, K21 pre-treatment robustly boosts the ability of macrophages to fight off the E. faecalis infection. We have added this data at the beginning of the new Figure 2, which launches our description of experiments testing how K21 modifies the functional properties of innate immune cells. We thank the reviewer for this suggestion, which has made the manuscript significantly better.

2) The experimental rationale for co-culturing macrophages with HFF or immune cells, as shown in Fig. 1, is not clearly explained. Instead of this artificial experimental setup, I suggest examining the effects of K21 on cell differentiation and gene expression in vivo, for example, by performing single-cell RNA sequencing on tissues from mice treated or untreated with K21.

Authors' Response: We do note the reviewer's concern regarding the complexity of the co-culture experiments, and we have therefore reduced the complexity of the experiments we presented regarding the co-culture. Our reasoning for examining macrophage differentiation and polarization in different co-cultures with either HFF or immune cells was to show that our PBMC analysis protocol reflected the subtle changes triggered by such conditions in previous publications, thereby validating the ability of our approach to detect subtle changes that might be induced by K21 treatment. We apologize that this rationale was not made clear. We have addressed this point by removing the HFF and immune co-culture experiments performed with K21, as K21 only showed modest differences under the co-culture conditions; this should simplify the reading of the paper. Instead, we have restricted the description of the HFF and immune co-culture conditions to our initial analysis of our PBMC cells

(now Figure 1), and we have re-written the sections describing these experiments to highlight the rationale: validation of our PBMC approach to detect subtle modulatory effects.

We agree that examining cell differentiation in vivo in mice is an excellent long-term goal. However, we thought it appropriate to start with human PBMCs as an important first step, as such an approach minimizes experimental variability compared to the equivalent in vivo experiment in mice. Capturing macrophages from an in vivo wound model would be challenging, as would ensuring that all assayed cells had been equally exposed to K21. By contrast, the PBMC experiment controls for many of these variables. Our hope is that our findings from PBMCs provide a foundation and premise for future in vivo experiments in mammalian wounds, which are currently beyond the scope of this current manuscript given the technical challenges that will need to be solved, as well as the substantial mouse costs required to conduct the experiments with sufficient statistical power to overcome the problems of wound-to-wound variability. Ideally, we think that the porcine model would likely be more suitable for this question, as wound modeling in pig is considered a more accurate reflection of human wound biology than that found in rodent models; such future experiments will build on the foundational knowledge acquired from our current human PBMC experiments.

We also re-emphasize that our manuscript <u>does</u> contain in vivo data. K21 can modify cellular mitochondria independent of cell type (i.e., our mechanistic analysis does not have to be performed solely in innate immune cells), which allowed us to test the effects of K21 in vivo by using the whole-animal model C. elegans. K21 has the same effects on mitochondrial fission and mitophagy in intact living C. elegans that it does on human cultured cells, showing not only that K21 mediates its function both in vivo and in vitro, but that this function is deeply conserved over evolutionary time.

Significance

Technically, the study is well executed. The second part of the manuscript, which examines the effects of K21 on mitophagy and lifespan, is particularly significant. However, the connection between the first part (K21's effects on macrophage differentiation and activation) and the second part is weak. This lack of a clear mechanistic link makes the overall narrative somewhat descriptive. I suggest that they focus their message on the effects of K21 on mitochondrial health and tissue homeostasis or regeneration.

<u>Authors' Response</u>: We appreciate the reviewer's assessment of the technical soundness and significance of the study. We have re-written the manuscript by expanding our discussion on mitochondrial health, including what is known about the links between tissue homeostasis and mitochondrial quality. We have also added some additional experiments (the new Figure 5 and Figure 6, plus Supplementary Figures S3 and S4) to flesh out the changes in mitochondria in response to K21, including comparing K21-induction of mitophagy to induction by DFO treatment and FCCP treatment, demonstrating an increase Drp1/DRP-1 stabilization and mitochondrial recruitment in both human cells and C. elegans cells, the use a pH-based mitophagy reporter (MitoQC) in cultured mammalian cells, and a clear demonstration that mitophagy induction by K21 requires the autophagy receptor LC3β/LGG-1. These experiments are described in more detail in the rebuttal to Reviewer 2 below.

Reviewer #2

Evidence, reproducibility and clarity

Summary:

In this study entitled 'Drug-induced metabolic remodeling of immune cell repertoire generates an effective broad-range antimicrobial effect' Hollmann et al. investigate how the silane derivative K21, previously described as a broad-spectrum antimicrobial, modulates innate immune cell metabolism. Using human peripheral blood mononuclear cells (PBMCs), the authors report that K21 induces a subtle remodelling of macrophage repertoire without a complete M1/M2 polarisation shift, promotes mitochondrial fission and mitophagy, and increases expression of metabolic stress-response genes. They further show that C. elegans exposed to K21 show increased mitophagy and lifespan, suggesting an evolutionary conservation of the mechanism. The authors conclude that K21 exerts its antimicrobial effect by remodelling host metabolism and mitochondrial quality control, leading to an impairment of the innate immune responses.

While the dataset is extensive and the topic is of high interest, the causal link between K21-induced metabolic remodelling is largely correlative and mechanistic depth is required. The text lacks clarity and figures, data presentation, and methodological details are often confusing or incomplete, making the manuscript difficult to follow.

Authors' Response: The reviewer raises reasonable concerns regarding clarity of the figures, text, and methodological details. The original manuscript was written in a short "letter" format framed around 4 central figures, which required the explanatory text and narrative to be abbreviated. Given the complexity of the paper and the concerns raised by both reviewers, we have converted the manuscript into a traditional longer format framed around 7 central figures, allowing us to expand textual explanations of the approach. We have also removed some experiments to maintain the manuscript length from becoming too expansive, as well as to simply the complexity of the narrative.

Major comments:

1. The manuscript is very difficult to follow due to unclear data descriptions, missing panels, and inconsistencies between text and figures. For example:
a. Extended Figure 1a and Figure 1a appear identical.

Authors' Response: We apologize for the confusion. Figure 1a and Extended Figure 1a were the same cartoon summary of the experimental setup. We duplicated this panel for Extended Figure 1a to make it easier for the reader to follow the experiments in that Extended Figure without having to turn back to Figure 1. Given the overall concerns raised by both reviewers, we have brought the bulk of Extended Figure 1 into the main manuscript, making it the new Figure 1. The bulk of the old Figure 1 is now Figure 2. We removed the duplicated cartoon that comprised the old Extended Figure 1a and Figure 1a; it is now a single cartoon figure in the new Figure 1C.

b. Figure 1b, Figure 3f-g are not mentioned in the text.

Authors' Response: Old Figure 1b was mentioned at the top of page 6, line 136. To address the concerns of the paper raised by Reviewer 1 (i.e., unnecessary focus on the HFF and immune cell co-culture experiments), we have removed the graph that comprised the old Figure 1b panel.

Old Figure 3-f-g is now new Figure 5B. We have added a description of this data to the text (page 11, lines 335-338). We apologize for this oversight.

c. Some datasets referenced in the text are missing (e.g. M2c macrophages in Figure 2c, HUVECs and U2-OS cells in Figure 3, Pex14 mentioned but data not shown, CCCP treatments in Extended Figure 12-13, Pgcy-7::GFP...)

Authors' Response: We apologize that some of the clusters in the scRNA-seq analysis were mislabeled in the old Figure 2. We have corrected the naming system for the clusters throughout the manuscript, including adopting a nomenclature for the clusters that includes a letter to indicate whether the cluster belongs to the naïve macrophage set of the activated macrophage set, combined with a number for that cluster in each of those two datasets.

Regarding the old Figure 3 (now the new Figure 5), we have indicated on the figure the specific cell type in question to make it easier to match the description in the text to the panels in the figure.

The reference to Pex14 has been removed from the text, which allows the text to focus more on mitochondria.

CCCP treatment was shown in the original Extended Data Figure 14. This data is now found in the new Supplementary Figure S4, panels I and J. The CCCP experiment is now more carefully described in the text (page 14, lines 436-439).

P_{gcy-7}::GFP (shown in the new Supplementary Figure S7) is simply a transformation marker found in the transgenic reporter for $P_{cyp-35B1}$::GFP. We have added an explanation for this in the text (page 16, line 494; page 62, legend for Supplementary Figure S7).

d. M2a in Extended f Figure 1e and Figure 2c is mentioned several times in the graphs.

Authors' Response: We are not sure about the nature of this specific concern here, but as mentioned above, we have carefully revised the data and manuscript with a new scRNA-seq cluster nomenclature to address mislabeled clusters and make the manuscript easier to understand and follow.

e. Figure legends are often too minimal to interpret the panels (e.g. Extended Figure 8)

Authors' Response: We have expanded the figure legends to make it easier for readers to understand the individual panels. The old Extended Figure 8 has been removed to address the reviewers' concerns that some of the additional data in the manuscript diffused the focus and readability of the work.

f. Several panels are too small to read.
Altogether, these issues significantly hinder readability and comprehension.

Authors' Response: Although we do not know to which panels the reviewer is referring, we have increased the size of panels where we have judged them likely to have been too small to read.

2. The central claim that K21 antimicrobial activity is mediated through macrophage metabolic and mitochondrial remodelling is not convincingly demonstrated. The data presented don't show direct evidence of enhanced antimicrobial activity in K21- treated macrophages. Functional infection assays (co-culture of macrophages treated with K21, with bacteria (fungi or viruses)) should be done to establish the link between remodelling and antimicrobial effect.

Authors' Response: Reviewer 1 also had this concern. In addition to citing the literature supporting this link, we have included new experiments in which we have pre-treated PBMCs with K21, washed out the drug, and then challenged the modified monocytes and macrophages in the culture with Enterococcus faecalis, a common bacteria used to test innate immune response. As expected, K21 pre-treatment robustly boosts the ability of macrophages to fight off the E. faecalis infection. We have added this data at the beginning of the new Figure 2, which launches our description of experiments testing how K21 modifies the functional properties of innate immune cells. We have also examined the effects of several known regulators of mitophagy on the ability of macrophages to fight off the E. faecalis infection, which can be found in Supplementary Figure S3C and D. We thank the reviewer for this suggestion, which has made the manuscript significantly better.

3. The evidence for mitophagy mostly relies on LC3 staining, which is insufficient. Other markers of mitophagy have not been assessed (such as PINK1, Parkin, BNIP3L/Nix, P62). Moreover, LC3 imaging was performed only in HUVECs, rather than in the main experimental models (HFFs or macrophages), raising questions about cell-type relevance.

Authors' Response: HUVECs were chosen because of their easily scorable mitochondrial networks. By contrast, macrophage mitochondrial markers and morphology are rather poor, and transfection into these cells is inefficient and often disruptive to their morphology, polarization phenotype, and function. Nevertheless, given the conservation of K21-induced mitophagy in C. elegans, we do not believe this physiological response is limited to macrophages but rather that most (perhaps all) cell types are induced to undergo mitophagy in response to K21. The MitoKeima marker in C. elegans provides direct evidence of mitophagy by monitoring the actual acidification of mitochondria as they enter the autophagosomal membrane system; consistent with this conclusion, mutations in lgg-1 (the nematode LC3β) block the acidification of MitoKeima-labeled mitochondria undergoing mitophagy, which we have added as new data in the new Figure 6.

To strengthen our case for mitophagy in human cells, we collaborated with the laboratory of Dr. Kay Macleod (University of Chicago), who is an established expert in studying mitophagy. Using a U2-OS cell line that stably expresses the fluorescent mitophagy reporter MitoQC, the Macleod lab showed that K21 treatment induces a similar acidification of mitochondria as seen in the MitoKeima experiments in C. elegans. This acidification is blocked by treatment with Bafilomycin A1, which inhibits the V-ATPase responsible for acidifying the autophagy and endolysosomal pathway. This is now in panel E of the new Figure 5.

To assess other mitophagy pathways, we focused on the two most studied: hypoxia-induced mitophagy, which can be induced with the hypoxia mimetic DFO and operates through the mitophagy receptor BNIP3/NIX, and decoupler-induced mitophagy, which can be induced with FCCP treatment and operates through the PINK1/Parkin system. Using two different cell types, we now clearly show that DFO-induced mitophagy and FCCP-induced mitophagy each have their specific molecular signatures (e.g., DFO causes an upregulation of BNIP3/NIX levels, whereas FCCP treatment causes a wide-spread lipidation of LC3β). It is well established that both mechanisms for mitophagy induction cause wide-spread cellular stress. By contrast, K21 treatment caused neither BNIP3/NIX upregulation nor systemic lipidation of LC3β. Rather, K21 treatment induced the stabilization and recruitment of Drp1/DRP-1 to mitochondria to induce fission in both human and C. elegans cells. This data can be found in Figure 5F and G. Our results suggest that K21 operates through a less stressful mechanism of mitophagy induction than that employed by DFO or FCCP treatment, but additional experiments to determine the specific mechanism go beyond the scope of this work.

4. DRP1 localisation and activation were not evaluated. The authors interpret the process as 'DRP1-dependent'. DRP1 localisation, recruitment or DRP1 phosphorylation states (Ser616 or Ser637) were not analysed to confirm activation. Additional markers of mitochondrial fusion/fission should also be assessed. These gaps make the claim of DRP1-dependent mitophagy premature and largely speculative.

<u>Authors' Response</u>: It is unclear whether this comment refers to the mammalian culture cells or the nematode experiments. For the nematode experiments, we simply observed that K21 induced changes in mitochondrial size and morphology in C. elegans consistent with DRP-1-mediated fission. These changes are absent in drp-1 loss of function mutants. By definition, this observation demonstrates that those changes are DRP-1-dependent. We have now conducted additional experiments with strains in which endogenous DRP-1 is tagged with a fluorescent reporter, and we found that K21 treatment causes the recruitment of DRP-1::GFP to mitochondria. This new data can be found in the new Figure 6a-d.

Regarding the human tissue culture experiments, we note that phosphorylation of Drp1 protein is not the only way in which Drp1 activity is promoted. For example, Drp1 directly binds to cardiolipin, which stabilizes and recruits the protein independent of changing its phosphorylation state. Indeed, Drp1 levels are elevated following K21 treatment, which can be found in the new Figure 5D, as well as part of new data found in Figure 5F and G.

5. Mitochondrial metabolism is discussed extensively but not directly measured. Techniques such as Seahorse metabolic flux analysis, ATP measurement, flow cytometry would probably strengthen the claims. Mitochondrial length should be quantified rather than area, which is less informative.

<u>Authors' Response</u>: We have now quantified mitochondrial fission two ways: maximum length per mitochondria and aspect ratio of each mitochondrion. We have also included higher quality images of the mitochondrial morphology. This new data can be found in new Figure 6G and H.

ATP measurements have been added (currently Supplementary Figure S3E and F). Beyond ATP measurements, our findings regarding metabolism are based on transcriptomics. A full metabolics analysis goes beyond the scope of this already data-dense manuscript. We have softened some of our statements by highlighting that a formal proof of metabolic reprogramming will require such analysis in the future.

6. The intracellular localisation or the cellular uptake of K21 are not demonstrated in the manuscript. Imaging or biochemical evidence of intracellular accumulation would be important for mechanistic interpretation.

Authors' Response: The silicon moiety within K21 makes mass spectrometry analysis of the intact chemical prohibitive. Attachment of fluorescent moieties to the drug will likely reduce efficacy. Tracking the uptake and metabolism of a new pharmacological agent is arguably a publication in and of itself.

7. Replicate numbers, biological variability, and donor information are inconsistently presented. Statistical analyses should be well annotated across figures.

Authors' Response: We have addressed this concern by making sure each figure legend highlights the statistical analysis and replicate number used. PBMC donor information has been provided in the methods.

Essential experiments to do:
- Functional infection assays to test antimicrobial activity.
- Mitophagy pathway validation.
- K21 uptake/localisation.
- Direct metabolic analysis.

These are feasible and would significantly strengthen the manuscript.

Authors' Response: As mentioned above, we have added the functional infection assays to test antimicrobial activity (the new Figure 2A-D) and performed additional mitophagy validation using a combination of Western blotting for mitophagy markers and a U2-OS cell line stabling expressing the mitophagy reporter MitoQC (the new Figure 5). We feel that additional experiments to monitor K21 uptake and localization, as well as to monitor metabolism, go beyond the scope of what already is a data-dense manuscript.

Minor comments:

1. Improve figure legends by clearly identifying sample type, treatment duration, number of replicates, and statistical tests.

Authors' Response: We have ensured that the figures and their legends clearly identify sample type, treatment duration, replicate number, and statistical test.

2. Ensure all panels are mentioned and appear in the manuscript.

Authors' Response: We have ensured that the figure panels are all mentioned in the manuscript.

3. Increase panel size for readability.

Authors' Response: We have ensured that the figure panel sizes have been increased to make them more readable.

4. Justify the use of HUVECs instead of macrophages or HFFs for LC3 imaging, or replicate staining in the relevant immune cells.

Authors' Response: We now show mitochondrial fission and mitophagy experiments in HUVECs, HFFs, U2-OS cells, and C. elegans intestinal cells in vivo.

Significance

Significance:

The study investigates a promising concept in host-directed antimicrobial therapy based on metabolic

reprogramming. If validated, these findings could provide a conceptual bridge between immunometabolism, mitochondrial quality control, and host-directed antimicrobial therapy.
However, in its current form, the conceptual advance remains preliminary. The data are interesting but insufficient to support the proposed mechanistic and functional conclusions. The manuscript needs improved presentation, correction of inconsistencies, and additional validation experiments would greatly enhance the manuscript's impact and credibility.
This study will primarily interest researchers working on immunometabolism, mitochondrial metabolism, host-pathogen interactions and antimicrobial therapies.

Reviewer expertise:

Immunometabolism, macrophage biology with a focus on mitochondrial metabolism, function and dynamics in kidney and mitochondrial diseases. Competent to evaluate cellular and molecular aspects of the study, but not an expert on C. elegans analyses.

Authors' Response: We feel that the expanded description of the experiments in the text combined with the additional experiments performed to address the reviewers' concerns provide the needed support for the proposed mechanistic and functional conclusions. Determining the specific molecular mechanism of a discovered drug (i.e., a pre-existing molecule found to have a new surprising biological activity despite not knowing the mechanism of action) is challenging compared to that of a developed drug, where the drug in question is created with a specific mechanism already in mind. For example, aspirin was first synthesized by Bayer in 1897 and shown to mimic the anti-inflammatory effects of willow bark, but it took another 74 years before John Vane and other researchers determined that its mechanism of action was to inhibit the production of prostaglandins by irreversibly binding and disabling COX enzymes. K21 is such a "found" molecule orphaned from its mechanism. Keeping that perspective in mind, our submitted manuscript is a detailed , thorough, and dense first step towards understanding the mechanism of K21 action, and the conservation of its effects on mitochondrial fission and mitophagy both in vitro and in vivo – across a billion years of evolution – provide strong evidence for its mechanism of action, with enough significant impact on our understanding to merit publication at this stage of research.

Reviewer #3 (Evidence, reproducibility and clarity (Required)):

Summary
Hollmann and Park et al, have investigated the effects of broad range antimicrobial K21 on macrophage differentiation via a combined approach of surface staining and single cell RNAseq. Having identified that mitochondrial fission and mitophagy occurs following K21 treatment, they investigated the effects of K21 on C. elegans biology, further identifying its role in promoting mitophagy and extending the life span of this model organism.

Major comments
1. M1 and M2 is status is defined by LPS+IFNg and IL-4 treatment respectively. GM-CSF-derived HMDM and CSF-1derived are not often labelled "M1" and "M2" and it would be more appropriate to label them "M0 GM-CSF-derived HMDM" and "M0 CSF-1-derived HMDM". Your writing and labelling should be updated to fit this convention.

Authors' Response: We thank the reviewer for these nomenclature-related suggestions, with which we agree. But we feel that the constant use of terms such as M0 GM-CSF-derived HMDM and M0 CSF-1-derived HMDM (or M0 M-CSF-derived HMDM, as we chose M-CSF rather than CSF-1 to refer to Macrophage Colony-Stimulating Factor) will be very confusing to readers and cumbersome to read, particularly when these cells are compared with their activated counterparts. Therefore, we argue that using the term "naïve macrophages" would be easier to follow throughout the text. Indeed, one of the important aspects of our scRNA-seq analysis is the demonstration that naïve and activated macrophages show a greater diversity at the level of single cell gene expression than is currently appreciated or otherwise captured using the current nomenclature.

As a compromise that will assist naïve readers and experts in the field alike, we have now incorporated the reviewer-suggested terminology into the text when the issue is first introduced in the Introduction (page 3 of the new draft, lines 91-93) and the beginning of the Results (page 6 of the new draft, lines 168-170), clarifying for readers how our simplified terms map to established nomenclature. We review this issue again when we summarize our findings in the Discussion section (page 18 of the new draft, lines 546-551).

2. The papers premise is that K21 reprograms immune cell metabolism to enhance antimicrobial activity. It's important to verify that you observed changes in immune cell phenotype is accompanied by enhanced antimicrobial capacity. Subjecting your macrophages (both purer CD14+ and your HFF supplemented HMDM) to a gentamicin exclusion assay/infection CFU assay with pathogenic and non-pathogenic bacteria in the presence or absence of K21 would bolster all your findings. Due to direct antimicrobial nature of K21, you should consider removing it from media just prior to infection.

Authors' Response: We thank the reviewer for these important suggestions. Accordingly, we have now added new sets of experiments using the gentamicin exclusion assay and the CFU assay together with E. faecalis infection. This data can be found in the new Figure 2. We agree with the point raised by the reviewer about removing K21 from culture media before infection. We usually remove K21 from the culture and wash the cells thoroughly 2-3 times before the bacterial infection. This point is now clearly mentioned within the methods text (page 7, line 210, and page 31 line 950 of the new draft).

3. OPTIONAL: Figure 1A-B. IL12 and IL-10 cytokine release would occur rapidly following your LPS/IFNg treatments, peaking by 24-48 hours. The 4-day time point could be confounded by cytokine degradation or reuptake by the macrophages. Earlier time points might capture the greatest level of cytokine release and maybe capture the greatest effect size of K21 treatment. Also, IL-12 and IL-10 are both induced by LPS stimulation and you lack a "unstimulated" population in which they only receive GM-CSF or M-CSF-1.

Authors' Response: We thank the reviewer for these critical comments. We measured cytokine levels on days 2, 3, and 4 of LPS/IFNg treatment and observed no significant differences in the time course of treatment between K21-treated and untreated samples. In fact, the cytokine levels are much cleaner and comparable after day 3-4 of treatment. Therefore, we decided to conduct all experiments after day 4 of LPS treatment.

Every set of experiments also included a naïve counterpart (both M1 and M2 naïve) that had not been treated with LPS. These cells have received either M-CSF or GM-CSF for 10 days. These cells served the purpose of unstimulated cells. However, IL-10 and IL-12 levels were undetectable in these cells, making meaningful representation in the graphs difficult. As the purpose of our experiments was to compare M1 to M2 macrophages, and we did not see any detectable amounts of IL-10 and IL-12 in the absence of LPS, it did not make any sense to show them within the graph, as both M1 and M2 are virtually indistinguishable in terms of their cytokine secretion capabilities. We have added this information to the Methods section (page 30, lines 935-938)

4. Supplement mitochondrial fission microscopy data with DRP1 phosphorylation as a direct marker of DRP1 activation and fission via K21. (phosphorylated DRP1 S616 should suffice)

Authors' Response: We note that phosphorylation of Drp1 protein is not the only way in which Drp1 activity is promoted. For example, Drp1 directly binds to cardiolipin, which stabilizes and recruits the protein independent of changing its phosphorylation state. Moreover, both the Drp1 and phospho-Drp1 alterations during mitophagy are cell-type dependent. For example, both DFO and FCCP treatment reduced Drp1 (and phospho-Drp1) levels in HUVECs (new Figure 5F), whereas these levels were upregulated in U2-OS cells (new Figure 5G). This reduction in Drp1 and phospho-Drp1 may simply reflect the rate of mitochondrial recycling and Drp1 turnover. Hence, it is difficult to assess Drp1 levels in cells and their relevance to mitophagy.

As K21 did not induce a drastic upregulation of Drp1 levels, we did not observe a substantial difference in Drp1 and phospho-Drp1 staining by microscopy in human cultured cells. However, we were able to use super-resolution microscopy to examine the C. elegans ortholog of Drp1. Using a strain of nematodes in which the endogenous DRP-1 protein is tagged with GFP, combined with MitoTracker Red staining to label mitochondria, we found that K21 induced the formation of larger and brighter DRP-1::GFP puncta in vivo, and that there was an

increase in the colocalization of these DRP-1::GFP puncta with MitoTracker Red-stained mitochondrial structures. These data comprise panels A-D of the new Figure 6, and support our assertion that K21 treatment recruits DRP-1 to mitochondria. Importantly, loss of function mutations in the drp-1 gene block the K21-induced fission, which we have added as new data in panels E-H of the new Figure 6. Taken together, these results provide compelling evidence that K21 promoted DRP-1-mediated mitochondrial fission.

5. Does K21 influence immune cell mitochondrial dynamics? It would be worth examining naïve m-CSF1-derived HMDM and perhaps T- cells to determine if they also have K21-inducible mitochondrial fission and mitophagy. This can be done using mitotracker staining or via IF staining against Tom20.

Authors' Response: We appreciate the reviewer's point, but as mentioned in response to the other reviewer above, HUVECs were chosen because of their easily scorable mitochondrial networks. By contrast, macrophage mitochondrial markers and morphology are rather poor, the cells are small and difficult to analyze using microscopy, and transfection into these cells is inefficient and often disruptive to their morphology, polarization phenotype, and function. Nevertheless, given the conservation of K21-induced mitophagy in C. elegans, we do not believe this physiological response is limited to macrophages but rather that most (perhaps all) cell types are induced to undergo mitophagy in response to K21. The MitoKeima marker in C. elegans provides direct evidence of mitophagy by monitoring the actual acidification of mitochondria as they enter the autophagosomal membrane system; consistent with this conclusion, mutations in lgg-1 (the nematode LC3β) block the acidification of MitoKeima-labeled mitochondria undergoing mitophagy, which we have added as new data in the new Figure 6.

To strengthen our case for mitophagy in human cells, we collaborated with the laboratory of Dr. Kay Macleod (University of Chicago), who is an established expert in studying mitophagy. Using a U2-OS cell line that stably expresses the fluorescent mitophagy reporter MitoQC, the Macleod lab showed that K21 treatment induces a similar acidification of mitochondria as seen in the MitoKeima experiments in C. elegans. This acidification is blocked by treatment with Bafilomycin A1, which inhibits the V-ATPase responsible for acidifying the autophagy and endolysosomal pathway. This is now in panel E of the new Figure 5.

To assess other mitophagy pathways, we focused on the two most studied: hypoxia-induced mitophagy, which can be induced with the hypoxia mimetic DFO and operates through the mitophagy receptor BNIP3/NIX, and decoupler-induced mitophagy, which can be induced with FCCP treatment and operates through the PINK1/Parkin system. Using two different cell types, we now clearly show that DFO-induced mitophagy and FCCP-induced mitophagy each have their specific molecular signatures (e.g., DFO causes an upregulation of BNIP3/NIX levels, whereas FCCP treatment causes a wide-spread lipidation of LC3β). It is well established that both mechanisms for mitophagy induction cause wide-spread cellular stress. By contrast, K21 treatment caused neither BNIP3/NIX upregulation nor systemic lipidation of LC3β. Rather, K21 treatment induced the stabilization and recruitment of Drp1/DRP-1 to mitochondria to induce fission in both human and C. elegans cells. This data can be found in Figure 5F and G. Our results suggest that K21 operates through a less stressful mechanism of mitophagy induction than that employed by DFO or FCCP treatment, but additional experiments to determine the specific mechanism go beyond the scope of this work.

Minor comments

1. Definition of HFFs needs to occur in first results paragraph as opposed to page 9

Authors' Response: The definition of HFF is now incorporated into the first paragraph of the Results (page 6, lines 152-155 of the new manuscript).

2. I appreciate the consistent Y axis scaling in Figure 1D to compare your macrophage sub populations, but it is difficult see and interpret the effects sizes like the one you're describing the activated M1 population. Can you either decrease the Y axis range or put a break in the Y axis of this panel?

Authors' Response: The previous Figure 1D is now Figure 2H in the new manuscript. By removing the HFF treatment data from the old Figure 1D and keeping the Y-axis flexible for each cell surface marker type, we now have a much cleaner figure in Figure 2H.

3. Some of the effects on protein expression in Figure 3D are not very clear. It would be appropriate to quantify band intensity normalised to your housekeeping protein control for each assessed protein.

Authors' Response: The previous Figure 3D is now Figure 5D in the new manuscript. We have now added the quantified and normalized protein values to the figure. Similar changes are also shown in other western blots (Figure 5G, 5H) within the manuscript.

4. Can you make dedicated method sections for "HMDM generation (please include your CD14 enrichment vs total monocyte approach) and HMDM polarisation (please include your methodology on HFF culture and co-culture with HMDM).

Authors' Response: We have now reorganized the Methods sections to clearly separate HMDM generation and polarization work from flow cytometry work.

5. The method section is two method sections put one after the other, presumably form the two labs. Please integrate your two methods sections and remove duplicate sections (statistics and data availability).

Authors' Response: The order of the individual Methods subsections is loosely matched to the order in which experiments are presented in the Results section to make it simpler for the reader to go back and forth between the two. As the human experiments comprise the first half of the Results and the C. elegans experiments the second half, we have organized the Methods subsections in the same order to match.

That said, the reviewer makes a good point regarding consolidating the two separate subsections on statistics and data availability for each organismal approach (and lab), which we have now merged in the new version of the Methods section.

6. Please provide references for description of GBP1, SOD2 and GBP5 protein functions on page 8.

Authors' Response: New references are added to the text.

7. In Extended data figure 13, can you have an image of your MitoKeima colocalising with mitotracker green in starved C. elegans? If the dye no longer works within the lysosome, perhaps IF detection of a mitochondrial protein?

Authors' Response: MitoTracker dyes work poorly as labels for mitolysosomes, as the reviewer surmised. Immunofluorescence protocols in C. elegans require treatments that disrupt the animal's cuticle to allow antibody permeability, and unfortunately these treatments damage mitochondrial morphology beyond recognition. An alternative is to use GFP as a tag, but GFP fluorescence is quenched by the low pH of the lysosome. We therefore generated a new strain in which we tagged the endogenous VDAC/Porin (VDAC-1 in C. elegans) with mNeonGreen, which is more resistant to acidic quenching than is GFP. We have done a colocalization analysis between the two excitation channels of MitoKeima and VDAC-1::mNeonGreen, showing colocalization between both in panels K-S on the new Supplemental Figure S4.

8. eat-2 line not described in the results section at all and extended data figure 16C-D aren't referenced at all?

Authors' Response: We apologize for this oversight. The purpose of examining eat-2 mutants was to provide a positive control for an animal that is undergoing starvation. EAT-2 encodes an ion channel expressed in the pharynx, and knockout mutants for eat-2 fail to pump their pharynx and feed correctly, resulting in a genetic model of calorie starvation, which is why we used it as a benchmark for what a starved animal with a feeding defect should look like. We have elaborated on this point in the Results section (page 15, line 470).

9. "Despite K21-treated animals showing similar feeding compared to untreated animals (Figure 4F)" I think this

refers to Extended Figure 16 and I think the statement is at odds with your observed increase with pharyngeal pumping. Can this be discussed and explained?

Authors' Response: The reviewer makes a fair point. Our focus was to show that K21 did not reduce feeding rates, which would be a simple explanation for some of the observed phenotypes induced by K21. We did not see a decrease in feeding rates (i.e., pharyngeal pumping rates) with K21 treatment. Indeed, there was a slight increase in the rates of K21-treated worms compared to untreated animals. Although we currently don't know the underlying mechanism behind this subtle increase in pumping rates, one explanation could be increased mitochondrial efficacy of the treated animals, leading to increased motor activity of the pharyngeal organ. While quite interesting, this is a tricky point to test and goes beyond the scope of the paper. We have rephrased the statement to indicate that K21 treatment doesn't cause a drop in pumping rates, as would be expected if it impaired feeding (page 15, lines 469). We have also highlighted this result in the Discussion (page 22, lines 672-677).

10. In text referencing for Extended Figures 11 and onwards lack reference to specific figure panels.

Authors' Response: We apologize for this oversight. We have made sure that all references to figures in the text include specific panels.

11. Statistical analyses in Figure 1B&D, Extended Figure 1B, Extended Figure 2A&C, should be performed using two-way ANOVA with a post hoc statistical test as opposed to unpair T-tests.

Authors' Response: Suggested changes have been made to the figures.

12. Statistical analysis in Figure 3C should be performed using a one-way ANOVA with a post hoc statistical test.

Authors' Response: Suggested changes have been made to the old Figure 3C, which is now new Figure 5A, 5B.

Reviewer #3 (Significance (Required)):

Significance

The paper significance is drawn from the findings that K21 influences macrophage cell identity and presumably function. The finding that K21 influences mitochondrial dynamics and promotes mitophagy was identified in cell lines and in vivo using C. elegans. There is however a disconnect as there is no functional data that K21 is boosting macrophage antimicrobial function or if the effect on mitochondria contributes to observed chances in macrophage polarisation. The observed effects on longevity in C. elegans are interesting but quite disconnected from immunological scope at the start of the paper. A lot of the previous work into K21 relates to dentistry and wound healing, so this paper provides development into innate immunology and some conceptual advances in its role in immune cell regulation. Audiences interested in this paper would be split between immunologists interested in developing host directed therapies and mitochondrial/mitophagy biologists interested in longevity and mitochondrial health/maintenance. As a reviewer, my research experience is in macrophage biology pertaining to inflammatory and antimicrobial responses downstream of metabolic rewiring. I have some experience collaborating with a C. elegans laboratory but my input to that section of this manuscript is limited.

Authors' Response: We appreciate the reviewer's assessment of the significance of the study. We have re-written the manuscript by expanding our discussion on mitochondrial health, including what is known about the links between tissue homeostasis and mitochondrial quality. We have also added some additional experiments (the new Figure 5 and Figure 6, plus Supplementary Figures S3 and S4) to flesh out the changes in mitochondria in response to K21, including comparing K21-induction of mitophagy to induction by DFO treatment and FCCP treatment, demonstrating an increase Drp1/DRP-1 stabilization and mitochondrial recruitment in both human cells and C. elegans cells, the use a pH-based mitophagy reporter (MitoQC) in cultured mammalian cells, and a clear demonstration that mitophagy induction by K21 requires the autophagy receptor LC3β/LGG-1. We have

added experiments that directly test the effects on known mitophagy inducers on the ability of the innate immune system to combat a model bacterial pathogen (the new Supplementary Figure S3).

Finally, we have highlighted in the Results and the Discussion the rationale and importance of examining K21 in C. elegans and comparing its effect on that model system versus human cells to make that connection more obvious and meaningful to the reader. Why compare the effects of K21 on C. elegans versus PBMCs? If the target of K21 action is mitochondria and their quality control mechanisms, then the effects of K21 observed in human tissue culture cells should be deeply conserved across animal phyla, as well as observed in a whole-animal model. Our C. elegans experiments satisfied both criteria, demonstrating an in vivo context shared by our observation of the effects of K21 on PBMCs in culture. Nematodes do not possess macrophages or other well-established components of human innate and adaptive immunity; nevertheless, genes associated with innate immunity in C. elegans were upregulated by K21 treatment. Although the effects of K21 on immunity may not be analogous to those in humans, we found that K21 treatment extended the lifespan of C. elegans without compromising survival or fertility, consistent with increased activity of mitophagy as a quality control mechanism that boosts health through hormesis. As nematodes and humans are separated by nearly a billion years of evolution, the overall conserved effects of K21 suggest that the molecular or pharmacological target of K21 is deeply conserved and not specific to immune cells. This evolutionary conservation (and with an in vivo system) supports the reliability of the findings and elevates the conclusions from the work above more typical experiments using drugs on cultured cells.

We feel that the expanded description of the experiments in the text combined with the additional experiments performed to address the reviewers' concerns provide the needed support for the proposed mechanistic and functional conclusions. Our submitted manuscript is a detailed , thorough, and dense first step towards understanding the mechanism of K21 action, and the conservation of its effects on mitochondrial fission and mitophagy both in vitro and in vivo – across a billion years of evolution – provide strong evidence for its mechanism of action, with enough significant impact on our understanding to merit publication at this stage of research.

August 20, 2026

RE: Life Science Alliance Manuscript #LSA-2026-03852-T

Prof. Bhupesh Kumar Prusty
Riga Stradins University
Institute for Microbiology and Virology
Kleisti Science Hub, Ratsupites ieal 5
Riga LV-1067
Latvia

Dear Dr. Prusty,

Thank you for submitting your manuscript entitled "Drug-induced metabolic remodeling of immune cells generates an effective antimicrobial effect". Your manuscript was returned to two of the original Review Commons referees, whose comments are below. As you will see both referees commend the substantial improvements to this work, and Referee 1 supports publication while Referee 3 requests few minor improvements. We concur with their first point that a higher resolution inset of images in Figure 2B are merited. Per their second point, phospho-Drp1 appears not to be shown in relevant western blots. Furnishing new western blot data is optional, but at minimum the text should remark that phospho-Drp1 was not measured here. Demonstrating a requirement for Drp1 activity for C elegans lifespan extension would cement its role here but this is optional. We would be happy to publish your paper in Life Science Alliance pending these final changes and revisions necessary to meet our formatting guidelines.

MANUSCRIPT ORGANIZATION AND FORMATTING:

To avoid unnecessary delays in the acceptance and publication of your paper, please read the following information carefully. Full guidelines are available on our Instructions for Authors page, https://www.life-science-alliance.org/authors

Submission of a paper that does not conform to Life Science Alliance guidelines will delay the acceptance of your manuscript.

-Please add a Summary Blurb/Alternate Abstract and Category for your manuscript in our system.
-Please add the X and Bluesky handles of your host institute/organization, as well as your own and/or one of the authors, in our system.
-While the current title is acceptable we suggest a reference to innate immunity or myeloid cells instead of simply "immune cells". We further suggest incorporating longevity into the title and/or some reference to the C elegans observations, such as: "Drug-induced metabolic remodeling promotes antimicrobial activity in myeloid cells and extends longevity in vivo"
Finally please ensure the title is consistent in both the system and the manuscript file.
-Please remove the phrase "in vivo" from the abstract as it is redundant in this context.
-Please be sure that the authorship listing and order are correct and match between the system and manuscript file.
-Please mark the secondary Corresponding Author in our system and be sure they have connected their ORCID iD with their account. Detailed instructions have been sent to them in a separate email.
-Please add the authors' contributions to the system as well.
-Please rename "COMPETING INTEREST DECLARATION" to "Conflict of Interest".
-Please add callouts for Figure 6I, J to your main manuscript text.
-The methods section is inconsistent in the level of detail provided on microscopy. Please ensure all details on imaging including illumination, software, and image processing are included for macrophages (rather than referring to a prior study) as are already in place for C elegans data. Please also provide concentrations of all antibodies used for flow cytometry.

We encourage our authors to provide original source data, particularly uncropped/-processed electrophoretic blots and spreadsheets for the main figures of the manuscript. If you would like to add source data, we would welcome one PDF/Excel-file per figure for this information. These files will be linked as supplementary "Source Data" files.

It is Life Science Alliance policy that if requested, original data images must be made available to the editors. Failure to provide original images upon request will result in unavoidable delays in publication. Please ensure that you have access to all original data images prior to final submission.

If you are planning a press release on your work, please inform us immediately to allow informing our production team and scheduling a release date.

We welcome submissions of potential cover images for the issue of LSA in which your work would appear. If you have high

quality images associated with this work, please feel free to email these, with a caption, to the journal office.

LSA encourages authors to provide a 30-60 second video where the study is briefly explained. These videos will be appear embedded with the manuscript online at Life Science Alliance, and on social media to promote the published paper and authors (for examples, see https://docs.google.com/document/d/1-UWCfbE4pGcDdcgzcmiuJl2XMBJnxKYeqRvLLrLSo8s/edit?usp=sharing). Corresponding or first-authors are welcome to submit the video. Please submit only one video per manuscript. The video can be emailed to contact@life-science-alliance.org

FINAL FILES:

To upload the final version of your manuscript, please log in to your account: https://lsa.msubmit.net/cgi-bin/main.plex
You will be guided to complete the submission of your revised manuscript and to fill in all necessary information. Please get in touch in case you do not know or remember your login name.

The following items are required for acceptance.

-- An editable version of the final text (.DOC or .DOCX) is needed for copyediting (no PDFs).

-- High-resolution figure, supplementary figure and video files uploaded as individual files: See our detailed guidelines for preparing your production-ready images, https://www.life-science-alliance.org/authors

-- Summary blurb (enter in submission system): A short text summarizing in a single sentence the study (max. 200 characters including spaces). This text is used in conjunction with the titles of papers, hence should be informative and complementary to the title. It should describe the context and significance of the findings for a general readership; it should be written in the present tense and refer to the work in the third person. Author names should not be mentioned.

The license to publish form must be signed before your manuscript can be sent to production. A link to the license to publish form will be available to the corresponding author only. Please take a moment to check your funder requirements.

Reviews, decision letters, and point-by-point responses associated with peer-review at Life Science Alliance will be published, alongside the manuscript. If you do want to opt out of having the reviewer reports and your point-by-point responses displayed, please let us know immediately.

Thank you for your attention to these final processing requirements. Please revise and format the manuscript and upload materials as soon as you are able.

Thank you for this interesting contribution to the literature. We look forward to publishing your paper in Life Science Alliance.

Sincerely,

Tim Fessenden
Scientific Editor
Life Science Alliance

Reviewer #1 (Comments to the Authors (Required)):

In this study, the authors investigated the effects of K21, a silane derivative previously reported to have broad antimicrobial activity, on macrophage differentiation and activation. They found that K21 alters cytokine secretion and cell-surface protein expression, selectively promoting the expansion of pro-inflammatory macrophages. They also showed that K21 enhances mitochondrial health in peripheral blood mononuclear cells by stimulating mitophagy. Finally, K21 induced mitophagy and extended lifespan in C. elegans.

The authors have addressed my concerns by providing new data and revising the manuscript accordingly. I have no further concerns, and I consider the manuscript suitable for publication in Life Science Alliance.

Reviewer #3 (Comments to the Authors (Required)):

Hollman et al have investigated the immunomodulatory role of the antimicrobial agent K21 in human cells (immune cells and cell lines) and C. elegans. They found that administration of K21 to differentiating macrophages influences macrophage polarisation states and ultimately function. They find that K21 promotes mitochondrial fission and subsequently mitophagy in human cell

models. Their findings extend to C. elegans models where this response is evolutionarily conserved. The nematode model provides mechanistic detail about the involvement of mitochondrial fission protein DRP1. They observed that this K21-driven mitophagy promoted nematode survival and identified conserved gene expression responses between nematode and macrophage that could be driving the K21-specific benefits in both systems.

The data presented are strongly supportive of their main points and upon revision the authors have provided adequate additional experiments and technical details to address most of my concerns. I have a few more questions that I think are worth addressing:

1. The additional gentamicin exclusion infection assays support the model. It would be helpful to determine macrophage viability post infection. Excessive macrophage cell death and release of bacteria into the gentamicin-containing supernatant prior to your PBS washes and lysis could have considerable impact on bacterial CFU. I ask since there was such low bacterial retrieval in M1 macrophages. I think this is addressed in your images in Figure 2B, but the resolution makes it hard to tell. If this needs to be assessed further, I would recommend an LDH assay of supernatants from infected macrophages at your 4 h.p.i collection time point.

2. Authors have investigated direct DRP1 involvement in response to comments from me and the other reviewers. In the response to my comments, they mention changes in phospho-DRP1 levels, but I don't think this has been included in the final figures. If you have them, they're probably worth including, along with your explanations regarding alternative methods of DRP1 activation.

3. Did you perform any mitokeima experiments in the drp1 C. elegans following K21 administration? Its inferred, and quite likely, that the Drp1-driven fission is required for K21-inducible mitophagy, this experiment would prove it.

4. Supplementary Figures 7 and 8 are swapped - please amend the figure order to match the body text.

Point-by-point response to the editorial and reviewer comments:

EDITORIAL COMMENTS:

Your manuscript was returned to two of the original Review Commons referees, whose comments are below. As you will see both referees commend the substantial improvements to this work, and Referee 1 supports publication while Referee 3 requests few minor improvements. We concur with their first point that a higher resolution inset of images in Figure 2B are merited. Per their second point, phospho-Drp1 appears not to be shown in relevant western blots. Furnishing new western blot data is optional, but at minimum the text should remark that phospho-Drp1 was not measured here. Demonstrating a requirement for Drp1 activity for C elegans lifespan extension would cement its role here but this is optional. We would be happy to publish your paper in Life Science Alliance pending these final changes and revisions necessary to meet our formatting guidelines.

Authors' Response:

We thank the editor and all reviewers for their constructive review, which has helped us improve the overall quality of the manuscript. We agree with the editor's points and have incorporated the following changes into the revised manuscript.

1. We have added high-resolution insets to Figure 2B. We also mentioned this in the figure legend.
2. Although technically challenging, we have visualized K21-induced changes in mitochondrial dynamics in our PBMC-derived macrophages, as requested by reviewers. We have included this data as the new Supplemental Figure S4.
3. We were unable to test pDrp1 levels in our blots because a suitable pDrp1 antibody was unavailable. We mentioned this in the text (lines 339-341).
4. We have not examined K21-induced lifespan extension in *drp-1* mutants. Mutations in *drp-1* reduce fertility, and reduced fertility by itself alters lifespan. Moreover, the role of DRP-1 in *C. elegans* mitophagy (or Drp1 in mammalian mitophagy for that matter) has not been explored extensively and would require an analysis that goes beyond the scope of the current manuscript.

MANUSCRIPT ORGANIZATION AND FORMATTING:

-Please add a Summary Blurb/Alternate Abstract and Category for your manuscript in our system.

Authors' Response:
A summary Blurb and Category of the manuscript have now been added to the LSA manuscript submission system.

-Please add the X and Bluesky handles of your host institute/organization, as well as your own and/or one of the authors, in our system.

Authors' Response:
X and Bluesky handles of both the institutions (Riga Stradins University and Rutgers University), as well as X handles of both the corresponding authors, have been added to the LSA manuscript submission system.

-While the current title is acceptable we suggest a reference to innate immunity or myeloid cells instead of simply "immune cells". We further suggest incorporating longevity into the title and/or some reference to the C elegans observations, such as:
"Drug-induced metabolic remodeling promotes antimicrobial activity in myeloid cells and extends longevity in vivo"

Authors' Response:
The manuscript title has now been changed. The new title is "Drug-induced metabolic remodeling promotes antimicrobial activity in macrophages and extends longevity in vivo".

Finally please ensure the title is consistent in both the system and the manuscript file.

Authors' Response:
Title consistency has now been verified.

-Please remove the phrase "in vivo" from the abstract as it is redundant in this context.

Authors' Response:
The word 'In vivo' has been removed from the abstract.

-Please be sure that the authorship listing and order are correct and match between the system and manuscript file.

Authors' Response:
We have edited the authorship listing and order in the LSA manuscript submission system to match the manuscript file.

-Please mark the secondary Corresponding Author in our system and be sure they have connected their ORCID iD with their account. Detailed instructions have been sent to them in a separate email.

Authors' Response:
We have made the necessary changes to rectify these issues.

-Please add the authors' contributions to the system as well.

Authors' Response:
We have made the necessary changes to rectify these issues.

-Please rename "COMPETING INTEREST DECLARATION" to "Conflict of Interest".

Authors' Response:
We have made the necessary changes to rectify these issues.

-Please add callouts for Figure 6I, J to your main manuscript text.

Authors' Response:
We have made appropriate changes within the text to mention Figure 6I- 6J (line 473-474).

-The methods section is inconsistent in the level of detail provided on microscopy. Please ensure all details on imaging including illumination, software, and image processing are included for macrophages (rather than referring to a prior study) as are already in place for C elegans data. Please also provide concentrations of all antibodies used for flow cytometry.

Authors' Response:
We have edited the Methods section to provide more details on antibody dilutions used for flow cytometry, as well as the imaging methodologies.

We encourage our authors to provide original source data, particularly uncropped/-processed electrophoretic blots and spreadsheets for the main figures of the manuscript. If you would like to add source data, we would welcome one PDF/Excel-file per figure for this information. These files will be linked as supplementary "Source Data" files.

Authors' Response:
We have now provided Source files for all the main figures. We have also provided PDF files with uncropped gel images for all the Western blots shown within the main figure.

It is Life Science Alliance policy that if requested, original data images must be made available to the editors. Failure to provide original images upon request will result in unavoidable delays in publication. Please ensure that you have access to all original data images prior to final submission.

Authors' Response:
We are happy to provide original data images to the editor upon request.

We welcome submissions of potential cover images for the issue of LSA in which your work would appear. If you have high quality images associated with this work, please feel free to email these, with a caption, to the journal office.

Authors' Response:

We have selected a super-resolution microscopy image of mitochondria undergoing mitophagy in *C. elegans in vivo*. The color image shows mitochondria labelled with VDAC-1::mNeonGreen (decorating the outer mitochondrial membrane in yellow) and mitophagosomes (red) labelled with the acidified version of MitoKeima.

*LSA encourages authors to provide a 30-60 second video where the study is briefly explained. These videos will be appear embedded with the manuscript online at Life Science Alliance, and on social media to promote the published paper and authors (for examples, see https://docs.google.com/document/d/1-UWCfbE4pGcDdcqzcmiuJl2XMBJnxKYeqRvLLrLSo8s/edit?usp=sharing). Corresponding or first-authors are welcome to submit the video. Please submit only one video per manuscript. The video can be emailed to contact@life-science-alliance.*org

Authors' Response:
We have submitted a 90-second video via a separate email to *contact@life-science-alliance.*org

----

Reviewer #1 (Comments to the Authors (Required)):

In this study, the authors investigated the effects of K21, a silane derivative previously reported to have broad antimicrobial activity, on macrophage differentiation and activation. They found that K21 alters cytokine secretion and cell-surface protein expression, selectively promoting the expansion of pro-inflammatory macrophages. They also showed that K21 enhances mitochondrial health in peripheral blood mononuclear cells by stimulating mitophagy. Finally, K21 induced mitophagy and extended lifespan in C. elegans.

The authors have addressed my concerns by providing new data and revising the manuscript accordingly. I have no further concerns, and I consider the manuscript suitable for publication in Life Science Alliance.

Authors' Response:
We thank the reviewer for the in-depth review and constructive comments.

Reviewer #3 (Comments to the Authors (Required)):

1. The additional gentamicin exclusion infection assays support the model. It would be helpful to determine macrophage viability post infection. Excessive macrophage cell death and release of bacteria into the gentamicin-containing supernatant prior to your PBS washes and lysis could have considerable impact on bacterial CFU. I ask since there was such low bacterial retrieval in M1 macrophages. I think this is addressed in your images in Figure 2B, but the resolution makes it hard to tell. If this needs to be assessed further, I would recommend an LDH assay of supernatants from infected macrophages at your 4 h.p.i

collection time point.

Authors' Response:
We appreciate the reviewer's concerns. To address this, we added low-magnification images of infected cells in Figure 2B. We have now added high-resolution insets to Figure 2B. We also mentioned this in the figure legend. We appreciate the reviewer's recommendation to perform an LDH assay on supernatants from infected macrophages; however, we do not see the need for this experiment here given no signs of such cell death were observed in the higher-magnification images, and as it would take a dramatic amount of time to complete this additional analysis.

2. Authors have investigated direct DRP1 involvement in response to comments from me and the other reviewers. In the response to my comments, they mention changes in phospho-DRP1 levels, but I don't think this has been included in the final figures. If you have them, they're probably worth including, along with your explanations regarding alternative methods of DRP1 activation.

Authors' Response:
We were unable to test phosphor-Drp1 levels in our blots because a suitable antibody against pDrp1 was unavailable. We mentioned this in the text (lines 339-341).

3. Did you perform any mitokeima experiments in the drp1 C. elegans following K21 administration? Its inferred, and quite likely, that the Drp1-driven fission is required for K21-inducible mitophagy, this experiment would prove it.

Authors' Response:
We have not examined K21-induced lifespan extension in *drp-1* mutants. Mutations in *drp-1* reduce fertility, and reduced fertility by itself alters lifespan, complicating interpretation of this experiment. Moreover, the role of DRP-1 in *C. elegans* mitophagy (or Drp1 in mammalian mitophagy for that matter) has not been explored extensively and would require an analysis that goes beyond the scope of the current manuscript.

4. Supplementary Figures 7 and 8 are swapped - please amend the figure order to match the body text.

Authors' Response:
We would like to note that Supplementary Figures S7 and S8 were in reverse order in the first version of the manuscript. However, these two figures are in the correct order (new Supplementary Figure S8 and S9) within the revised manuscript and are described correctly in the text.

September 1, 2026

RE: Life Science Alliance Manuscript #LSA-2026-03852-TR

Prof. Bhupesh Kumar Prusty
Riga Stradins University
Institute for Microbiology and Virology
Kleisti Science Hub, Ratsupites ieal 5
Riga LV-1067
Latvia

Dear Dr. Prusty,

Thank you for submitting your revised Research Article entitled "Drug-induced metabolic remodeling promotes antimicrobial activity in macrophages and extends longevity in vivo". We note the changes in place that resolve remaining reviewer concerns and journal requirements, as well as new observations on mitochondrial localization of Drp1 and LC3b in M2 macrophages. It is a pleasure to let you know that your manuscript is now accepted for publication in Life Science Alliance. Congratulations on bringing together these two experimental systems to set forth a unique and valuable advance on innate immunity and mitochondria.

The final published version of your manuscript will be deposited by us to PubMed Central upon publication.

Your manuscript will now progress through copyediting and proofing. It is journal policy that authors provide original data upon request.

Your article will publish open access upon publication under a CC-BY license.

Reviews, decision letters, and point-by-point responses associated with peer-review at Life Science Alliance will be published, alongside the manuscript. If you do want to opt out of having the reviewer reports and your point-by-point responses displayed, please let us know immediately.

IMPORTANT: If you will be unreachable at any time, please provide us with the email address of an alternate author. Failure to respond to routine queries may lead to unavoidable delays in publication.

Scheduling details will be available from our production department. You will receive proofs shortly before the publication date. Only essential corrections can be made at the proof stage so if there are any minor final changes you wish to make to the manuscript, please let the journal office know now.

DISTRIBUTION OF MATERIALS:
Authors are required to distribute freely any materials used in experiments published in Life Science Alliance. Authors are encouraged to deposit materials used in their studies to the appropriate repositories for distribution to researchers.

You can contact the journal office with any questions, contact@life-science-alliance.org

Again, congratulations on a very nice paper. I hope you found the editorial process to be constructive and are pleased with how the manuscript was handled. We look forward to future exciting submissions from your lab.

Sincerely,

Tim Fessenden
Scientific Editor
Life Science Alliance
